# Enzymic recognition of amino acids drove the evolution of primordial genetic codes

**DOI:** 10.1093/nar/gkad1160

**Published:** 2023-12-04

**Authors:** Jordan Douglas, Remco Bouckaert, Charles W Carter, Peter R Wills

**Affiliations:** Department of Physics, The University of Auckland, New Zealand; Centre for Computational Evolution, The University of Auckland, New Zealand; Centre for Computational Evolution, The University of Auckland, New Zealand; School of Computer Science, The University of Auckland, New Zealand; Department of Biochemistry and Biophysics, University of North Carolina at Chapel Hill, USA; Department of Physics, The University of Auckland, New Zealand; Centre for Computational Evolution, The University of Auckland, New Zealand

## Abstract

How genetic information gained its exquisite control over chemical processes needed to build living cells remains an enigma. Today, the aminoacyl-tRNA synthetases (AARS) execute the genetic codes in all living systems. But how did the AARS that emerged over three billion years ago as low-specificity, protozymic forms then spawn the full range of highly-specific enzymes that distinguish between 22 diverse amino acids? A phylogenetic reconstruction of extant AARS genes, enhanced by analysing modular acquisitions, reveals six AARS with distinct bacterial, archaeal, eukaryotic, or organellar clades, resulting in a total of 36 families of AARS catalytic domains. Small structural modules that differentiate one AARS family from another played pivotal roles in discriminating between amino acid side chains, thereby expanding the genetic code and refining its precision. The resulting model shows a tendency for less elaborate enzymes, with simpler catalytic domains, to activate amino acids that were not synthesised until later in the evolution of the code. The most probable evolutionary route for an emergent amino acid type to establish a place in the code was by recruiting older, less specific AARS, rather than adapting contemporary lineages. This process, retrofunctionalisation, differs from previously described mechanisms through which amino acids would enter the code.

## Graphical Abstract

**Graphical Abstract:**
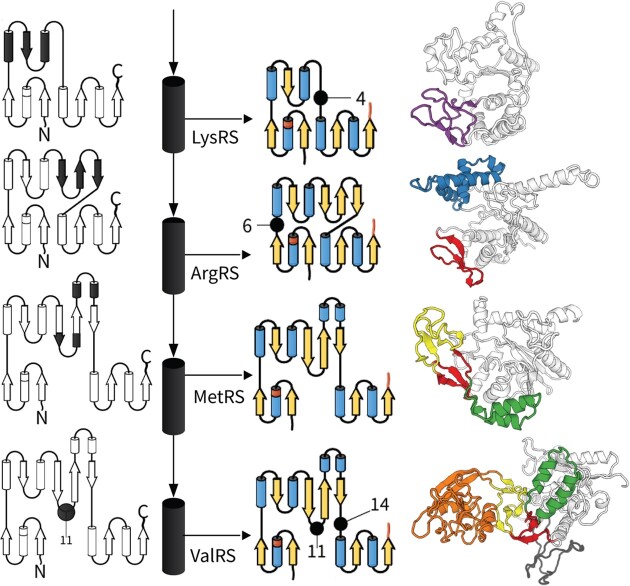

## Introduction

The primordial genetic codes would have looked significantly different from their contemporary descendants (1,2). Whereas the genetic codes of today are almost deterministic and include up to 22 amino acids, the primordial genetic codes would have been ambiguous due to low translational fidelity (3,4) and used the limited pool of amino acids initially available for protein synthesis. Some amino acids were available through prebiotic geochemistry and simple metabolic pathways, but there would be no enrichment of the more complex or less stable molecules until protocellular metabolism and regulation had advanced sufficiently (5–9). Further, the first genetic codes were likely geographically regional. Protocellular populations may have resided in different parts of the ocean or in confined aqueous environments (10,11), but still able to exchange genetic material periodically. Under the error minimisation theory, these competing genetic codes were selected for their ability to dampen the effect of genetic mutation on protein structure and function (2). With time, translational fidelity sharpened, the pool of amino acids diversified, and the pairing between amino acids and anticodons was optimised—offering the genetic code greater precision, utility and robustness. Protocellular complexity grew to a tipping point where changes to the genetic code would become incremental and rare (12), giving the appearance of a ‘frozen accident’ (13).

In all contemporary living things, genetic coding is effected by the catalytic action of aminoacyl-tRNA synthetases (AARS), a large group of enzymes that attach amino acids to their cognate tRNA. Aminoacylation is a two-step reaction powered by adenosine triphosphate (ATP). These two steps involve, first, activating an amino acid by attaching it to adenosine monophosphate, and second, charging its cognate tRNA with the amino acid.

Any comprehensive explanation of the origin of the genetic code, a subject of considerable debate (see reviews: (1,3)), must pay close attention to the AARS. The RNA world hypothesis suggests that the genetic code originated in an environment where self-reproducing populations of diverse RNA governed life’s reaction pathways, including aminoacylation through hypothetical ribozymal aminoacyl-tRNA synthetases (1,4), which have been synthesised by a number of laboratories (14–16), but have not been observed in nature. Ribozymes would later be supplanted by proteinaceous enzymes due to their superior catalytic properties. AARS enzymes are an afterthought in the RNA world version of the code’s origin. Nucleopeptide world challenges this classical theory. It proposes the genetic code originated in an environment which supported the RNA catalysis of peptide synthesis, and peptide catalysis of RNA synthesis, with AARS serving the central integrating role (1,3,17) as these enzymes now do in all three domains of life – bacteria, archaea, and eukaryota – as well as mitochondria and chloroplasts.

Contemporary AARS are curious enzymes, rife with idiosyncrasies (see review: (18)). They consist of a catalytic domain which recognises an amino acid (and ATP), one or more domains that recognise tRNA (typically its acceptor stem and anticodon), and sometimes an editing domain that expels mistargeted amino acids from the reaction pathway. AARS belong to two distinct, apparently unrelated, evolutionary groups, which are designated Class I and Class II. The majority of within-class diversification likely occurred before the last universal common ancestor (LUCA) (3,9). Nine of the 22 proteinogenic amino acids are rendered into proteins from tRNAs charged exclusively by Class I enzymes, eleven by Class II, and the remaining two amino acids, lysine (19) and cysteine (20), can be rendered from the products of Class I or II analogs. In most cases, each AARS attaches a single amino acid type to its code-cognate tRNA, specified in the naming of that enzyme - for example alanyl-tRNA synthetase (AlaRS) attaches alanine onto tRNA^Ala^. However, in some cases, an AARS supplies an additional amino acid through pretranslational modification of the original amino acid substrate after its attachment to tRNA. This is the case for the non-discriminating aspartyl- and glutamyl-tRNA synthetases (AsxRS and GlxRS), which attach Asp to tRNA^Asn^ and Glu to tRNA^Gln^, respectively (21,22). Similarly, O-phosphoseryl-tRNA synthetase (SepRS) supplies cysteine for organisms lacking CysRS (20), and SerRS supplies both serine and selenocysteine (23). There is also a discriminating GluRS that attaches Glu to tRNA^Gln^, representing an ancestral midpoint between discriminating and non-discriminating forms (24,25).

The Class I AARS catalytic domain is characterised by a Rossmann fold containing a four-stranded parallel β-sheet, and Class II by a six-stranded antiparallel sheet (18). But while there are just two evolutionary superfamilies of catalytic domains (Classes I and II), there are several superfamilies of domains that recognise tRNA molecules (26), and these have a history of exchanging between enzymes as mobile elements (19,27). Indeed, these domains are often auxiliary to tRNA recognition elements found in the catalytic domain (28,29), which are specific to different families (27,30–32). Due to the central role of the catalytic domain in recognising both amino acids and tRNA acceptor stems, and the comparatively fluid nature of tRNA anticodon recognition, we restrict our focus to the catalytic domains.

We combine information from both sequence and structure using a phylogenetic method within a Bayesian framework. To that end, we assembled a taxonomically representative dataset of AARS structural predictions to recover a ‘snapshot of the tree of life’. We identified structural elements common to either class, and the insertion modules (IM) that characterise subclasses and families. These insertion modules define a succession of AARS catalytic domain families. This succession suggests a piecewise assembly of aminoacyl-tRNA synthetases through evolutionary time and demonstrates how the model explains key aspects of genetic code evolution. Although preexisting AARS phylogenetic analyses are manifold (26,33–36), this study stands alone in its use of (i) a comprehensive and taxonomically representative dataset, (ii) a Bayesian phylogenetic method which accounts for changes in both sequence and structural modules and (iii) an interpretation of the resulting phylogeny that describes the assembly of protein structures over evolutionary time. We hope this synthesis of modular and sequence phylogeny will contribute to an eventual understanding of how protein folding coevolved with the growth of the coding table itself.

## Materials and methods

### Building sequence alignments

**Figure 1.**
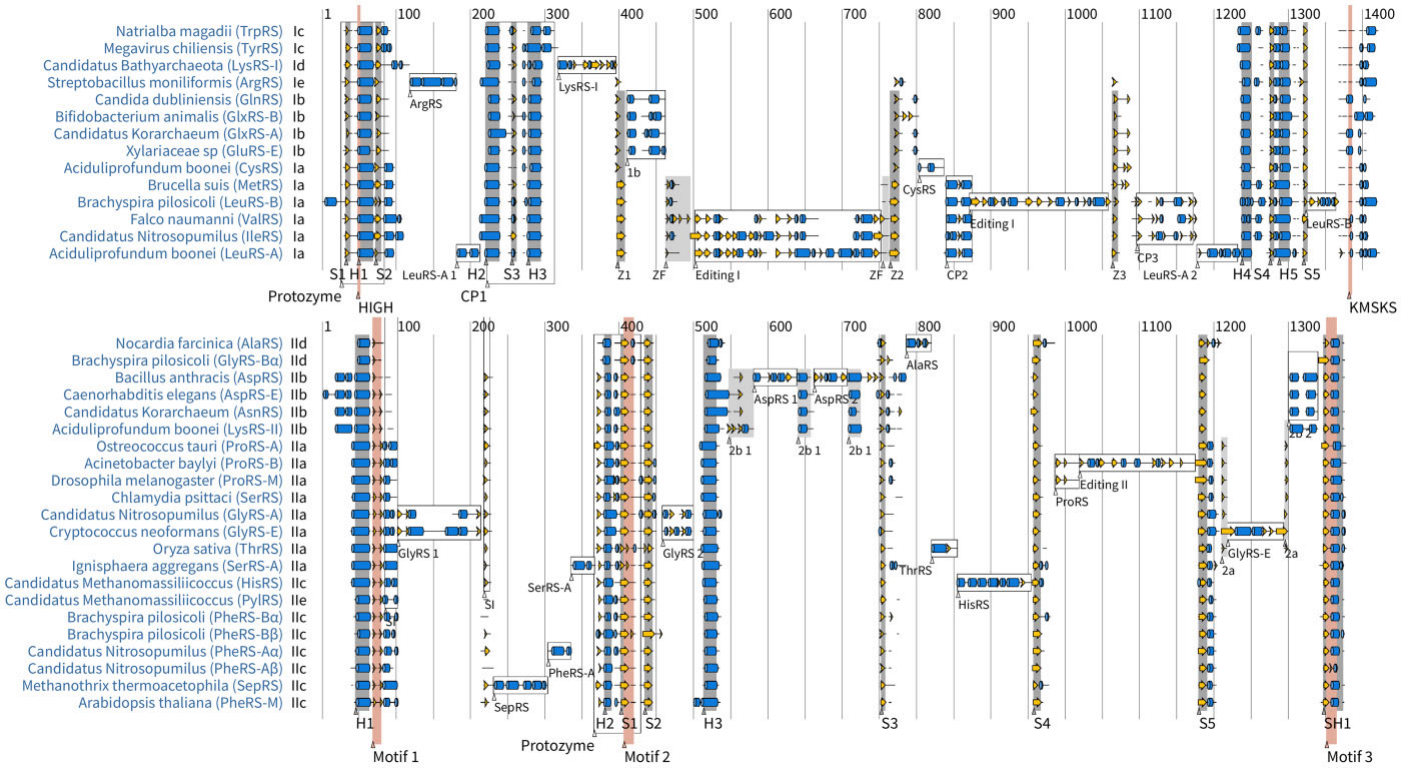
Multiple sequence alignment of Class I (top) and Class II (bottom) catalytic domains. One AlphaFold-generated representative was randomly selected from each family, provided that the reference structure contained all of the insertion modules which characterise the family. Helices are depicted by blue cylinders; β-strands by yellow arrows; all other secondary structural elements by black lines; and multiple sequence alignment gaps are left blank. For simplicity, when an extended helix or strand is interrupted by a single secondary structural element (such as a turn or a bend), that element is omitted from the diagram.

Annotated AARS sequence entries were searched for on GenBank using the rentrez library (37), and the taxonomically-representative samples of each family were selected randomly from the downloaded sequences. Protein structures were predicted with AlphaFold v2.3.0 (38) and secondary structures were defined using DSSP v3.0.0 (39). Protein structures were displayed using PV (Marco Biasini. (2015). pv: v1.8.1. Zenodo. 10.5281/zenodo.20980). Pairwise structural alignments were generated by DeepAlign (40). Per-family multiple sequence alignments were generated by first aligning the structures with 3DCOMB (41), followed by a refinement algorithm that realigned contiguous regions of at least three sites lacking secondary structure, using ClustalW based on primary sequence (42). This protocol was especially useful for aligning the flexible region flanking the Class I KMSKS motif. As existing structural alignment tools were not always reliable at delineating homologous insertions, alignments were treated to manual adjustment. To keep the superfamily alignment problem tractable, only one representative of each family was used (see Figure 1), and one alignment was generated per class using the protocol above, and then the family alignments were incorporated into the superfamily alignment afterwards.

### Bayesian phylogenetic inference

All phylogenetic analyses were performed using BEAST v2.7.3 (43). Two independent Markov chain Monte Carlo chains were run for each class, and their convergence was assessed by confirming their effective sample sizes were over 200 using Tracer v1.7 (44). Trees were summarised using the maximum clade credibility tree (45) and visualised using UglyTrees (46). AARS families were identified using the optimised relaxed clock (v1.1.1) (47), the OBAMA substitution model (v1.1.1) (48), and the BICEPS tree prior (v1.1.1) (49). After the AARS families were identified, they were constrained in all subsequent analyses by assigning them to protein families (as opposed to *species*) in the multispecies coalescent model (50) implemented in StarBeast3 (v1.1.7) (51). To calculate the ancestral frequencies of phase II amino acids (Figure 5), we used BEAST 2 to perform ancestral reconstruction, with 4 rate categories, and gap characters modelled as the 21st amino acid. This analysis was performed on multiple sequence alignments of the common elements of the Class I and II catalytic domains, as described in Supporting information. The frequency of phase II amino acids for the ancestor of each AARS family was compared with the empirical frequency averaged across all extant members of that family.

**Figure 2.**
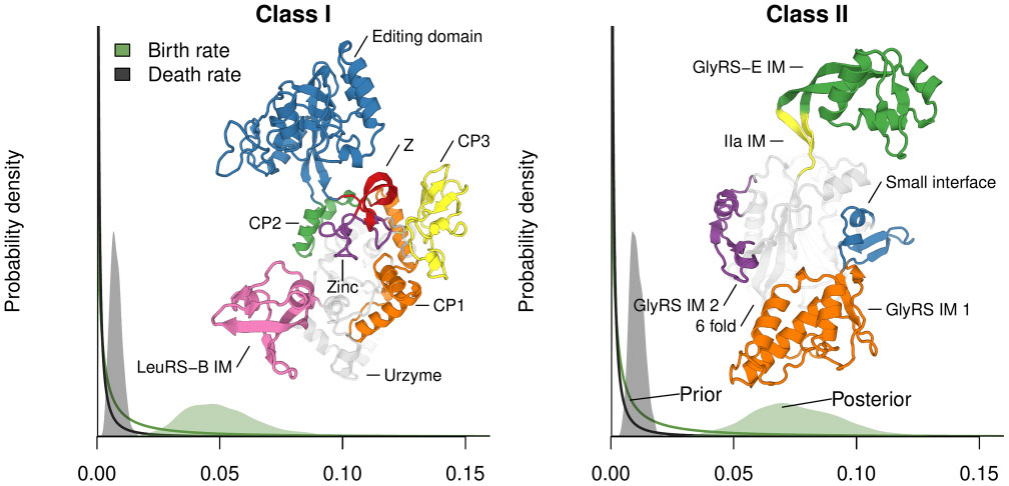
Prior and posterior distributions of birth and death rates of IMs, relative to amino acid substitution rate. Protein structures are the catalytic domains of the *Thermus thermophilus* LeuRS-B (PDB: 2V0C (107)) and the *Cryptococcus neoformans* GlyRS-E (generated by AlphaFold). The GlyRS-E IM is intrinsically disordered (32), and therefore its predicted structure above (green) may be one of the many conformations it adopts. It exists as an insertion nested within the β-hairpin found in most members of *IIa* (yellow).

**Figure 3.**
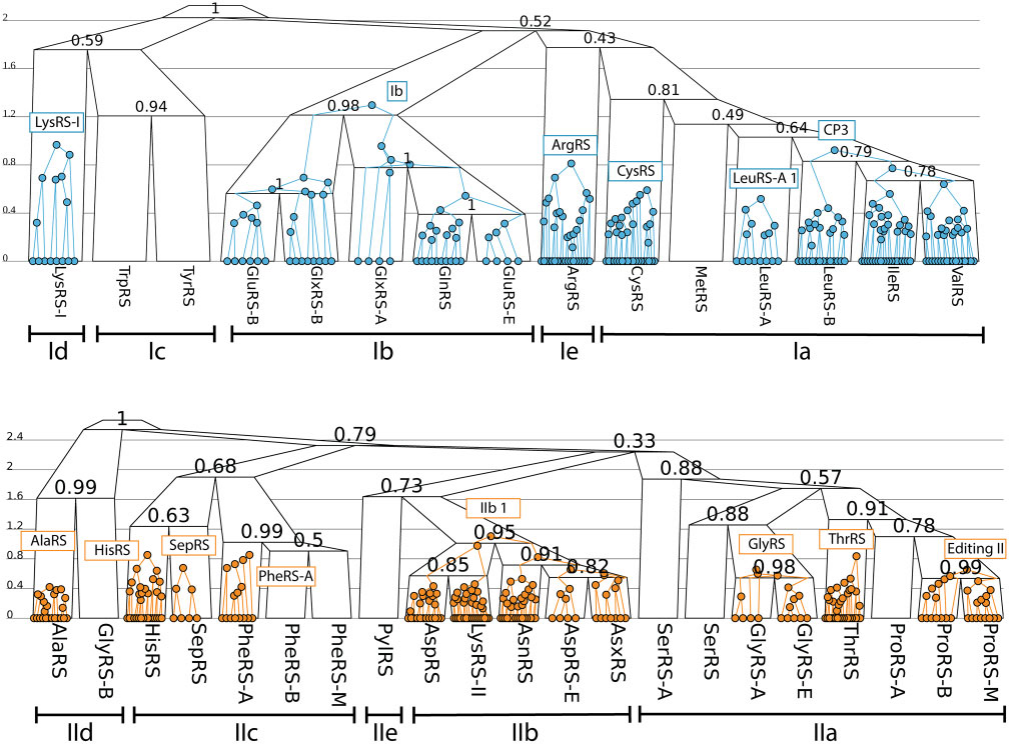
Phylogenies of Class I (top) and II (bottom) catalytic domains. A selection of module trees (coloured) are displayed within the catalytic domain family trees (black). Family tree internal nodes are labelled by clade posterior support. The y-axes depict the rate of change (amino acid substitutions per site and births/deaths per module, weighted according to their instantaneous rates, (see Supporting information), in contrast to the phylogenies in Supplementary Figures S1–S2 which are expressed in substitutions per site, and show similar heights for Class I and II trees. The remaining insertion modules, omitted from this diagram, are shown in Supplementary Figures S42–S67.

**Figure 4.**
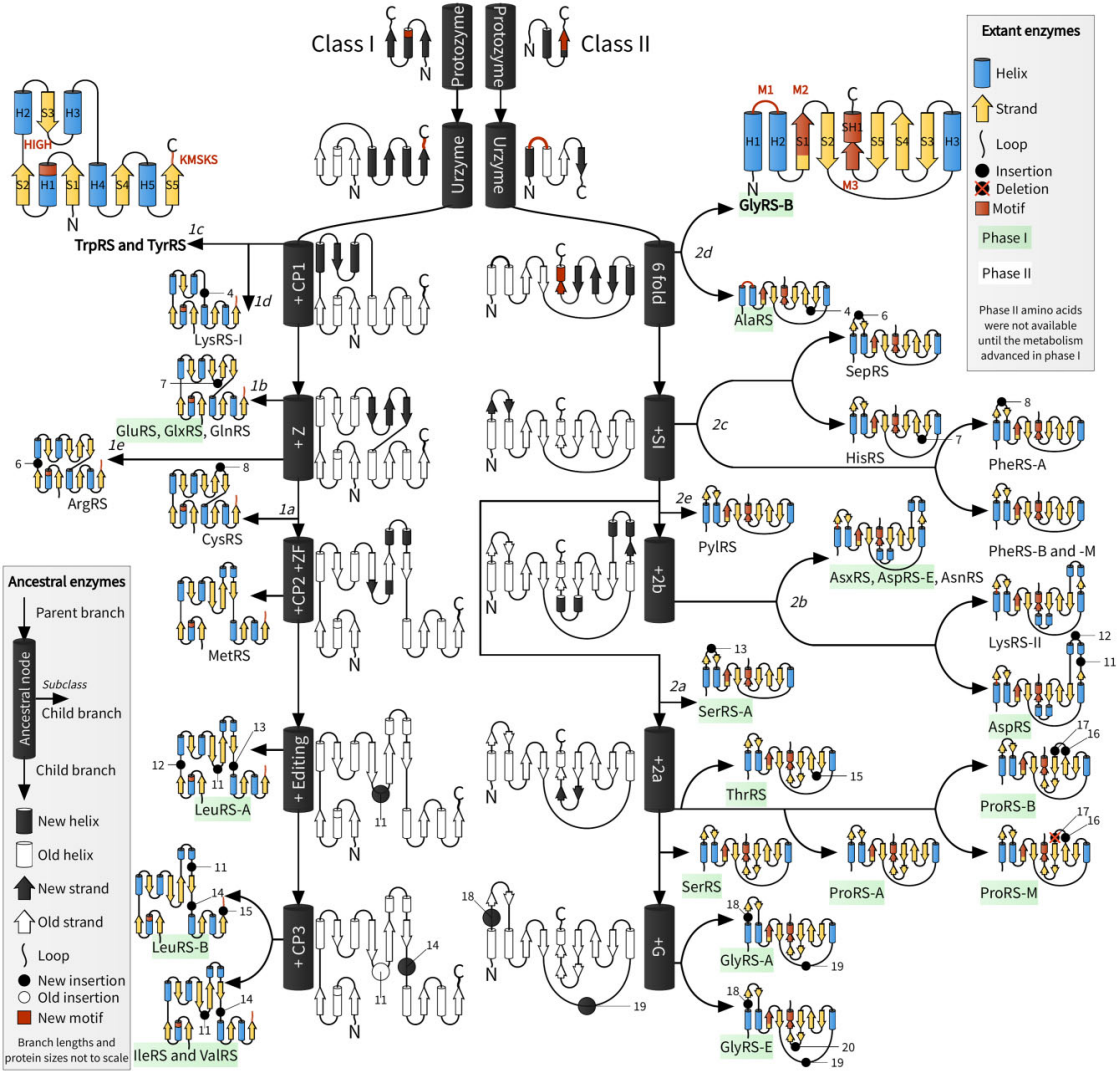
AARS accretion model. Branching off from the central black-and-white ancestral lineages into extant proteins could have occurred at any time, and hence arrows do not denote the passage of time, but rather evolutionary relationships. The temporal component of this figure is depicted by the phase I and II amino acids, as identified by Wong 2005 (6), where we have assigned Pyl and Sep to phase II. Insertion modules are numbered using the key in Table 1. HIGH and KMSKS are the motifs of Class I, and M1–M3 are the Class II motifs 1–3 (68). Loops may contain other secondary structures (see Figure 1).

**Figure 5.**
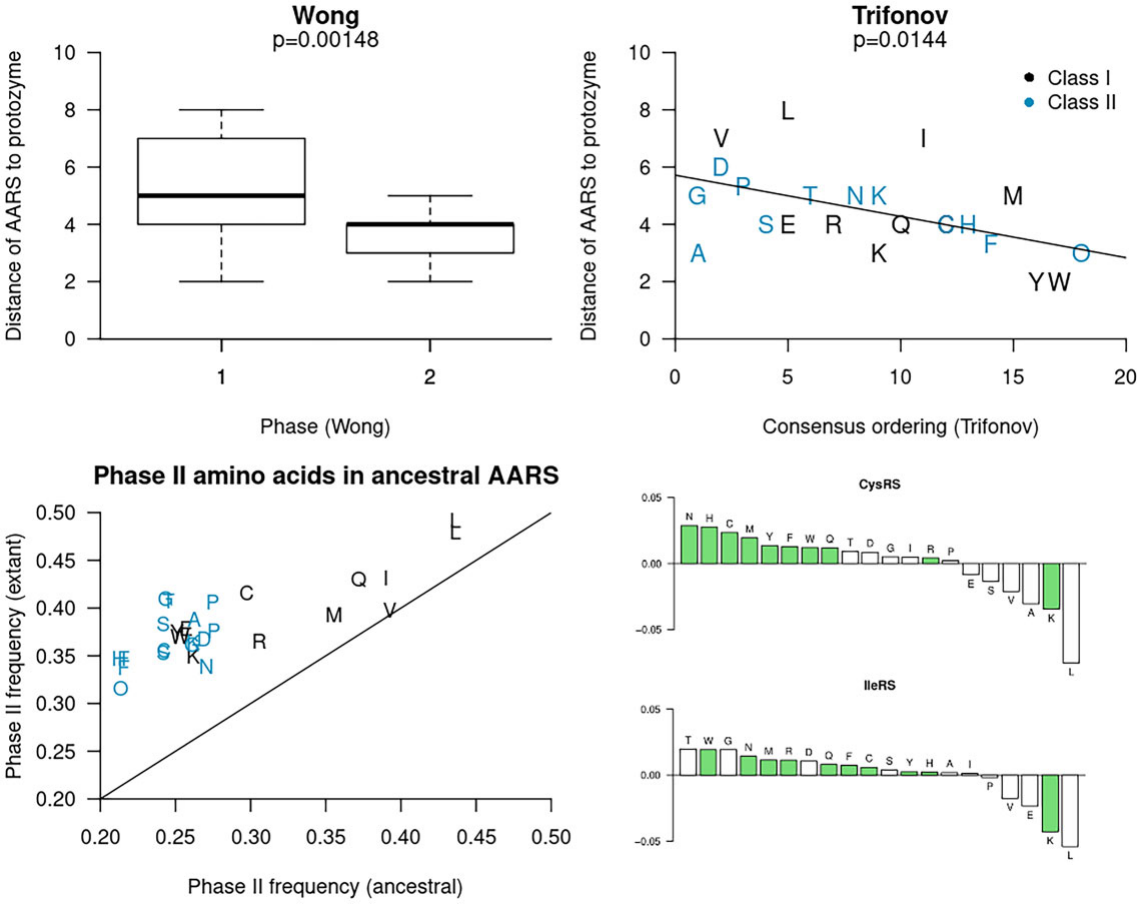
Top: the distance between each extant AARS and the protozyme, according to Figure 4. This distance is defined as the number of IMs that were inserted to assemble the extant AARS from the protozyme. For example, LeuRS-A has a distance of 8. Top left: the *p*-value is the result of a one sided Student *t*-test with a null hypothesis that the phase I and II amino acids (6) are activated by AARS which are equally close to the protozyme (i.e., the same degree of structural primitivity). Top right: the *P*-value is from a two-sided Pearson test between distance to protozyme and Trifonov’s consensus ordering (5). We note the inclusion of pyrrolysine (O), which was absent from Trifonov’s ordering, but has been assigned here as a latecomer due to its metabolic dependency on lysine (108). These two experiments are consistent with the hypothesis of more recently occurring amino acids being recognised by simpler AARS catalytic domains, particularly for Class I. Bottom: the proportions of phase II amino acids were estimate for the most recent common ancestor of each AARS family, and compared with the same estimates from their extant forms. These results show an increase in phase II amino acid use for assembling AARS proteins through time. The increase in amino acid frequencies for two such families (CysRS and IleRS) are further broken down, with phase II amino acids coloured green. Analogous plots for the remaining families are presented in Supplementary Figures S40 and S41.

### Insertion-deletion Dollo model

This Bayesian phylogenetic model has two components. First, IM evolution is modelled as a birth process, followed by either loss (a death event) or retention by extant taxa, following a stochastic Dollo process (52). This approach distinguishes between IMs lost from ancestral proteins and those never present, and assumes that all forms of an IM are homologs of a common ancestor, thus requiring careful identification of IMs. Second, the amino acid sequence evolves down the tree originating at the birth event using established substitution models for protein evolution (48). These module phylogenies are constrained within a family phylogeny, analogous to the multispecies coalescent model (53). All parameters, including trees, IM birth and death rates, and amino acid substitution parameters, are jointly inferred within a Bayesian framework, allowing for hypothesis testing and quantification of Bayesian posterior support.

The posterior density of this model is expressed in Equation 1, where the protein tree *g* is constrained within the protein family tree *S*. The insertion module data is represented in a binary form, where *M*_*i*, *j*_ = 1 if taxon *j* has module *i* = 1, 2, ⋅⋅⋅, *k*, or 0 otherwise. Taxon *j* has amino acid sequence *D*_*i*, *j*_ if and only if *M*_*i*, *j*_ = 1, whose sites are assumed to evolve independently down tree *g* under a continuous time Markov process (54). The stochastic Dollo model (the module likelihood) assumes that all 1’s are homologous and were derived from a common birth event, such that loss of the module is irreversible (52). Each node of the family tree *S* describes a population of modules which belong to the same family, constituting a tree prior distribution governing how module lineages coalesce within each population of families (53) with effective population size **N_e_**, estimated per branch. The estimated model parameters θ include a pure-birth protein tree diversification rate, and a module birth and death rate - which are all relative to the amino acid substitution rate fixed at 1 - as well as vector **N_e_**, and other parameters pertaining to the OBAMA substitution model (48) and family tree relaxed clock (51). θ also includes module birth times *B* = (*b*_1_, *b*_2_, ⋅⋅⋅, *b*_*k*_), which specify the time of the origin of each insertion module. Further details can be found in Supporting Information.


(1) \begin{eqnarray*} \overbrace{ p(S,g,\theta |D,M)}^{\text{Posterior density}} &\propto & \prod _{i=1}^k \Big ( \overbrace{p(D_i|g,\theta )}^{\text{Sequence likelihoods}} \Big ) \times \overbrace{p(M|g,\theta )}^{\text{Module likelihood}} \nonumber\\ &&\times\, \overbrace{p(g|S,\theta )}^{\text{Module tree prior}} \times \overbrace{p(S|\theta )}^{\text{Family tree prior}}\times \overbrace{p(\theta )}^{\text{Other priors}} \nonumber \\ \end{eqnarray*}

## Results

### Families of catalytic domains

Catalytic domain sequences and structures were compared in order to identify AARS families. Although available experimentally solved AARS structures are manifold, they are oftentimes incomplete, harbour solubility-enhancing mutations or truncations, and are far from a representative sample of the biosphere, as they tend to be sourced from organisms that are culturable or have medical or economic significance. To address these biases, we used AlphaFold to generate 422 taxonomically-representative AARS structural models, which were structurally aligned so they could be used for phylogenetic inference. To validate the reliability of these structural models, we compared them with closely related solved structures (Supplementary Figure S3). This experiment confirmed that variation within experimentally solved structures of the same family was similar to the variation between experimental and AlphaFold structures of the same family (*p* > 0.1). Moreover, the pLDDT scores of our AlphaFold structures were large, indicating a high level of confidence, likely reflecting the preponderance of experimentally solved AARS structures used to train AlphaFold. The median pLDDT scores were 96.0% and 96.7% for the Class I and II catalytic domains, and their lower quartiles 93.1% and 93.9%. Low scoring regions (<60%) are invariably confined to short loops on the surface of the protein, often consistent with prior observations of flexibility or disorder, such as the flexible area flanking the KMSKS motif of Class I (55), the flexible small interface loop downstream of motif 1 in Class II (56–58), and the flexible loop found on the surface of CysRS (30). However, disorder does not imply low confidence, for instance the disordered insertion on the surface of the eukaryotic GlyRS (32) has quite high support (over 85%; Supplementary Figure S24). This may indicate conditional disorder, where the module adopts a conformation upon binding (59). For a further breakdown of pLDDT scores, please refer to Supplementary Figures S4–S39. Overall, these results provide confidence that the AlphaFold structures should be informative in comparative analysis.

We identified 36 families of AARS catalytic domains: 15 for Class I and 21 for Class II. Each family meets the following requirements. First, there is a minimum of four samples from four phyla, and where possible, up to eight bacterial phyla, four archaeal phyla, four eukaryotic phyla, and one viral phylum, plus two organellar (mitochondrial or chloroplast) samples from two distinct eukaryotic phyla. Although the emergence of eukaryotes and their organelles occurred relatively late in evolution (60), the inclusion of their AARS can assist in inferring the earlier phylogeny, and allow the identification of further insertion modules. Second, all members of a family are predicted to display common aminoacylation activity based on their similarity to functionally characterised homologs. Third, each family is monophyletic, or monophyletic with a second family contained within it. Finally, in the event of a family containing a clade that can be further distinguished by an insertion or deletion of at least 50 amino acids, it was recursively split into two families, provided that both candidates meet these four requirements. The families are summarised in Supplementary Table S1.

Families are identified with unique short names. In this notation, an AARS that is largely restricted to a certain taxonomy is suffixed accordingly: ‘A’ for archaeal-like, ‘B’ for bacterial-like, ‘E’ for eukaryote-like, and ‘M’ for mitochondrial-like. Most catalytic domain families are unique in their aminoacylation activity, with the following six exceptions.

- The dual forms of LysRS: as anticipated, LysRS belongs to two families LysRS-I and LysRS-II, one for each class (19).
- The dual forms of LeuRS: an archaeal-like form LeuRS-A and a bacterial-like form LeuRS-B, where eukaryotic genomes express either one. The two forms differ in the placement of the editing domain within the catalytic domain (61,62).
- The dual forms of SerRS: the standard SerRS found in most organisms differs from the SerRS-A form found in certain archaea (63).
- The dual forms of AspRS: the standard form AspRS found in bacteria/archaea and the eukaryotic form AspRS-E (64). The latter appears to have diversified from AsxRS post-LUCA.
- The two forms for GluRS: GluRS-B and GluRS-E. These discriminating forms arose convergently from the non-discriminating ancestral GlxRS. The bacterial form is characterised by a helical anticodon binding domain, while GluRS-E has a β-barrel anticodon binding domain (65).
- The two forms for GlxRS: GlxRS-A and GlxRS-B. Like the rest of subclass *Ib*, the bacterial forms are characterised by a helical anticodon binding domain, and GlxRS-A by a β-barrel (65).
- The three forms for ProRS: ProRS-A, ProRS-B and ProRS-M (66), where ProRS-B is characterised by an editing domain within the catalytic domain, which is absent from ProRS-A and most members of ProRS-M.
- The three forms for GlyRS: GlyRS-A, GlyRS-E and GlyRS-B. The first two are dimeric, and the third exists as a heterotetramer. GlyRS-E is differentiated from GlyRS-A by the presence of an ∼90 amino acid insertion.
- The five PheRS families: PheRS-Aα, PheRS-Aβ, PheRS-Bα, PheRS-Bβ and PheRS-M. The PheRS-A and -B forms are heterotetrameric, but only the α chains display catalytic activity (67). As such, the β chains are omitted from our main evolutionary model, but have been included in Supplementary Figure S2.

These 36 families include the same 24 families identified by Perona and Hadd 2012 (33), plus an additional 12. The 24 identified by Perona and Hadd corresponded to the 20 canonical amino acids, plus SepRS and PylRS, but with two families of LysRS, and two families of GlyRS. The full list of accessions and their family assignments can be found in Supplementary Data.

### Phylogeny of insertion modules

We examined protein structures from the 36 families to identify features endemic to each class and the insertion modules found in specific families (Figure 1). An insertion module (IM) is defined as a conserved structural element that is contiguous in sequence, with an average length of at least 30 amino acids in over half the members of a single family, or at least 10 amino acids but with a distinct IM nested within it. These length requirements improve the reliability of inferring homology among IMs, but it does mean that some conserved elements (such as the 1-2 short helices downstream of connecting peptide 1 in TrpRS and TyrRS) were not included in the analysis. Our search was confined to the catalytic domains; we did not consider IMs in editing or anticodon binding domains for instance. If an editing domain was nested within the catalytic domain (as in ProRS and ValRS), we considered the domain as a single IM and did not dissect any IMs within it. Our analysis identified 15 modules for Class I and 20 for Class II (Table 1). The elements common to all members of each class are helices H1-H5 and strands S1–S5 for Class I, and helices H1-H3 and strands S1–S5 for Class II. The final Class II strand is immediately followed by a helix and hence denoted as SH1 (which contains motif 3 (68)). Some of these helices contain a one-residue interruption, such as a turn, and therefore can be regarded as kinked helices, for example H4 in IleRS.

**Table 1.** Summary of modules and their proposed functional roles

Class	Nr	Module	Structure	Functions	Length (aa)
I	1	**Protozyme**	Molten globule	Amino acid activation (72,73)	37−56
	2	**Urzyme**	4 stranded Rossmann fold	tRNA aminoacylation (28)	87−110*
	3	CP1	Exoskeleton †	tRNA binding, dimerisation (76,102)	43−73
	4	LysRS-I	β rich domain (19)		58−90
	5	Z	3 antiparallel β-strands (77)		16−51
	6	ArgRS	3-5 helix bundle (77)		37−113
	7	*Ib*	Loop flanked by two helices	Acceptor stem recognition (27)	63−100
	8	CysRS	Partially disordered lasso	tRNA binding (30)	30−55
	9	CP2	2 helix bundle	Amino acid activation and editing (78)	30−39
	10	ZF	Cysteine-rich zinc finger	tRNA aminoacylation (79,80)	19−44
	11	Editing I	Large globular domain	Post-transfer editing (61,62)	172−318
	12	LeuRS-A 1	2 helices (62)		25−74
	13	LeuRS-A 2	4 helices (62)		52−76
	14	CP3	Cysteine-rich zinc finger (62)		25−77
	15	LeuRS-B	Several β-strands, 2 helices (61)		38−87
II	1	**Protozyme**	Molten globule	Amino acid activation (72,73)	35−50
	2	**Urzyme**	3 stranded antiparallel fold	tRNA aminoacylation (28)	73−88*
	3	**6 fold**	6 stranded antiparallel fold		131−155
	4	AlaRS	Helical loop †	tRNA aminoacylation (83,103)	30−40
	5	SI	Loop	Aminoacylation, dimerisation (56–58)	13−28
	6	SepRS	Disordered / helical bundle (104)		73−77
	7	HisRS	Disordered †	tRNA binding (105)	92−128
	8	PheRS-A	2 helices (67)		28−41
	9	*IIb* 1	Loop flanked by two helices †		43−80
	10	*IIb* 2	2 helix bundle		24−46
	11	AspRS 1	6-stranded antiparallel fold	tRNA interactions (31)	44−66
	12	AspRS 2	*See above*		45−48
	13	SerRS-A	Helix-turn-helix	Dimerisation (63)	36
	14	*IIa*	β-hairpin		6−18
	15	ThrRS	Helix-strand †	Amino acid activation (106)	35−42
	16	ProRS	β-hairpin followed by loop		32−36
	17	Editing II	Large soluble domain	Post-transfer editing (66)	147−173
	18	GlyRS 1	Zinc ribbon	tRNA aminoacylation (32)	44−115
	19	GlyRS 2	2 strands and 2−3 helices (32) †		37−44
	20	GlyRS-E	Disordered	tRNA binding (32)	86−94

Modules in bold font are ancestral catalytic domains, and those in standard font are insertions. Module length ranges are 95% credible intervals across all AlphaFold generated structures. †These elements contain a strand which runs parallel to the N-terminal edge of the Rossmann fold (Class I) or the C-terminal edge of the β-sheet (Class II). *Universal urzyme structures were constructed from aligned helices and strands, excluding loops, so these values underestimate the expected lengths (∼130 aa).

We developed a Bayesian phylogenetic method to integrate IMs with amino acid sequence data (see Methods). This model differs from standard sequence-based phylogenetic methods because it explicitly accounts for modular insertion and deletion. Under our prior distributions, IMs were assumed to appear and disappear at characteristic birth and death rates, which are considerably lower than the rates of amino acid substitution. The estimated birth/death rates were further informed by the data, which is evident when comparing the peaked posterior distributions with the flat and uninformed prior distributions of Figure 2, and Class II was estimated to have a higher birth rate than Class I (consistent with its higher count of IMs). In most cases, when an extant protein was lacking an IM, it was explained as lack-of-birth, as opposed to deletion. But notably, a post-transfer editing domain (Editing II) appears to have been deleted from the mitochondrial ProRS after it diverged from the bacterial-like form (Supplementary Figure S56). This truncated form ProRS-M is phylogenetically distinct from the *Rhodopseudomonas palustris* ProRS, which is also lacking the editing domain (66), however it belongs to the ProRS-B clade (Supplementary Figure S2). Therefore, ProRS-M represents an additional form of ProRS over the three described by Crepin *et al.* (66). We examined eight ProRS-M samples; seven of which are predicted to localise to mitochondria, and have lost the domain, while the last, in *Candida albicans*, is predicted to reside in the cytoplasm, and has retained the domain (or perhaps lost and reacquired it). When the editing domain was lost, it left behind an evolutionary scar, in the form of the small cysteine-rich ProRS IM. ProRS-M is the only AARS family for which there is no experimentally solved structure.

The catalytic domain phylogenies informed by both IM and amino acid data are presented in Figure 3. Accounting for insertion modules gave similar phylogenies to standard phylogenetic approaches (Supplementary Figures S1 and S2), but offering structural context to the interpretation. These analyses support splitting off the LysRS-I, ArgRS, and PylRS families into singleton subclasses *Id*, *Ie*, and *IIe* respectively, due to the absence of close relatives or uncertainty concerning placement in existing subclasses. The results provide a number of insights. First, our placement of HisRS into *IIc*, as opposed to *IIa*, is incongruent with most studies (18,26,33,34). Many of these studies placed HisRS into *IIa* because of its mode of tRNA binding via an anticodon binding domain, which is homologous with members of *IIa*. Here however, we considered the phylogeny of the catalytic domain in isolation from other domains, and thus the anticodon binding domain of HisRS was likely exchanged with that of *IIa*. *Ic* and *IIc* alike are structurally simple, are not characterised by any IMs, and they adenylate some of the larger aromatic amino acids. Second, we placed PylRS into its own subclass *IIe*, which is closely related to *IIb*, congruent with a previous sequence-based analysis (36). However, a previous structural analysis placed it with *IIc* (35). Given that PylRS has the same profile of IMs as *IIc*, the high structural similarity scores with these families are not unexpected. Third, our placement of ArgRS and LysRS-I into singleton subclasses is at odds with some prior studies, many of which consider the mode of tRNA recognition in their classifications (26,33,34). The deep phylogenies describing relationships between subclasses is challenging to resolve, as reflected by the comparatively low levels of posterior support on internal nodes closer to the roots of Figure 3. Our full posterior distributions are summarised in Supplementary Tables S1-S2.

## Discussion

We describe a likely assembly of AARS catalytic domains, layer by layer throughout evolutionary history (Figure 4). This model was generated using a Bayesian phylogenetic method that integrated information from amino acid substitutions with the presence or absence of insertion modules (Figure 3). The phylogenetic method is open-source and is readily available for future use (see Materials and methods). To begin our discussion, we first provide a brief overview of the origins of the Class I and II AARS. We then consider possible processes by which extant catalytic domains were assembled from small structural modules, which grew progressively on the surface of the protein, under principles similar to those described by Petrov et al. (69) for the accretion of RNA onto the ribosome. This process enabled discrimination between closely related amino acid side chains and tRNA molecules. Finally, we discuss the implications of these findings for the interconnected evolution of the genetic code and metabolism.

### Inception of the AARS

One major theory on the origin of the AARS suggests the two AARS classes arose simultaneously as opposing strands of a bidirectional gene (17,70,71). This hypothesis, initially proposed by Rodin and Ohno (70), has prompted a series of experimental investigations into the reconstructed ancestral forms of the two AARS classes. These earliest forms were likely small, low-specificity, molten globules, known as protozymes (71). Although model protozymes from both classes have been experimentally investigated and found to exhibit adenylation activity (72,73), it is not clear how tRNA would have been aminoacylated or how the first protozyme genes originated. In extant proteins, the protozymic region contains the HIGH motif for Class I, and motif 2 for Class II (68). However, it is unlikely that the histidine in the HIGH motif, or the arginine in motif 2, were part of the coding alphabet at this early stage (5,6,74). The Class I protozyme would later be modified by a second crossover, leading to the Rossmann fold, and the Class II protozyme would expand into an antiparallel β-sheet, giving rise to the Class I and II urzymes, which have been shown to aminoacylate tRNA (28). These expansions included the KMSKS motif in Class I and motif 1 Class II, respectively (68). The subsequent steps introduced nested insertions that differentiated the different AARS families and would have necessarily decoupled bidirectional coding into separate Class I and II genes.

The structures resulting from all of these later steps have no bearing on whether the urzymes of the two AARS classes have a common bidirectional origin. Much like the inception of the AARS, most of these later steps most likely happened pre-LUCA, with a few exceptions, including the extensive diversification events within subclasses *Ib* and *IIb* (64,65) and within PheRS (75).

### Class I assembly

The phylogeny of the Class I catalytic domain resembles a ‘caterpillar tree’ with a central lineage providing the trunk from which extant enzymes emerged. This hierarchy of enzymic complexity, the result of gradual modular accretion, is reflected in the nearly linear progression from structurally simpler enzymes (TrpRS and TyrRS) to intermediate (ArgRS and GluRS) to more elaborate ones (ValRS and LeuRS).

Connecting peptide 1 (CP1) occurred early in Class I history, wrapping around the core like an exoskeleton (76). Two lineages diverged from the central Class I AARS lineage: one giving rise to subclass *Ic* (TrpRS and TyrRS), and another giving rise to *Id* (LysRS-I) with an anticodon binding domain similar to GluRS (19,27). However, it is unlikely that there was an abundance of tryptophan, tyrosine, or lysine until much later in evolution of metabolism (6), suggesting that the genesis of *Ic* and *Id* may have occurred much later in time than *Ia* and *Ib*.

The C-terminal of CP1 was later modified by inserting the Z-fold—an antiparallel β-sheet consisting of three strands Z1, Z2 and Z3. ArgRS presents this Z-shaped module in its most primitive form (Ins-2, (77)), which appears unrelated to the β-rich insert found in LysRS-I. This β-sheet provided a platform for future additions nested between its three strands, notably a cysteine-rich zinc finger (ZF) at the end of Z1, and a short two helix bundle (connecting peptide 2, CP2) at the end of Z2. These two modules characterise subclass *Ia* and contribute to aminoacylation (78–80), however the zinc-coordinating cysteine and histidine residues are not entirely conserved, and therefore the ZF region does not always bind zinc (62). The arrival of these two modules coincided with the extension of Z1 and Z2 from around 4 to around 10 amino acids in length, such that it resembled a β-hairpin. A post-transfer editing domain provided the means to discriminate between amino acids with very similar side chains: leucine, isoleucine, and valine. Interestingly, this module occurs in two distinct positions: between CP2 and Z3 for LeuRS-B, and nested within the zinc finger for other enzymes (61,62). It is unclear whether the domain originated in one of these two positions or elsewhere in the proteome. Subclass *Ib* branched off separately from *Ia* and diversified post-LUCA into GlnRS and various discriminating and non-discriminating forms of GluRS (65). GluRS, GlnRS, and GlxRS have similar catalytic domain structures, however the bacterial and archaeal/eukaryotic lineages differ in their anticodon binding domains (65).

### Class II assembly

The phylogeny of the Class II catalytic domain is much more balanced, or ‘tree-like’, than that of Class I (Figure 4). This can perhaps be attributed to the structural plasticity of its antiparallel β-sheet fold, which, much like the smaller antiparallel sheet Z of Class I, provided fertile ground for the rapid proliferation of insertion modules within the loops connecting consecutive strands. Many of these insertions were stabilised by the formation of an additional strand running parallel to the sheet’s C-terminal edge (Table 1). Taken together, it appears that the Class II fold is more receptive to insertions than the Class I Rossmann fold.

Early in the history of Class II, a short loop, known as the small interface (SI) (57), emerged on the surface of the protein. The N-terminal region of SI works intimately with the active site through a range of distinct mechanisms, sequence signatures, and structures, and has been termed the flipping loop (56), the ordering loop (81), and the helical loop (82). Together with a strand in motif 1, the C-terminal region of SI appears at the dimeric interface where it often forms a six-stranded antiparallel sheet across the two subunits (C2–C3 loop, (58)). This β-hairpin would later acquire nested insertions on three independent occasions: PheRS-A, SepRS, and SerRS-A. SI emerged only after the divergence of *IId*, whose members oligomerise through mechanisms quite distinct from the rest of the class, a coiled coil for AlaRS (83) and a three-helix bundle for the tetrameric GlyRS-B (84).

### An unexpected inversion

Elaboration of the successive insertion modules defining the AARS families has revealed a curious inversion. AARS for the simplest amino acids have, in general, accumulated more insertion modules. Examining Figure 4, we observe that the catalytic domains of AARS that bind to phase II amino acids (as defined by Wong (6): see below), which supposedly appeared later in the coding alphabet, have, on average, significantly fewer insertion modules than those for phase I. This inversion is most clearly illustrated in tryptophan and tyrosine, which may have been the last two amino acids to enter the coding alphabet (5), and yet their AARS did *not* diverge from those of the earlier canonical amino acids, such as valine or glutamate, as one might expect. Rather, the genesis of TrpRS and TyrRS is rooted deep within the Class I phylogeny (Figure 3) and their catalytic domains are similar to the earliest ancestral structures (Figure 4).

Two interrelated observations help explain the unexpected strength of this inversion. First, as Pauling (85) noted, simpler amino acid side chains are harder to select without error. Rejecting small, similarly-shaped side chains required the acquisition of insertions to modulate the basic specificity determinants and eventually facilitate editing of incorrectly activated or misacylated amino acids. More complex side chains increase the scale of differences, facilitating discrimination with fewer structural tweaks.

Second, Wong’s coevolutionary model for genetic code expansion suggests a complementary inference. Wong (6) distinguished those amino acids produced in abundance through prebiotic chemistry or simple metabolic pathways as phase I amino acids. He proposed that these served as metabolic precursors for more complex phase II amino acids that required more extensive biosynthetic pathways. Wong arrived at a similar delineation to previous inferences based on different methods, including Trifonov’s consensus approach (5) and Brooks’ phylogenetic approach (86). The earliest proteins were presumably synthesised from a limited pool of phase I amino acids using promiscuous AARS and an ambiguous genetic code. With time, the binding specificities of AARS sharpened by acquiring new modules, allowing them to sterically discriminate between closely related amino acid types. This then enriched the types of molecules available through more elaborate metabolic pathways, eventually producing the amino acids of phase II. These, in turn, became particularly valuable for catalysis (notably the side chains of histidine, arginine, lysine, cysteine, and tyrosine (87)). This reasoning recently gained experimental support from a demonstration that the histidine and lysine side chains in the Class I sequence motifs contributed little to catalysis, and were in fact inhibitory, in an ancestral model of the LeuRS-A urzyme which lacked CP1 (74).

This trend exists across Wong’s and Trifonov’s amino acid orderings (*P* < 0.01 and *P* < 0.02, respectively; Figure 5). Moreoever, by reconstructing the ancestral sequences of each AARS family, we showed that the proportion of phase II amino acids increased through time for each family (Figure 5), consistent with the experiments performed by Brooks *et al.*, which were on different ancient proteins (86). Taken together, these results are consistent with this inversion being a common trend, but not universal across all AARS (notable exceptions include GlyRS-B and AlaRS).

These results highlight the far-reaching question of how ancestral AARS protein folding evolved concurrently with the expansion of the coding table (88). Although the earlier coding alphabets must have been sufficient to enable protein folding, it remains unclear how similar ancestral AARS folds were to those we infer from structures of their present-day descendants. We can, however, cite evidence for likely characteristics of those early folds. Urzymes lacking the insertion modules described here show uniformly high catalytic rate accelerations. Their structural repertoires must therefore include active-site configurations complementary to the transition states for amino acid activation and RNA aminoacylation. Preliminary nuclear magnetic resonance evidence for TrpRS (89) and LeuRS (90) urzymes imply substantially broader structural variances than those of properly folded proteins. Both are thus probably catalytically active molten globules. Enzyme fragments homologous to Class I AARS protozymes exhibit ligand-dependent folding transitions (91–93). Early AARS ancestors thus probably resembled contemporary forms, albeit transiently, as complexes with amino acid and RNA substrates.

### Expansion of the primordial genetic code through retrofunctionalisation

Suppose that a novel amino acid type, *X*, were to emerge in abundance from a new metabolic pathway. A number of scenarios could follow, each exerting unique selective pressures on the protocell and the metabolic pathways that produce the amino acid. In the event that *X* were not recognised by existing AARS to any significant extent, its production would have no material impact on the genetic code. In a second scenario, were *X* to be recognised by existing AARS in a way that interfered with the protein synthetic machinery by perturbing its products, the production of *X* would be selected against, or perhaps there would be selection for AARS to preclude *X*. For instance, meta-tyrosine is a toxic amino acid which competes with phenylalanine during protein synthesis, leading to defective proteins, but PheRS catalyses the removal of mistargeted meta-tyrosine through its editing activity (75). The emergence of amino acid types that react with tRNA, such as glutamine and homocysteine, may also impose a selective disadvantage for the diversification of their cognate AARS (9). In the third case, a midpoint between these two extremes, suppose *X* were to be recognised by AARS in a non-disruptive manner, allowing it to gradually work its way into the genetic code. By establishing itself as an essential metabolite, *X* and the metabolic pathways for its production would be selected.

The least disruptive way to incorporate *X* into the genetic code would be through its recognition by a promiscuous, and perhaps low-activity, AARS, as opposed to one of the more specialised enzymes, which would have evolved more precise substrate recognition and enabled, for example, discrimination between leucine and isoleucine, or serine and threonine. Thus, the most fruitful place to find such an AARS would be among the ancient lineages, perhaps acquired by exchanging genetic material with a geographically isolated population at a different stage of evolution. From there, the specificity of *X*-tRNA synthetase could be refined by using the newly available phase II amino acids, and their advanced catalytic propensities (87). This proposed mechanism is a variation on the epistatic ratchet observed in the evolution of specificity in steroid hormone receptors (94).

Placement of *X* into the genetic code would be determined by the anticodons of whatever tRNA molecules were recognised by the adapted *X*-tRNA synthetase. As demonstrated by the dynamic phylogeny of tRNA specificity (95), and the sheer number of AARS modules (Table 1) and domain superfamilies (26) involved in tRNA recognition, the interaction between tRNA and AARS has been fairly malleable. Thus, the fluid nature of the pairing between amino acids and anticodons would enable *X* to assume a place in the genetic code, while also optimising the code’s robustness under the error minimisation principle (2).

As the code evolved, amino acid types competed for a place in the parliament of 64 seats. There are several routes which amino acid types have taken to enter the genetic code. First, there is subfunctionalisation (96), whereby a promiscuous AARS duplicates, and its daughters adapt to discriminate between the amino acids recognised by the parent. This mechanism has been considered for the ancestor of IleRS and ValRS (97). Second, through neofunctionalisation, a duplicate of an existing specialised AARS is co-opted to supply a new amino acid, and has been suggested for the ancestor of TrpRS and TyrRS (98). Third, pretranslational modification enabled unstable amino acids (asparagine, glutamine, and selenocysteine) to enter the coding alphabet without the need for an AARS duplication event (6,23,26). Lastly, as demonstrated here, the recruitment of ancient, unspecialised AARS lineages provided a fourth route. However, much like the third route, this process does not readily fit into the framework of specificity-refinement or functional gain among gene duplicates, but rather it is a change in environmental condition (i.e., substrate availability) that enables an unfulfilled capacity (i.e. recognition of that substrate), dormant within the broader pool of AARS genes, to manifest as a novel biological function much later in time. In contrast to neofunctionalisation, the new function would emerge from a change in environment rather than a change in sequence, and in contrast to subfunctionalisation, the drive for specialisation would not exist until its function was activated. This process of *retrofunctionalisation* may have been the point of entry for tryptophan, tyrosine, arginine, histidine, phenylalanine, pyrrolysine, cysteine and methionine, all of which most likely entered the genetic code quite late, and yet their cognate AARS often have comparatively primitive catalytic domains. While retrofunctionalisation may be a common trend, especially in Class I, it is not universal - for example GlyRS-B and AlaRS have primitive catalytic domains but they also recognise simple amino acids. Further consideration of the mode of operation and detailed effects of this mechanism may help resolve the order in which amino acids entered the code, irrespective of which AARS class supplies them for rendering into proteins, and may also prove useful in attempts to expand the repertoire of the code.

### Limitations and assumptions

These methods and results have limitations. First, the structures generated by AlphaFold (38) are predictions, and are no match for experimentally determined structures (99). Although the reliability of these predictions benefits from an abundance of close relatives in the protein databank, they may also induce reference biases that obscure true deviations between structures. AlphaFold structures were not interpreted at an atomic resolution, but were used for more coarse-grained purposes: (a) generating sequence alignments and (b) identifying the presence or absence of insertion models. Moreover, all of the insertion modules described had already been identified in experimental structures. Therefore, the downstream effects of any small inaccuracies made by AlphaFold should be relatively minor. Second, our evolutionary model assumes that the AARS started as small structures that grew in complexity through time. Insertions are therefore assumed to be more frequently occurring than deletions and both events are assumed to be significantly less common than amino acid substitutions, as reflected in our prior distributions. Third, our studies were restricted to the AARS catalytic domain, which has a distinct phylogenetic history from the various editing and anticodon binding domain superfamilies. Fourth, phylogenetic analyses were conducted under the standard assumption made by amino acid substitution models that the amino acid alphabet remained fixed through time, which is most certainly false. This assumption is likely to introduce biases in many places, such as when inferring ancestral amino acid frequencies as we did in Figure 5. This fundamental limitation is prevalent in all phylogenetic approaches to studying ancient proteins that predate the modern coding alphabet, as previously discussed (100,101).

Our proposal of retrofunctionalisation recognises that only a limited subset of the 20 canonical amino acids were likely initially available, e.g., those specified by Wong (6) or Trifonov (5). If all 20 were abundantly available from the onset, then retrofunctionalisation would not be necessary to explain the observed phylogeny. Lastly, as is the nature of all historical recounts, our models can only be as reliable as the breadcrumbs of evidence that have survived the passage of time. The discovery of a novel organism or gene, for instance, could necessitate a revision of the model. Notwithstanding these caveats, we believe our results are robust and provide a useful framework for studying aminoacyl-tRNA synthetases and the genetic code.

## Conclusion

Many efforts to root the origin of the genetic code in a hypothetical RNA world downplay the role of the AARS, the enzymes exclusively known to have operated the code in all known forms of life. AARS phylogeny suggests that the chemical logic of the code was shaped simultaneously by an evolutionary pressure to refine AARS specificities, that is, the ability to discriminate between amino acids with similar side chains, and a pressure to expand the coding alphabet by recognising amino acids produced through emergent biosynthetic pathways. Unexpectedly, the complexity of an amino acid side chain is inversely related to that of its enzyme’s modular structure (Figure 4, Supplementary Figure S4). This inversion suggests that nature crafted specific enzymes for new, more specialised amino acids from the reservoir of relatively non-specific ancestral AARS, which served as blank canvases for expanding the coding alphabet. Following adaptation to the introduction of a new amino acid, the entrenchment of orthogonality - exclusivity in AARS-tRNA pair recognition - gives the code an appearance of it being a ‘frozen accident’ (13). Widely known regularities in the coding table on which the error minimisation theory is founded (2) seem to have arisen from the coevolution of the coding table with the concurrent elaboration of metabolic pathways for more specialised amino acid side chains, as advocated by Wong (6). Increasingly precise genetic coding can only have coevolved with enhanced control over biochemical pathways. The process of retrofunctionalisation is distinct from the three previously observed mechanisms by which AARS lineages would differentiate: subfunctionalisation, neofunctionalisation, and pretranslational modification. Recognising the role of retrofunctionalisation will be especially important in future efforts to characterise ancestral Class I and II aminoacyl-tRNA synthetases.

## Supplementary Material

gkad1160_supplemental_file

## Data Availability

AARS GenBank accessions, family assignments, multiple sequence alignments, and BEAST 2 XML files are available as supplementary data on FigShare at https://doi.org/10.17608/k6.auckland.24406057.v2.

## References

[1] Kondratyeva L.G. , Dyachkova M.S., Galchenko A.V. The origin of genetic code and translation in the framework of current concepts on the origin of life. Biochemistry (Moscow). 2022; 87:150–169. 35508902 10.1134/S0006297922020079

[2] Koonin E.V. , Novozhilov A.S. Origin and evolution of the genetic code: the universal enigma. IUBMB life. 2009; 61:99–111. 19117371 10.1002/iub.146 PMC3293468

[3] Carter C.W. Jr , Wills P.R. The roots of genetic coding in aminoacyl-tRNA synthetase duality. Annu. Rev. Biochem. 2021; 90:349–373. 33781075 10.1146/annurev-biochem-071620-021218

[4] Janzen E. , Blanco C., Peng H., Kenchel J., Chen I.A. Promiscuous ribozymes and their proposed role in prebiotic evolution. Chem. Rev. 2020; 120:4879–4897. 32011135 10.1021/acs.chemrev.9b00620 PMC7291351

[5] Trifonov E.N. Consensus temporal order of amino acids and evolution of the triplet code. Gene. 2000; 261:139–151. 11164045 10.1016/s0378-1119(00)00476-5

[6] Wong J. T.-F. Coevolution theory of the genetic code at age thirty. BioEssays. 2005; 27:416–425. 15770677 10.1002/bies.20208

[7] Takénaka A. , Moras D. Correlation between equi-partition of aminoacyl-tRNA synthetases and amino-acid biosynthesis pathways. Nucleic Acids Res. 2020; 48:3277–3285. 31965182 10.1093/nar/gkaa013 PMC7102985

[8] Harrison S.A. , Palmeira R.N., Halpern A., Lane N. A biophysical basis for the emergence of the genetic code in protocells. Biochim. Biophys. Acta (BBA) Bioenerget. 2022; 1863:148597. 10.1016/j.bbabio.2022.148597 35868450

[9] Hendrickson T.L. , Wood W.N., Rathnayake U.M. Did amino acid side chain reactivity dictate the composition and timing of Aminoacyl-tRNA synthetase evolution?. Genes. 2021; 12:409. 33809136 10.3390/genes12030409 PMC8001834

[10] Brack A. Liquid water and the origin of life. Origins Life Evol. B. 1993; 23:3–10. 10.1007/BF01581985 11536525

[11] do Nascimento Vieira A. , Kleinermanns K., Martin W.F., Preiner M. The ambivalent role of water at the origins of life. FEBS Lett. 2020; 594:2717–2733. 32416624 10.1002/1873-3468.13815

[12] Ribas de Pouplana L. , Torres A.G., Rafels-Ybern À. What froze the genetic code?. Life. 2017; 7:14. 28379164 10.3390/life7020014 PMC5492136

[13] Crick F.H. The origin of the genetic code. J. Mol. Biol. 1968; 38:367–379. 4887876 10.1016/0022-2836(68)90392-6

[14] Illangasekare M. , Sanchez G., Nickles T., Yarus M. Aminoacyl-RNA synthesis catalyzed by an RNA. Science. 1995; 267:643–647. 7530860 10.1126/science.7530860

[15] Lohse P.A. , Szostak J.W. Ribozyme-catalysed amino-acid transfer reactions. Nature. 1996; 381:442–444. 8632803 10.1038/381442a0

[16] Suga H. , Hayashi G., Terasaka N. The RNA origin of transfer RNA aminoacylation and beyond. Phil. T. Roy. Soc. B: Biol. Sci. 2011; 366:2959–2964. 10.1098/rstb.2011.0137 PMC3158918 21930588

[17] Kauffman S. , Lehman N. Mixed anhydrides at the intersection between peptide and RNA autocatalytic sets: evolution of biological coding. J. R. Soc. Interface. 2023; 13:20230009. 10.1098/rsfs.2023.0009 PMC10198252 37213924

[18] Gomez M. A.R. , Ibba M. Aminoacyl-tRNA synthetases. Rna. 2020; 26:910–936. 32303649 10.1261/rna.071720.119 PMC7373986

[19] Terada T. , Nureki O., Ishitani R., Ambrogelly A., Ibba M., Söll D., Yokoyama S. Functional convergence of two lysyl-tRNA synthetases with unrelated topologies. Nat. Struct. Biol. 2002; 9:257–262. 11887185 10.1038/nsb777

[20] Sauerwald A. , Zhu W., Major T.A., Roy H., Palioura S., Jahn D., Whitman W.B., Yates J.R. 3rd, Ibba M., Soll D. RNA-dependent cysteine biosynthesis in archaea. Science. 2005; 307:1969–1972. 15790858 10.1126/science.1108329

[21] Lapointe J. , Duplain L., Proulx M. A single glutamyl-tRNA synthetase aminoacylates tRNAGlu and tRNAGln in Bacillus subtilis and efficiently misacylates Escherichia coli tRNAGln1 in vitro. J. Bacteriol. 1986; 165:88–93. 3079749 10.1128/jb.165.1.88-93.1986 PMC214374

[22] Raczniak G. , Becker H.D., Min B., Soll D. A single amidotransferase forms asparaginyl-tRNA and glutaminyl-tRNA in Chlamydia trachomatis. J. Biol. Chem. 2001; 276:45862–45867. 11585842 10.1074/jbc.M109494200

[23] Lee B.J. , Worland P.J., Davis J.N., Stadtman T.C., Hatfield D.L. Identification of a selenocysteyl-tRNASer in mammalian cells that recognizes the nonsense codon, UGA. J. Biol. Chem. 1989; 264:9724–9727. 2498338

[24] Salazar J.C. , Ahel I., Orellana O., Tumbula-Hansen D., Krieger R., Daniels L., Söll D. Coevolution of an aminoacyl-tRNA synthetase with its tRNA substrates. Proc. Natl. Acad. Sci. U.S.A. 2003; 100:13863–13868. 14615592 10.1073/pnas.1936123100 PMC283512

[25] Skouloubris S. , de Pouplana L.R., De Reuse H., Hendrickson T.L. A noncognate aminoacyl-tRNA synthetase that may resolve a missing link in protein evolution. Proc. Natl. Acad. Sci. U.S.A. 2003; 100:11297–11302. 13679580 10.1073/pnas.1932482100 PMC208751

[26] O’Donoghue P. , Luthey-Schulten Z. On the evolution of structure in aminoacyl-tRNA synthetases. Microbiol. Mol. Biol. Rev. 2003; 67:550–573. 14665676 10.1128/MMBR.67.4.550-573.2003 PMC309052

[27] Nureki O. , O’Donoghue P., Watanabe N., Ohmori A., Oshikane H., Araiso Y., Sheppard K., Söll D., Ishitani R. Structure of an archaeal non-discriminating glutamyl-tRNA synthetase: a missing link in the evolution of Gln-tRNAGln formation. Nucleic Acids Res. 2010; 38:7286–7297. 20601684 10.1093/nar/gkq605 PMC2978374

[28] Li L. , Francklyn C., Carter C.W. Aminoacylating urzymes challenge the RNA world hypothesis. J. Biol. Chem. 2013; 288:26856–26863. 23867455 10.1074/jbc.M113.496125 PMC3772232

[29] Carter C.W. Jr , Wills P.R. Hierarchical groove discrimination by Class I and II aminoacyl-tRNA synthetases reveals a palimpsest of the operational RNA code in the tRNA acceptor-stem bases. Nucleic Acids Res. 2018; 46:9667–9683. 30016476 10.1093/nar/gky600 PMC6182185

[30] Newberry K.J. , Hou Y.-M., Perona J.J. Structural origins of amino acid selection without editing by cysteinyl-tRNA synthetase. EMBO J. 2002; 21:2778–2787. 12032090 10.1093/emboj/21.11.2778 PMC126036

[31] Eiler S. , Dock-Bregeon A.-C., Moulinier L., Thierry J.-C., Moras D. Synthesis of aspartyl-tRNAAsp in Escherichia coli’a snapshot of the second step. EMBO J. 1999; 18:6532–6541. 10562565 10.1093/emboj/18.22.6532 PMC1171716

[32] Qin X. , Hao Z., Tian Q., Zhang Z., Zhou C., Xie W. Cocrystal structures of glycyl-tRNA synthetase in complex with tRNA suggest multiple conformational states in glycylation. J. Biol. Chem. 2014; 289:20359–20369. 24898252 10.1074/jbc.M114.557249 PMC4106348

[33] Perona J.J. , Hadd A. Structural diversity and protein engineering of the aminoacyl-tRNA synthetases. Biochemistry. 2012; 51:8705–8729. 23075299 10.1021/bi301180x

[34] de Pouplana L.R. , Schimmel P. Aminoacyl-tRNA synthetases: potential markers of genetic code development. Trends Biochem. Sci. 2001; 26:591–596. 11590011 10.1016/s0968-0004(01)01932-6

[35] Kavran J.M. , Gundllapalli S., O’Donoghue P., Englert M., Söll D., Steitz T.A. Structure of pyrrolysyl-tRNA synthetase, an archaeal enzyme for genetic code innovation. Proc. Natl. Acad. Sci. 2007; 104:11268–11273. 17592110 10.1073/pnas.0704769104 PMC2040888

[36] Fournier G.P. , Huang J., Gogarten J.P. Horizontal gene transfer from extinct and extant lineages: biological innovation and the coral of life. Phil. T. Roy. Soc. B: Biol. Sci. 2009; 364:2229–2239. 10.1098/rstb.2009.0033 PMC2873001 19571243

[37] Winter D.J. rentrez: an R package for the NCBI eUtils API. 2017; Technical report, PeerJ, Preprints.

[38] Jumper J. , Evans R., Pritzel A., Green T., Figurnov M., Ronneberger O., Tunyasuvunakool K., Bates R., Žídek A., Potapenko A. et al. Highly accurate protein structure prediction with AlphaFold. Nature. 2021; 596:583–589. 34265844 10.1038/s41586-021-03819-2 PMC8371605

[39] Kabsch W. , Sander C. Dictionary of protein secondary structure: pattern recognition of hydrogen-bonded and geometrical features. Biopolymers Origin. Res. Biomol. 1983; 22:2577–2637. 10.1002/bip.360221211 6667333

[40] Wang S. , Ma J., Peng J., Xu J. Protein structure alignment beyond spatial proximity. Sci. Rep. 2013; 3:1448. 23486213 10.1038/srep01448 PMC3596798

[41] Wang S. , Peng J., Xu J. Alignment of distantly related protein structures: algorithm, bound and implications to homology modeling. Bioinformatics. 2011; 27:2537–2545. 21791532 10.1093/bioinformatics/btr432 PMC3167051

[42] Thompson J.D. , Gibson T.J., Higgins D.G. Multiple sequence alignment using ClustalW and ClustalX. Curr. protoc. Bioinform. 2003; Chapter 2:Unit 2.3. 10.1002/0471250953.bi0203s00 18792934

[43] Bouckaert R. , Vaughan T.G., Barido-Sottani J., Duchêne S., Fourment M., Gavryushkina A., Heled J., Jones G., Kühnert D., De Maio N., et al. BEAST 2.5: an advanced software platform for Bayesian evolutionary analysis. PLoS Comput. Biol. 2019; 15:e1006650. 30958812 10.1371/journal.pcbi.1006650 PMC6472827

[44] Rambaut A. , Drummond A.J., Xie D., Baele G., Suchard M.A. Posterior summarization in Bayesian phylogenetics using Tracer 1.7. Syst. Biol. 2018; 67:901. 29718447 10.1093/sysbio/syy032 PMC6101584

[45] Heled J. , Bouckaert R.R. Looking for trees in the forest: summary tree from posterior samples. BMC Evol. Biol. 2013; 13:221. 24093883 10.1186/1471-2148-13-221 PMC3853548

[46] Douglas J. UglyTrees: a browser-based multispecies coalescent tree visualizer. Bioinformatics. 2021; 37:268–269. 32717041 10.1093/bioinformatics/btaa679 PMC8055222

[47] Douglas J. , Zhang R., Bouckaert R. Adaptive dating and fast proposals: revisiting the phylogenetic relaxed clock model. PLoS Comput. Biol. 2021; 17:e1008322. 33529184 10.1371/journal.pcbi.1008322 PMC7880504

[48] Bouckaert R.R. OBAMA: OBAMA for Bayesian amino-acid model averaging. PeerJ. 2020; 8:e9460. 32832259 10.7717/peerj.9460 PMC7413081

[49] Bouckaert R.R. An efficient coalescent epoch model for Bayesian phylogenetic inference. Syst. Biol. 2022; 71:1549–1560. 35212733 10.1093/sysbio/syac015 PMC9773037

[50] Heled J. , Drummond A.J. Calibrated tree priors for relaxed phylogenetics and divergence time estimation. Syst. Biol. 2012; 61:138–149. 21856631 10.1093/sysbio/syr087 PMC3243734

[51] Douglas J. , Jiménez-Silva C.L., Bouckaert R. StarBeast3: adaptive parallelized bayesian inference under the multispecies coalescent. Syst. Biol. 2022; 71:901–916. 35176772 10.1093/sysbio/syac010 PMC9248896

[52] Nicholls G.K. , Gray R.D. Dated ancestral trees from binary trait data and their application to the diversification of languages. J. R. Stat. Soc.: Ser. B (Stat. Method.). 2008; 70:545–566.

[53] Heled J. , Drummond A.J. Bayesian inference of species trees from multilocus data. Mol. Biol. Evol. 2010; 27:570–580. 19906793 10.1093/molbev/msp274 PMC2822290

[54] Felsenstein J. Evolutionary trees from DNA sequences: a maximum likelihood approach. J. Mol. Evol. 1981; 17:368–376. 7288891 10.1007/BF01734359

[55] Kobayashi T. , Takimura T., Sekine R., Vincent K., Kamata K., Sakamoto K., Nishimura S., Yokoyama S. Structural snapshots of the KMSKS loop rearrangement for amino acid activation by bacterial tyrosyl-tRNA synthetase. J. Mol. Biol. 2005; 346:105–117. 15663931 10.1016/j.jmb.2004.11.034

[56] Schmitt E. , Moulinier L., Fujiwara S., Imanaka T., Thierry J.-C., Moras D. Crystal structure of aspartyl-tRNA synthetase from Pyrococcus kodakaraensis KOD: archaeon specificity and catalytic mechanism of adenylate formation. EMBO J. 1998; 17:5227–5237. 9724658 10.1093/emboj/17.17.5227 PMC1170850

[57] Qiu X. , Janson C.A., Blackburn M.N., Chhohan I.K., Hibbs M., Abdel-Meguid S.S. Cooperative structural dynamics and a novel fidelity mechanism in histidyl-tRNA synthetases. Biochemistry. 1999; 38:12296–12304. 10493797 10.1021/bi990482v

[58] Hughes S.J. , Tanner J.A., Hindley A.D., Miller A.D., Gould I.R. Functional asymmetry in the lysyl-tRNA synthetase explored by molecular dynamics, free energy calculations and experiment. BMC Struct. Biol. 2003; 3:5. 12787471 10.1186/1472-6807-3-5 PMC165585

[59] Yegambaram K. , Bulloch E.M., Kingston R.L. Protein domain definition should allow for conditional disorder. Protein Science. 2013; 22:1502–1518. 23963781 10.1002/pro.2336 PMC3831666

[60] Canfield D.E. , Teske A. Late Proterozoic rise in atmospheric oxygen concentration inferred from phylogenetic and sulphur-isotope studies. Nature. 1996; 382:127–132. 11536736 10.1038/382127a0

[61] Cusack S. , Yaremchuk A., Tukalo M. The 2 Å crystal structure of leucyl-tRNA synthetase and its complex with a leucyl-adenylate analogue. EMBO J. 2000; 19:2351–2361. 10811626 10.1093/emboj/19.10.2351 PMC384370

[62] Fukunaga R. , Yokoyama S. Crystal structure of leucyl-tRNA synthetase from the archaeon Pyrococcus horikoshii reveals a novel editing domain orientation. J. Mol. Biol. 2005; 346:57–71. 15663927 10.1016/j.jmb.2004.11.060

[63] Bilokapic S. , Maier T., Ahel D., Gruic-Sovulj I., Söll D., Weygand-Durasevic I., Ban N. Structure of the unusual seryl-tRNA synthetase reveals a distinct zinc-dependent mode of substrate recognition. EMBO J. 2006; 25:2498–2509. 16675947 10.1038/sj.emboj.7601129 PMC1478180

[64] Kern D. , Roy H., Becker H.D. Asparaginyl-tRNA synthetases. Madame Curie Bioscience Database. 2013; Landes Bioscience.

[65] Hadd A. , Perona J.J. Coevolution of specificity determinants in eukaryotic glutamyl-and glutaminyl-tRNA synthetases. J. Mol. Biol. 2014; 426:3619–3633. 25149203 10.1016/j.jmb.2014.08.006

[66] Crepin T. , Yaremchuk A., Tukalo M., Cusack S. Structures of two bacterial prolyl-tRNA synthetases with and without a cis-editing domain. Structure. 2006; 14:1511–1525. 17027500 10.1016/j.str.2006.08.007

[67] Finarov I. , Moor N., Kessler N., Klipcan L., Safro M.G. Structure of human cytosolic phenylalanyl-tRNA synthetase: evidence for kingdom-specific design of the active sites and tRNA binding patterns. Structure. 2010; 18:343–353. 20223217 10.1016/j.str.2010.01.002

[68] Eriani G. , Delarue M., Poch O., Gangloff J., Moras D. Partition of tRNA synthetases into two classes based on mutually exclusive sets of sequence motifs. Nature. 1990; 347:203–206. 2203971 10.1038/347203a0

[69] Petrov A.S. , Gulen B., Norris A.M., Kovacs N.A., Bernier C.R., Lanier K.A., Fox G.E., Harvey S.C., Wartell R.M., Hud N.V. et al. History of the ribosome and the origin of translation. Proc. Natl. Acad. Sci. U.S.A. 2015; 112:15396–15401. 26621738 10.1073/pnas.1509761112 PMC4687566

[70] Rodin S.N. , Ohno S. Two types of aminoacyl-tRNA synthetases could be originally encoded by complementary strands of the same nucleic acid. Origins Life Evol. B. 1995; 25:565–589. 10.1007/BF01582025 7494636

[71] Carter C.W. Coding of Class I and II aminoacyl-tRNA synthetases. Protein Rev. 2017; 18:103–148. 10.1007/5584_2017_93 PMC5927602 28828732

[72] Martinez-Rodriguez L. , Erdogan O., Jimenez-Rodriguez M., Gonzalez-Rivera K., Williams T., Li L., Weinreb V., Collier M., Chandrasekaran S.N., Ambroggio X. et al. Functional class I and II amino acid-activating enzymes can be coded by opposite strands of the same gene. J. Biol. Chem. 2015; 290:19710–19725. 26088142 10.1074/jbc.M115.642876 PMC4528134

[73] Onodera K. , Suganuma N., Takano H., Sugita Y., Shoji T., Minobe A., Yamaki N., Otsuka R., Mutsuro-Aoki H., Umehara T. et al. Amino acid activation analysis of primitive aminoacyl-tRNA synthetases encoded by both strands of a single gene using the malachite green assay. BioSystems. 2021; 208:104481. 34245865 10.1016/j.biosystems.2021.104481

[74] Tang G.Q. , Elder J.J., Douglas J., Carter C.W. Jr Domain acquisition by Class I Aminoacyl-tRNA synthetase urzymes coordinated the catalytic functions of HVGH and KMSKS motifs. Nucleic Acids Res. 2023; 51:8070–8084. 37470821 10.1093/nar/gkad590 PMC10450160

[75] Klipcan L. , Moor N., Kessler N., Safro M.G. Eukaryotic cytosolic and mitochondrial phenylalanyl-tRNA synthetases catalyze the charging of tRNA with the meta-tyrosine. Proc. Natl. Acad. Sci. U.S.A. 2009; 106:11045–11048. 19549855 10.1073/pnas.0905212106 PMC2700156

[76] Pham Y. , Li L., Kim A., Erdogan O., Weinreb V., Butterfoss G.L., Kuhlman B., Carter C.W. Jr A minimal TrpRS catalytic domain supports sense/antisense ancestry of class I and II aminoacyl-tRNA synthetases. Molecular cell. 2007; 25:851–862. 17386262 10.1016/j.molcel.2007.02.010

[77] Cavarelli J. , Delagoutte B., Eriani G., Gangloff J., Moras D. L-arginine recognition by yeast arginyl-tRNA synthetase. EMBO J. 1998; 17:5438–5448. 9736621 10.1093/emboj/17.18.5438 PMC1170869

[78] Zhou X.-L. , Zhu B., Wang E.-D. The CP2 domain of leucyl-tRNA synthetase is crucial for amino acid activation and post-transfer editing. J. Biol. Chem. 2008; 283:36608–36616. 18955487 10.1074/jbc.M806745200 PMC2662312

[79] Nureki O. , Kohno T., Sakamoto K., Miyazawa T., Yokoyama S. Chemical modification and mutagenesis studies on zinc binding of aminoacyl-tRNA synthetases. J. Biol. Chem. 1993; 268:15368–15373. 8340367

[80] Sugiura I. , Nureki O., Ugaji-Yoshikawa Y., Kuwabara S., Shimada A., Tateno M., Lorber B., Giegé R., Moras D., Yokoyama S. et al. The 2.0 Å crystal structure of Thermus thermophilus methionyl-tRNA synthetase reveals two RNA-binding modules. Structure. 2000; 8:197–208. 10673435 10.1016/s0969-2126(00)00095-2

[81] Yaremchuk A. , Tukalo M., Grøtli M., Cusack S. A succession of substrate induced conformational changes ensures the amino acid specificity of Thermus thermophilus prolyl-tRNA synthetase: comparison with histidyl-tRNA synthetase. J. Mol. Biol. 2001; 309:989–1002. 11399074 10.1006/jmbi.2001.4712

[82] Moor N. , Kotik-Kogan O., Tworowski D., Sukhanova M., Safro M. The crystal structure of the ternary complex of phenylalanyl-tRNA synthetase with tRNAPhe and a phenylalanyl-adenylate analogue reveals a conformational switch of the CCA end. Biochemistry. 2006; 45:10572–10583. 16939209 10.1021/bi060491l

[83] Naganuma M. , Sekine S.-I., Fukunaga R., Yokoyama S. Unique protein architecture of alanyl-tRNA synthetase for aminoacylation, editing, and dimerization. Proc. Natl. Acad. Sci. 2009; 106:8489–8494. 19423669 10.1073/pnas.0901572106 PMC2689022

[84] Tan K. , Zhou M., Zhang R., Anderson W.F., Joachimiak A. The crystal structures of the α-subunit of the α 2 β 2 tetrameric Glycyl-tRNA synthetase. J. Struct. And Funct. Genomics. 2012; 13:233–239. 23054484 10.1007/s10969-012-9142-6 PMC3691008

[85] Pauling L. The Probability of Errors in the Process of Synthesis of Protein Molecules. 1957; Birkhauser.

[86] Brooks D.J. , Fresco J.R., Lesk A.M., Singh M. Evolution of amino acid frequencies in proteins over deep time: inferred order of introduction of amino acids into the genetic code. Mol. Biol. Evol. 2002; 19:1645–1655. 12270892 10.1093/oxfordjournals.molbev.a003988

[87] Ribeiro A.J. , Tyzack J.D., Borkakoti N., Holliday G.L., Thornton J.M. A global analysis of function and conservation of catalytic residues in enzymes. J. Biol. Chem. 2020; 295:314–324. 31796628 10.1074/jbc.REV119.006289 PMC6956550

[88] Kovacs N.A. , Petrov A.S., Lanier K.A., Williams L.D. Frozen in time: the history of proteins. Mol. Biol. Evol. 2017; 34:1252–1260. 28201543 10.1093/molbev/msx086 PMC5400399

[89] Sapienza P.J. , Li L., Williams T., Lee A.L., Carter C.W. Jr An ancestral tryptophanyl-tRNA synthetase precursor achieves high catalytic rate enhancement without ordered ground-state tertiary structures. ACS Chem. Biol. 2016; 11:1661–1668. 27008438 10.1021/acschembio.5b01011 PMC5461432

[90] Li Z. , Carter C. Aminoacyl-tRNA synthetases may have evolved from molten globular precursors. Acta Crystallographica A-Foundation and Advances. Vol. 75. Int Union Crystallography 2 Abbey SQ. 2019; Chester, CH1 2HU, England A98.

[91] Mullen G. , Vaughn J., Mildvan A. Sequential proton NMR resonance assignments, circular dichroism, and structural properties of a 50-residue substrate-binding peptide from DNA polymerase I. Arch. Biochem. Biophys. 1993; 301:174–183. 8442659 10.1006/abbi.1993.1130

[92] Chuang W.J. , Abeygunawardana C., Pedersen P.L., Mildvan A.S. Two-dimensional NMR, circular dichroism, and fluorescence studies of PP-50, a synthetic ATP-binding peptide from the. beta.-subunit of mitochondrial ATP synthase. Biochemistry. 1992; 31:7915–7921. 1387322 10.1021/bi00149a024

[93] Chuang W.-J. , Abeygunawardana C., Gittis A.G., Pedersen P.L., Mildvan A.S. Solution structure and function in trifluoroethanol of PP-50, an ATP-binding peptide from F1ATPase. Arch. Biochem. Biophys. 1995; 319:110–122. 7771774 10.1006/abbi.1995.1272

[94] Bridgham J.T. , Ortlund E.A., Thornton J.W. An epistatic ratchet constrains the direction of glucocorticoid receptor evolution. Nature. 2009; 461:515–519. 19779450 10.1038/nature08249 PMC6141187

[95] Widmann J. , Harris J.K., Lozupone C., Wolfson A., Knight R. Stable tRNA-based phylogenies using only 76 nucleotides. Rna. 2010; 16:1469–1477. 20558546 10.1261/rna.726010 PMC2905747

[96] Force A. , Lynch M., Pickett F.B., Amores A., Yan Y.-L., Postlethwait J. Preservation of duplicate genes by complementary, degenerative mutations. Genetics. 1999; 151:1531–1545. 10101175 10.1093/genetics/151.4.1531 PMC1460548

[97] Fournier G.P. , Andam C.P., Alm E.J., Gogarten J.P. Molecular evolution of aminoacyl tRNA synthetase proteins in the early history of life. Origins Life Evol. B. 2011; 41:621–632. 10.1007/s11084-011-9261-2 22200905

[98] Fournier G.P. , Alm E. Ancestral reconstruction of a pre-LUCA aminoacyl-tRNA synthetase ancestor supports the late addition of Trp to the genetic code. J. Mol. Evol. 2015; 80:171–185. 25791872 10.1007/s00239-015-9672-1

[99] Terwilliger T.C. , Liebschner D., Croll T.I., Williams C.J., McCoy A.J., Poon B.K., Afonine P.V., Oeffner R.D., Richardson J.S., Read R.J. et al. AlphaFold predictions are valuable hypotheses, and accelerate but do not replace experimental structure determination. 2022; bioRxiv doi:19 May 2023, preprint: not peer reviewed 10.1101/2022.11.21.517405. PMC10776388 38036854

[100] Wills P.R. , Nieselt K., McCaskill J.S. Emergence of coding and its specificity as a physico-informatic problem. Origins Life Evol. B. 2015; 45:249–255. 10.1007/s11084-015-9434-5 25813662

[101] Shore J.A. , Holland B.R., Sumner J.G., Nieselt K., Wills P.R. The ancient operational code is embedded in the amino acid substitution matrix and aaRS phylogenies. J. Mol. Evol. 2020; 88:136–150. 31781936 10.1007/s00239-019-09918-z

[102] Sekine S.-I. , Nureki O., Dubois D.Y., Bernier S., Chênevert R., Lapointe J., Vassylyev D.G., Yokoyama S. ATP binding by glutamyl-tRNA synthetase is switched to the productive mode by tRNA binding. EMBO J. 2003; 22:676–688. 12554668 10.1093/emboj/cdg053 PMC140737

[103] Hill K. , Schimmel P. Evidence that the 3’-end of a transfer RNA binds to a site in the adenylate synthesis domain of an aminoacyl-tRNA synthetase. Biochemistry. 1989; 28:2577–2586. 2543446 10.1021/bi00432a035

[104] Kamtekar S. , Hohn M.J., Park H.-S., Schnitzbauer M., Sauerwald A., Söll D., Steitz T.A. Toward understanding phosphoseryl-tRNACys formation: the crystal structure of Methanococcus maripaludis phosphoseryl-tRNA synthetase. Proc. Natl. Acad. Sci. U.S.A. 2007; 104:2620–2625. 17301225 10.1073/pnas.0611504104 PMC1815232

[105] Arnez J. , Harris D., Mitschler A., Rees B., Francklyn C., Moras D. Crystal structure of histidyl-tRNA synthetase from Escherichia coli complexed with histidyl-adenylate. EMBO J. 1995; 14:4143–4155. 7556055 10.1002/j.1460-2075.1995.tb00088.x PMC394497

[106] Torres-Larios A. , Sankaranarayanan R., Rees B., Dock-Bregeon A.-C., Moras D. Conformational movements and cooperativity upon amino acid, ATP and tRNA binding in threonyl-tRNA synthetase. J. Mol. Biol. 2003; 331:201–211. 12875846 10.1016/s0022-2836(03)00719-8

[107] Rock F.L. , Mao W., Yaremchuk A., Tukalo M., Crépin T., Zhou H., Zhang Y.-K., Hernandez V., Akama T., Baker S.J. et al. An antifungal agent inhibits an aminoacyl-tRNA synthetase by trapping tRNA in the editing site. science. 2007; 316:1759–1761. 17588934 10.1126/science.1142189

[108] Gaston M.A. , Zhang L., Green-Church K.B., Krzycki J.A. The complete biosynthesis of the genetically encoded amino acid pyrrolysine from lysine. Nature. 2011; 471:647–650. 21455182 10.1038/nature09918 PMC3070376

