# Supplementary material for: Enzymic recognition of amino acids drove the evolution of primordial genetic codes
Source: Nucleic Acids Res. 2023 Dec 4;52(2):558–71. doi: 10.1093/nar/gkad1160 (PMC10810186; doi:10.1093/nar/gkad1160)
Supplement: gkad1160_supplemental_file [file gkad1160_supplemental_file.pdf]

# Enzymic recognition of amino acids drove the evolution of primordial genetic codes

Jordan Douglas[1,*], Remco Bouckaert[2], Charles W. Carter, Jr [3] , Peter R. Wills[1]

[1]Department of Physics, The University of Auckland, New Zealand
[2]School of Computer Science, The University of Auckland, New Zealand
[3]Department of Biochemistry and Biophysics, University of North Carolina at Chapel Hill, USA
[*]jordan.douglas@auckland.ac.nz

October 27, 2023

## Supporting information

### Identification of AARS Families

To identify the 36 AARS catalytic domain families, we inferred the phylogenies of Class I and II catalytic domains using BEAST 2 [1]. These analyses, as described in Materials and Methods of the main article, were performed in order to identify families used in subsequent analyses and were based on an alignment of structural elements common to the entire class. These elements are: S1-S5 and H1-H5 for Class I, and S1-S5, H1-H3, SH1 and Motif 1 for Class II. The summary trees are in **Fig. S3** and **Fig. S2** demonstrate a high level of posterior support for the families identified. The families are summarised in **Table S1**. Most families are monophyletic with over 99% support. However, the following families have less than 75% support.

1. Class I ValRS has 56% clade support. The Archaeal form is distinct from the Bacterial and Eukaryotic form. With the remaining 44% support, these two ValRS clades belong to distinct parts of the subclass Ia clade.

2. Class II GlyRS-A is not monophyletic because it contains GlyRS-E. However, the two families combined are monophyletic with over 99% support.

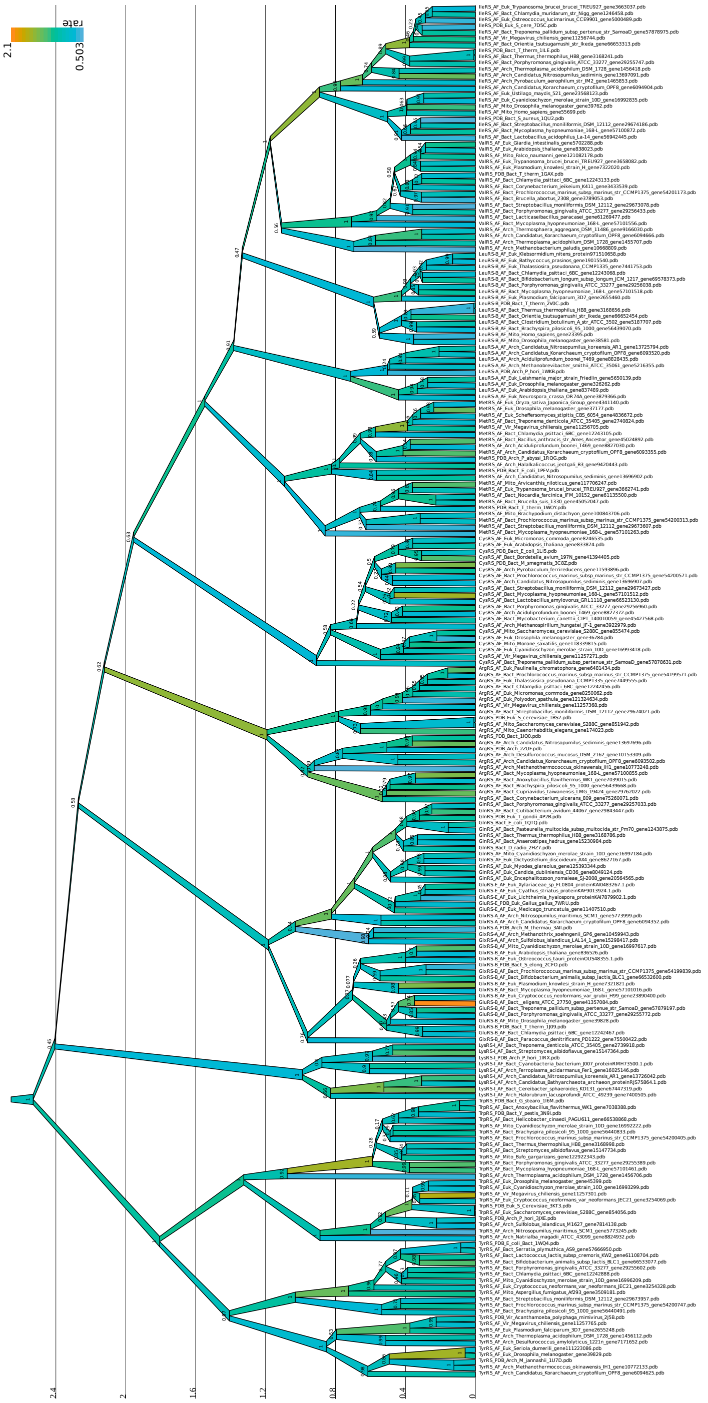


Fig. S1: Phylogeny of the Class I common catalytic domain sequence elements. This summary tree is the maximum clade credibility [2] tree with internal nodes labelled by posterior clade support and node heights are in substitutions per site. Branches are coloured by relative susbsitution rate under the relaxed clock model [3]. Leaves annotated with 'PDB' refer to experimentally solved structures.

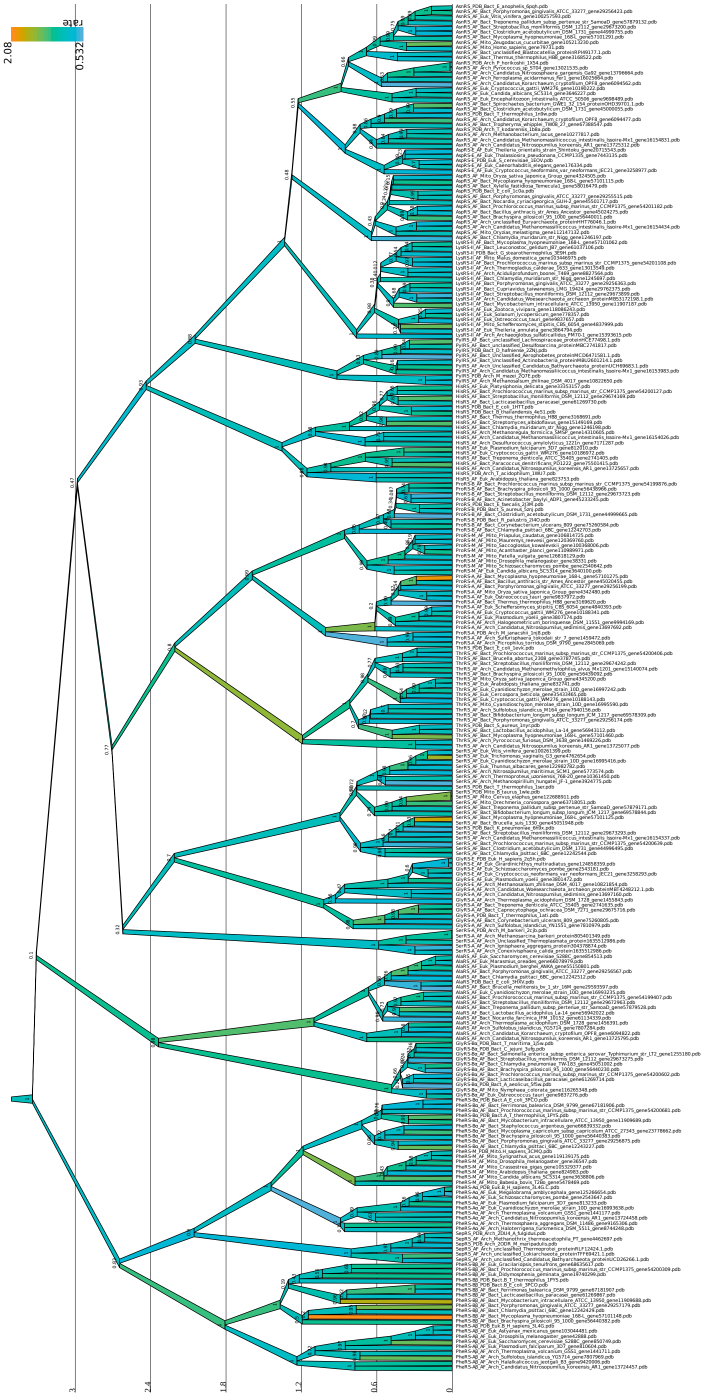

Fig. S2: Phylogeny of the Class II common catalytic domain sequence elements. See **Fig. S3** caption. Note the low posterior clade support near the root of the tree, especially related to the placement of PheRS and SepRS as part of the outgroup (as opposed to AlaRS and GlyRS-B).

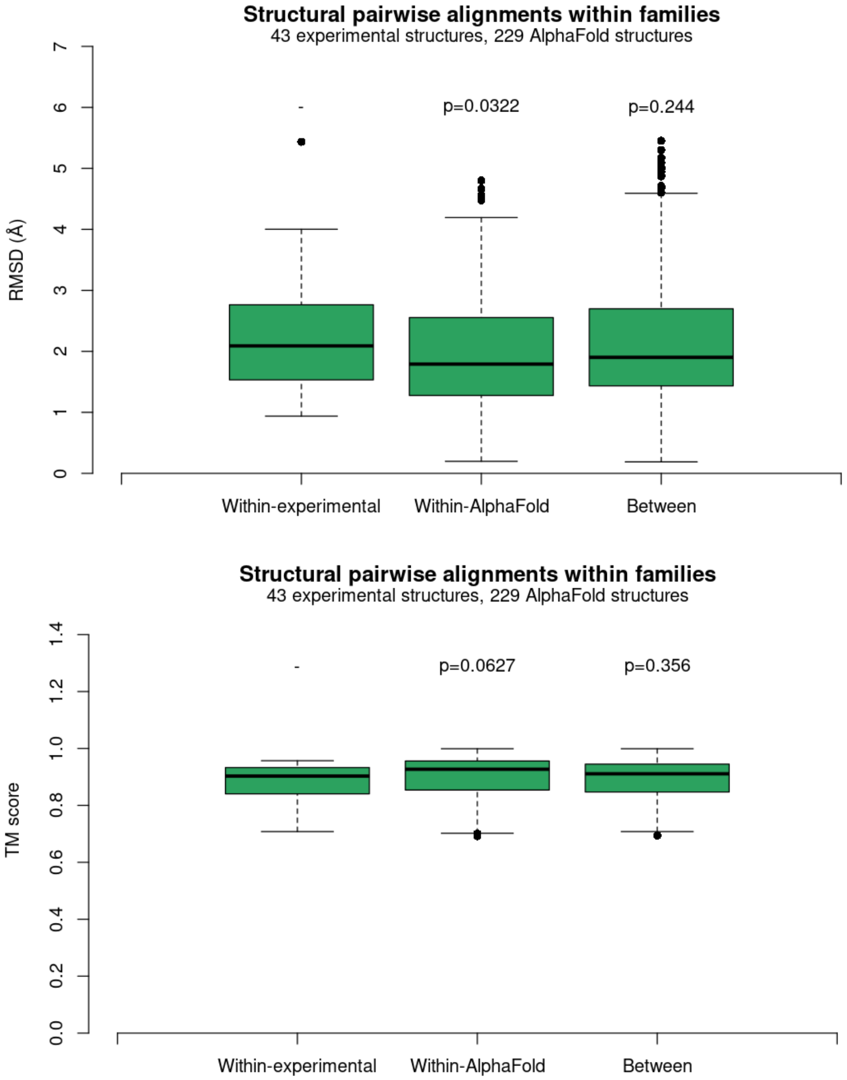


Fig. S3: Distances were calculated from alignments between experimentally solved structures, between AlphaFold structures, or across the two groups, using DeepAlign [4] on pairs of members from the same AARS catalytic domain family. P-values are from two-sided Student t-tests which compares the pairwise distances of each group with those of within-experimental. These results suggest that the AlphaFold structures are generally quite similar to the experimentally determined structures, and that the variation among experimentally determined structures is not significantly different to the variation among AlphaFold structures, or between the two groups. We performed this test on all AARS families with at least 2 experimentally solved structures from at least 2 species. These PDB structures are: ArgRS: 2zuf, 1iq0, 1bs2; CysRS: 1li5, 3c8z; IleRS: 1qu2, 1ile, 7d5c; MetRS: 1rqg, 1pfv, 1woy; TrpRS :3jxe, 3kt3, 1i6m, 3n9i; TyrRS: 1u7d, 1wq4, 2j5b; AsnRS: 1x54, 6pqh, 1asz; AsxRS: 1b8a, 1n9w, 1eov; GluRS-B: 5f5w, 3ufg, 1j5w; HisRS: 1wu7, 4e51, 1htt; PheRS-B :3pco, 1pys; ProRS-B: 5znj,2j3m; PylRS: 2q7e, 2znj; SepRS: 2odr, 2du4; SerRS: 6h9x, 1ser, 1wle; ThrRS: 1evk, 1nyr.

| **Class** | **Subclass** | **Family (PDB structure)** | $P_{\text{S}}$ | $P_{\text{SM}}$ |
|---|---|---|---|---|
| I | a | CysRS (1li5), IleRS (7d5c), LeuRS-A (1wkb), LeuRS-B (2v0c), MetRS (1pfv), ValRS (1gax) | 0.79 | 0.81 |
| | b | GlnRS (1qtq), GluRS-B (1j09), GlxRS-B (2cfo), GluRS-E (7wru), GlxRS-A (3aii) | 0.98 | 0.98 |
| | c | TrpRS (1i6m), TyrRS (2j5b) | 0.95 | 0.94 |
| | d | ArgRS (1bs2) | 1 | 1 |
| | e | LysRS-I (1irx) | 1 | 1 |
| II | a | GlyRS-A (1ati), GlyRS-E (2q5h), ProRS-A (1nj8), ProRS-B (2j3m), ProRS-M, SerRS (1wle), SerRS-A (2cjb), ThrRS (1nyr) | 0.62 | 0.88 |
| | b | AsnRS (6pqh), AspRS (1c0a), AsxRS (1eov), LysRS-II (3e9h) | 0.98 | 0.96 |
| | c | PheRS-M (3cmq) , PheRS-A$\alpha$ (3l4g), PheRS-B$\alpha$ (3pco), SepRS (2odr), HisRS (1htt) | 0.77 | 0.76 |
| | d | AlaRS (3hxv), GlyRS-B (1j5w) | 0.99 | 0.99 |
| | e | PylRS (2q7e) | 1 | 1 |

Table S1: AARS catalytic domain families and an example of an experimentally solved structure. There are no solved structures for ProRS-M. The estimated clade supports $P$ are shown, where $P_{\text{S}}$ was calculated using only sequence information, and $P_{\text{SM}}$ using both sequences and insertion modules. The inclusion of singletons (such as ArgRS and LysRS-I) into other subclasses was found to significantly reduce their supports and therefore they are best identified as singletons.

| **Parameter** | **Prior** | **Posterior (Class I)** | **Posterior (Class II)** |
|---|---|---|---|
| Family tree height | Yule prior | 2.02 (1.7,2.37) | 2.54 (2.09,3.03) |
| Yule birth rate | LN(-0.5,1) | 0.775 (0.437,1.2) | 0.711 (0.419,1.02) |
| Relaxed clock standard deviation | Gamma(4,0.05) | 0.445 (0.363,0.557) | 0.411 (0.323,0.496) |
| Amino acid substitution rate | 1 | 1 | 1 |
| Insertion module birth rate | LN(-5,2) | 0.0513 (0.026,0.0799) | 0.0754 (0.0388,0.107) |
| Insertion module death rate | LN(-7.3,2) | 0.00783 (0.00392,0.0126) | 0.0106 (0.0057,0.0162) |
| Substitution model indicator | Uniform({ 0,1,...,14 }) | 13 {13,13} | 13 {13,13} |
| Using gamma rate heterogeneity? | Uniform([0,1]) | 1 {1,1} | 1 {1,1} |
| Estimating invariant proportion? | Uniform([0,1]) | 1 {1,1} | 1 {1,1} |
| Gamma rate heterogeneity shape | Exponential(1) | 2.21 (2.08,2.38) | 1.98 (1.78,2.13) |
| Proportion of invariant sites | Beta(1,4) | 0.00677 (0.00241,0.0107) | 0.0111 (0,0.0193) |

Table S2: Prior and posterior distributions under the Sequence+IM model. Prior distributions: LN: LogNormal distribution with parameters $\mu$ and $\sigma$; gamma and beta distributions with parameters $\alpha$ and $\beta$; exponential distribution with mean; discrete uniform distribution over the specified elements. The gamma shape also has a minimum value of 0.1, and the most commonly estimated subsitution model by OBAMA was model 13 – the VT model [5].

## Class I AlphaFold Confidence Scores

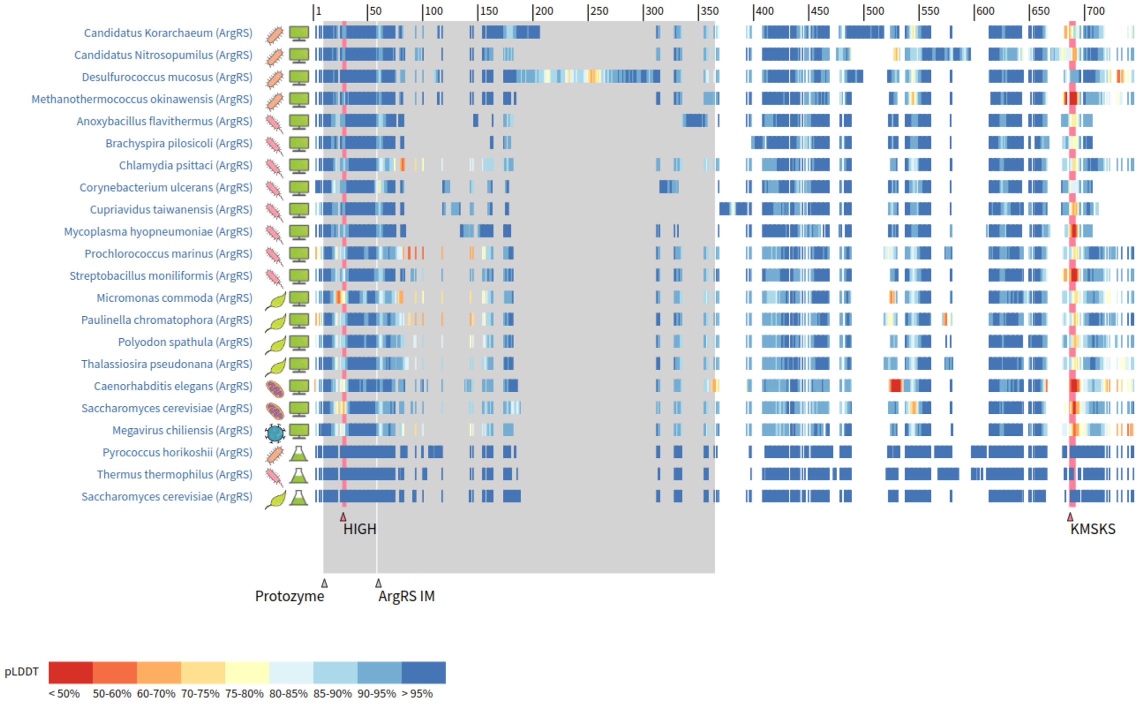


Fig. S4: **ArgRS** distribution of AlphaFold pLDDT scores for the catalytic domain. Each row is a sequence/structure in the alignment, with gaps denoted by empty space. The computers denote AlphaFold structures, while flasks are experimentally solved (and their pLDDT scores are fixed at 100%). The icons after the sequence names denote the domain of life - orange microbe: Archaea; pink microbe: bacteria; green leaf: eukaryotic cytosol; brown/purple organelle: mitochondrion or cholorplast; blue virus: virus. This plot suggests that most regions of the catalytic domain have high confidence, except for the area flanking the KMSKS motif. The median score across AlphaFold structures was 96%, and 90% of all scores were greater than 85%.

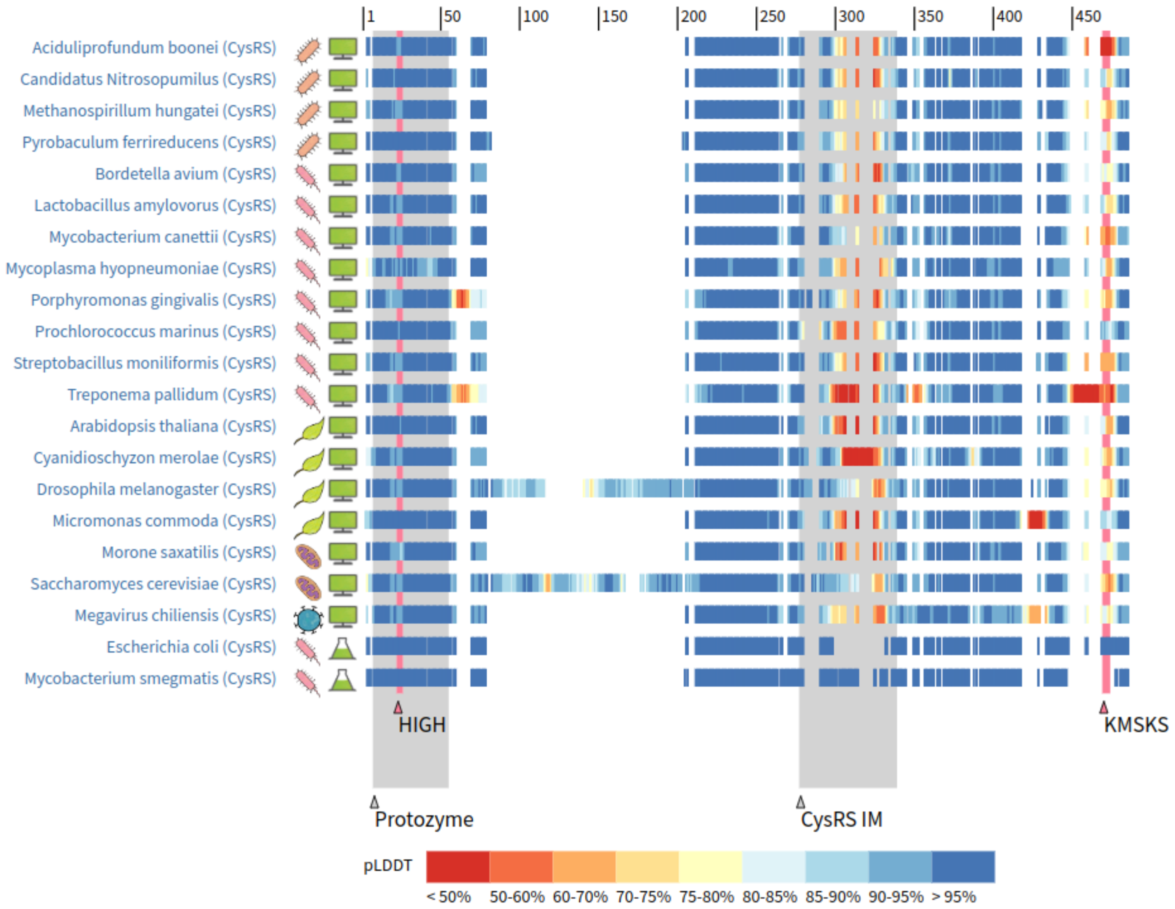


Fig. S5: **CysRS** distribution of AlphaFold pLDDT scores for the catalytic domain. Regions of low confidence: the CysRS insertion module, and the area flanking the KMSKS motif. The median score was 96%, and 90% of all scores were greater than 79%. Refer to Fig. S4 for notation.

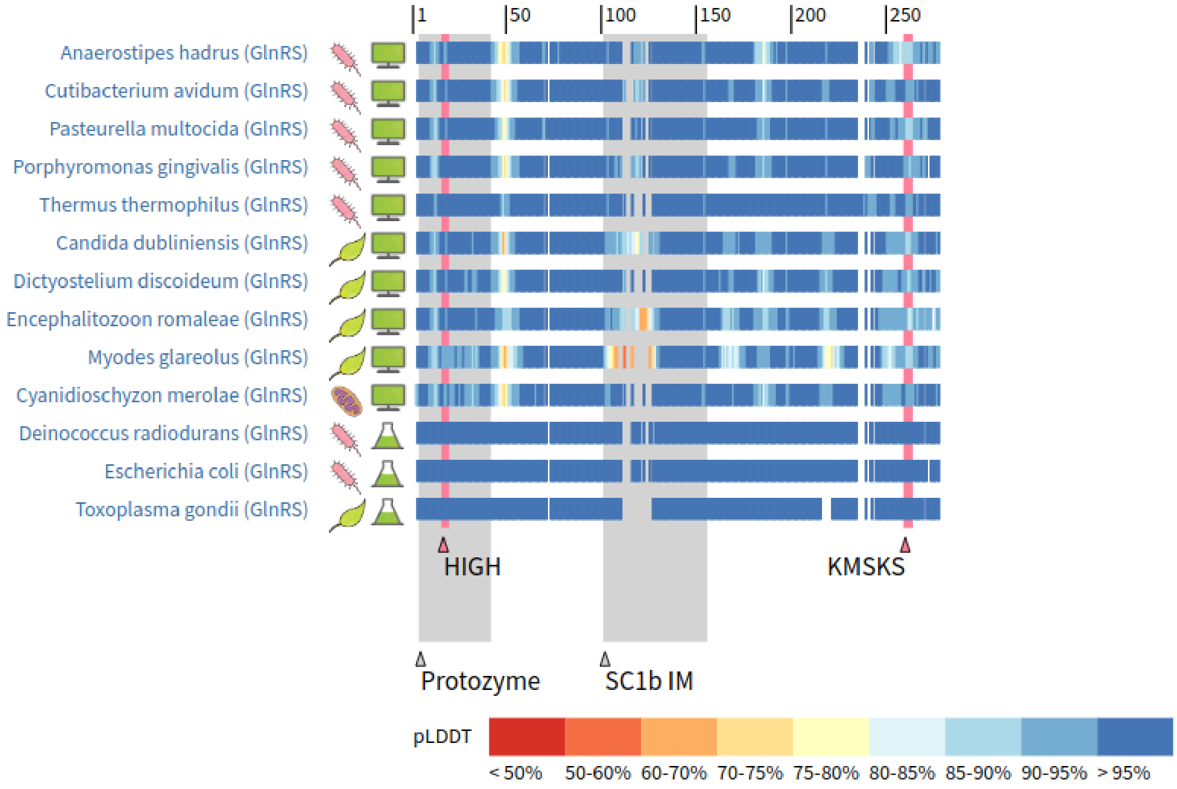


Fig. S6: **GlnRS** distribution of AlphaFold pLDDT scores for the catalytic domain. The median score was 95%, and 90% of all scores were greater than 80%. Refer to Fig. S4 for notation.

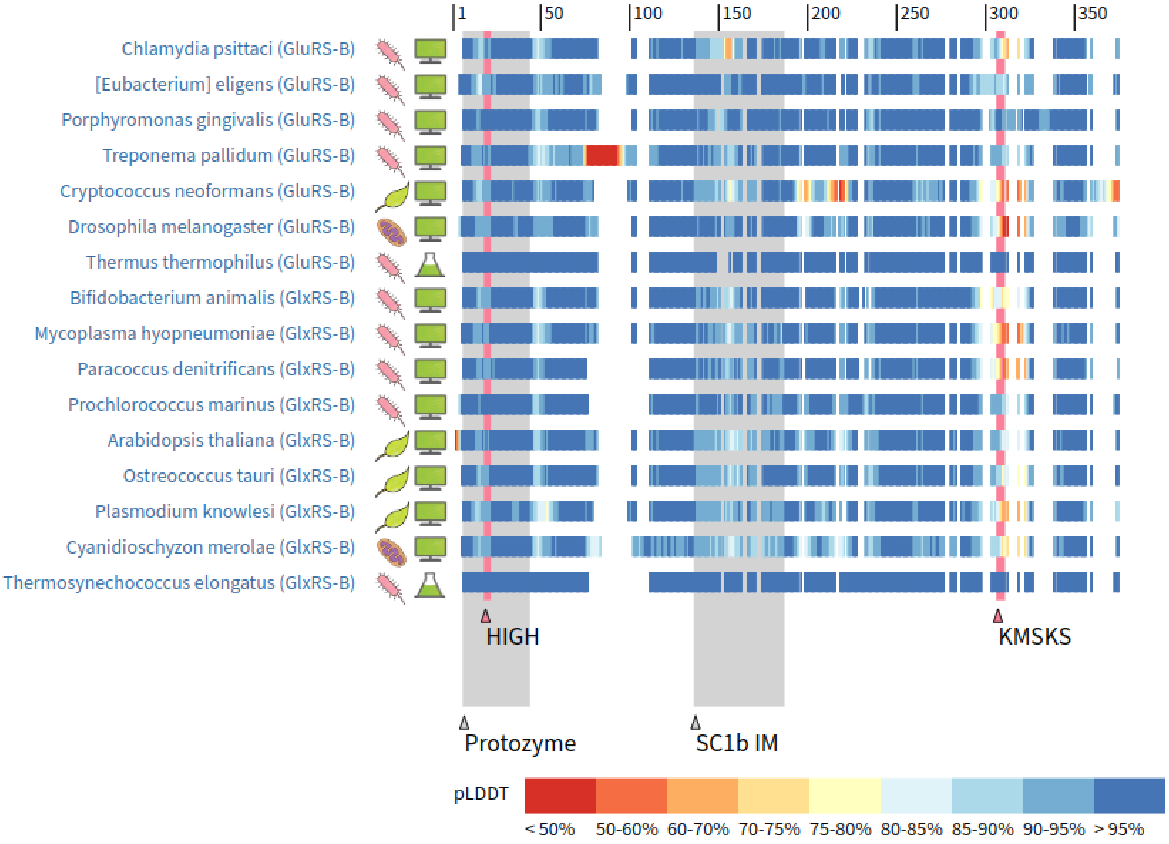


Fig. S7: **GluRS-B and GlxRS-B** distributions of AlphaFold pLDDT scores for the catalytic domain. Regions of low confidence: the area flanking the KMSKS motif. The median score was 95%, and 90% of all scores were greater than 75%. Refer to Fig. S4 for notation.

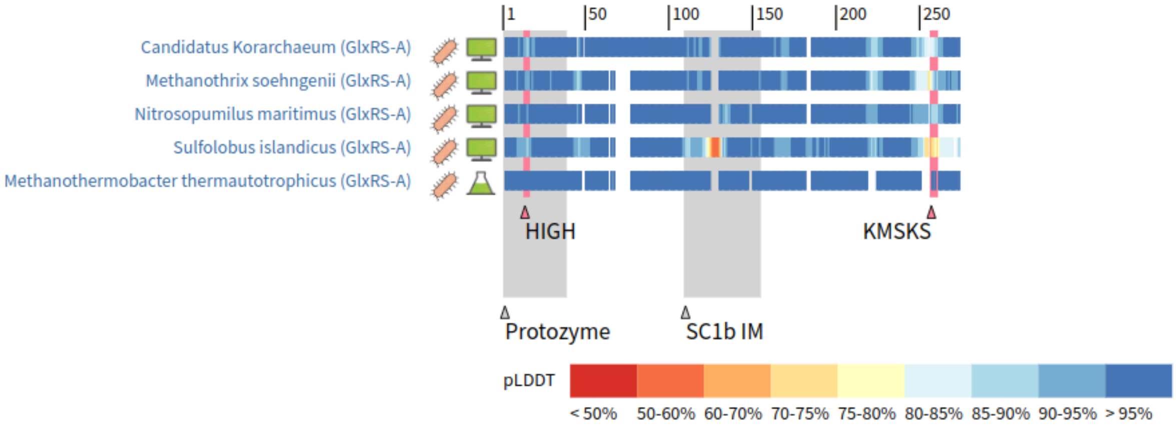


Fig. S8: **GlxRS-A** distribution of AlphaFold pLDDT scores for the catalytic domain. The median score was 95%, and 90% of all scores were greater than 86%. Refer to Fig. S4 for notation.

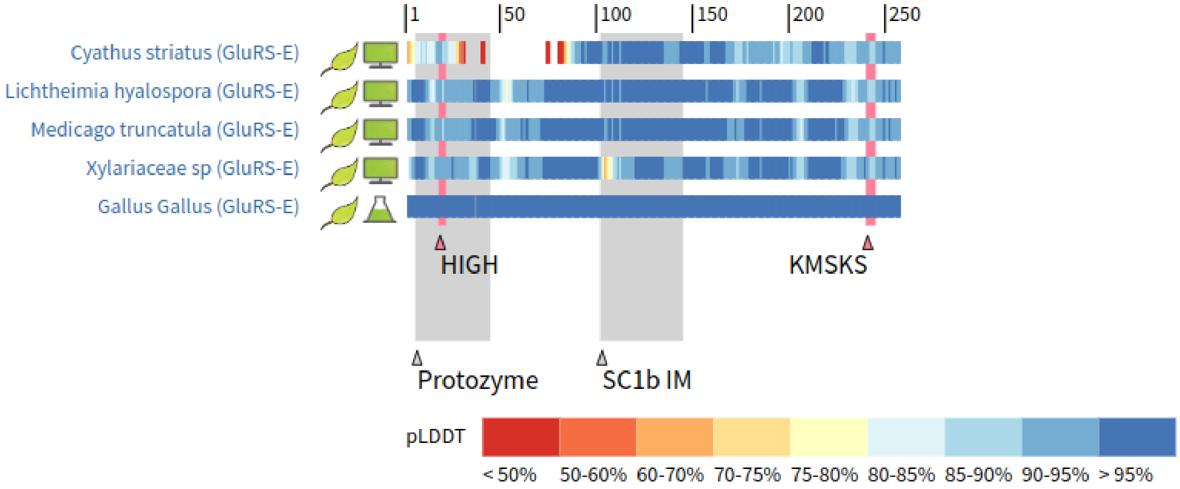


Fig. S9: **GluRS-E** distribution of AlphaFold pLDDT scores for the catalytic domain. The median score was 94%, and 90% of all scores were greater than 83%. Refer to Fig. S4 for notation.

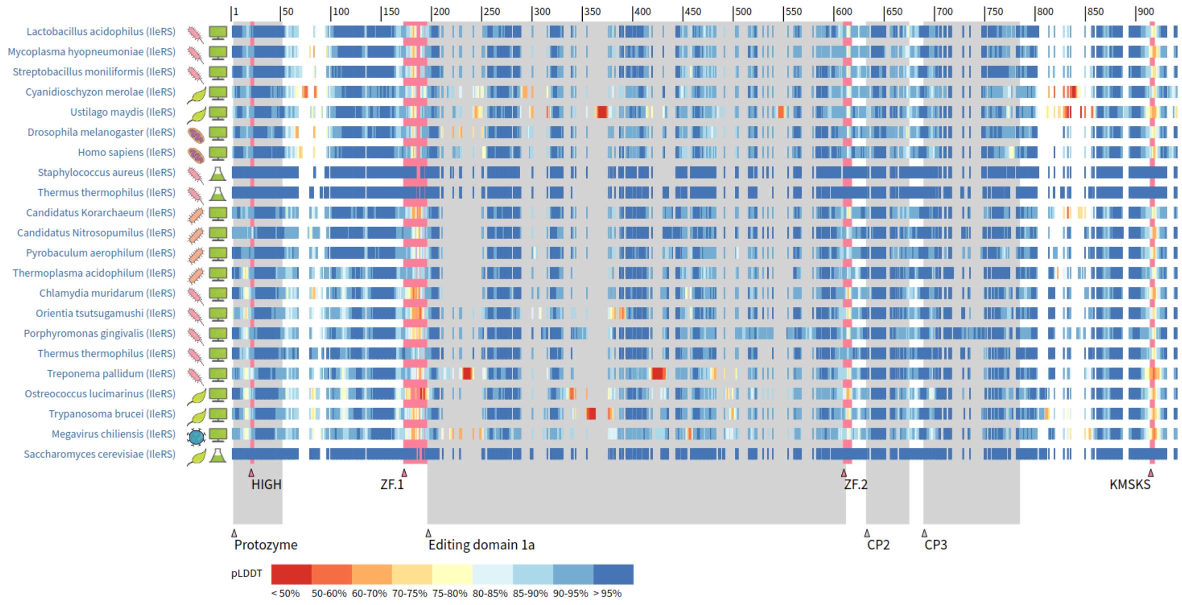


Fig. S10: **IleRS** distribution of AlphaFold pLDDT scores for the catalytic domain. Regions of low confidence: the zinc finger (ZF), and the area flanking the KMSKS motif. The median score was 95%, and 90% of all scores were greater than 83%. Refer to Fig. S4 for notation.

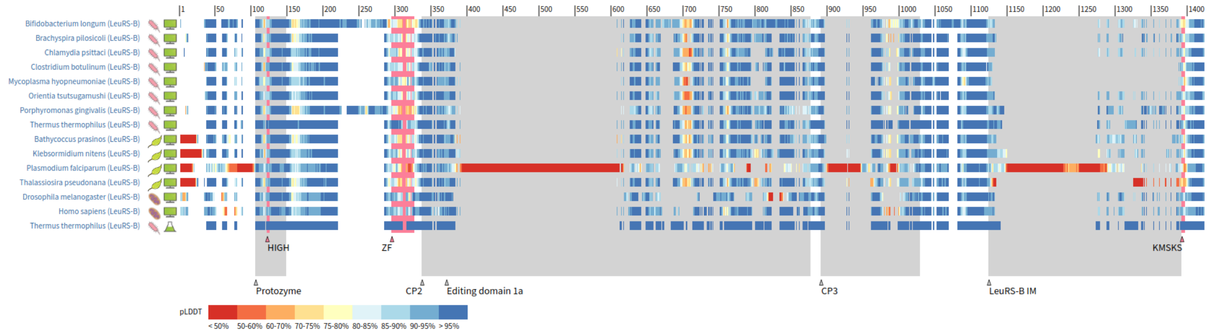


Fig. S11: **LeuRS-B** distribution of AlphaFold pLDDT scores for the catalytic domain. Regions of low confidence: the zinc finger (ZF), the area flanking the KMSKS motif, a short loop in the editing domain, and many putative insertions within *Plasmodium falciparum*. The median score was 94%, and 90% of all scores were greater than 66%. Refer to Fig. S4 for notation.

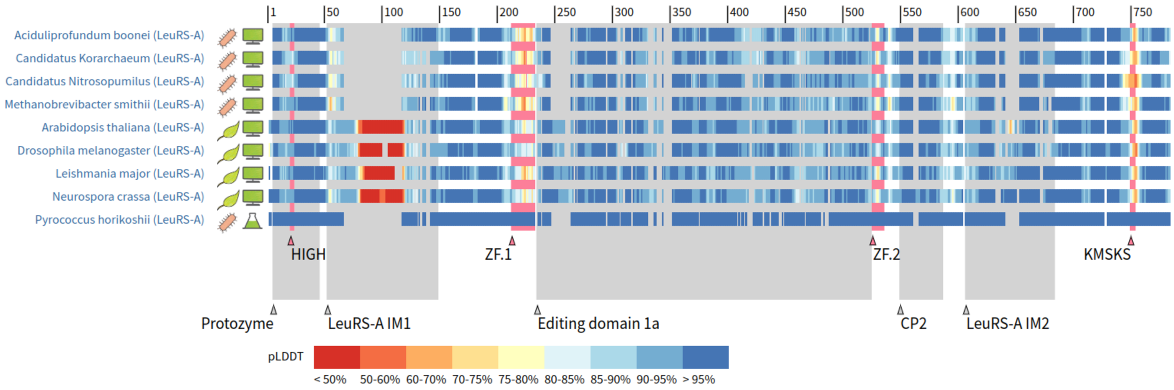


Fig. S12: **LeuRS-A** distribution of AlphaFold pLDDT scores for the catalytic domain. Regions of low confidence: the zinc finger (ZF), the area flanking the KMSKS motif, and a region within the LeuRS-A insertion module (eukaryotes only). The median score was 94%, and 90% of all scores were greater than 83%. Refer to Fig. S4 for notation.

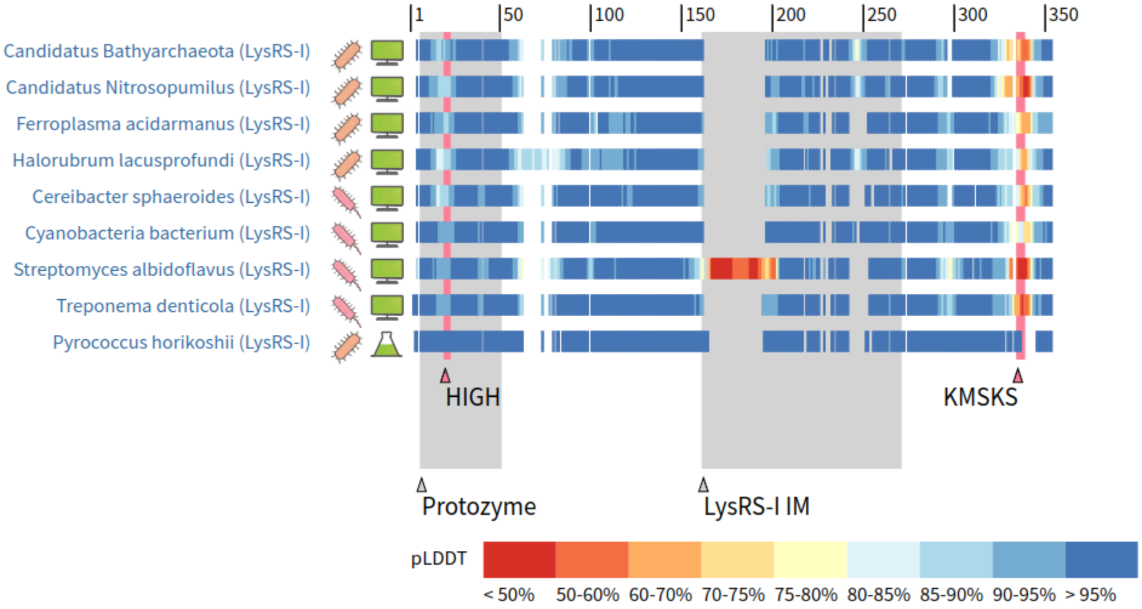


Fig. S13: **LysRS-I** distribution of AlphaFold pLDDT scores for the catalytic domain. Regions of low confidence: the area flanking the KMSKS motif. The median score was 96%, and 90% of all scores were greater than 87%. Refer to Fig. S4 for notation.

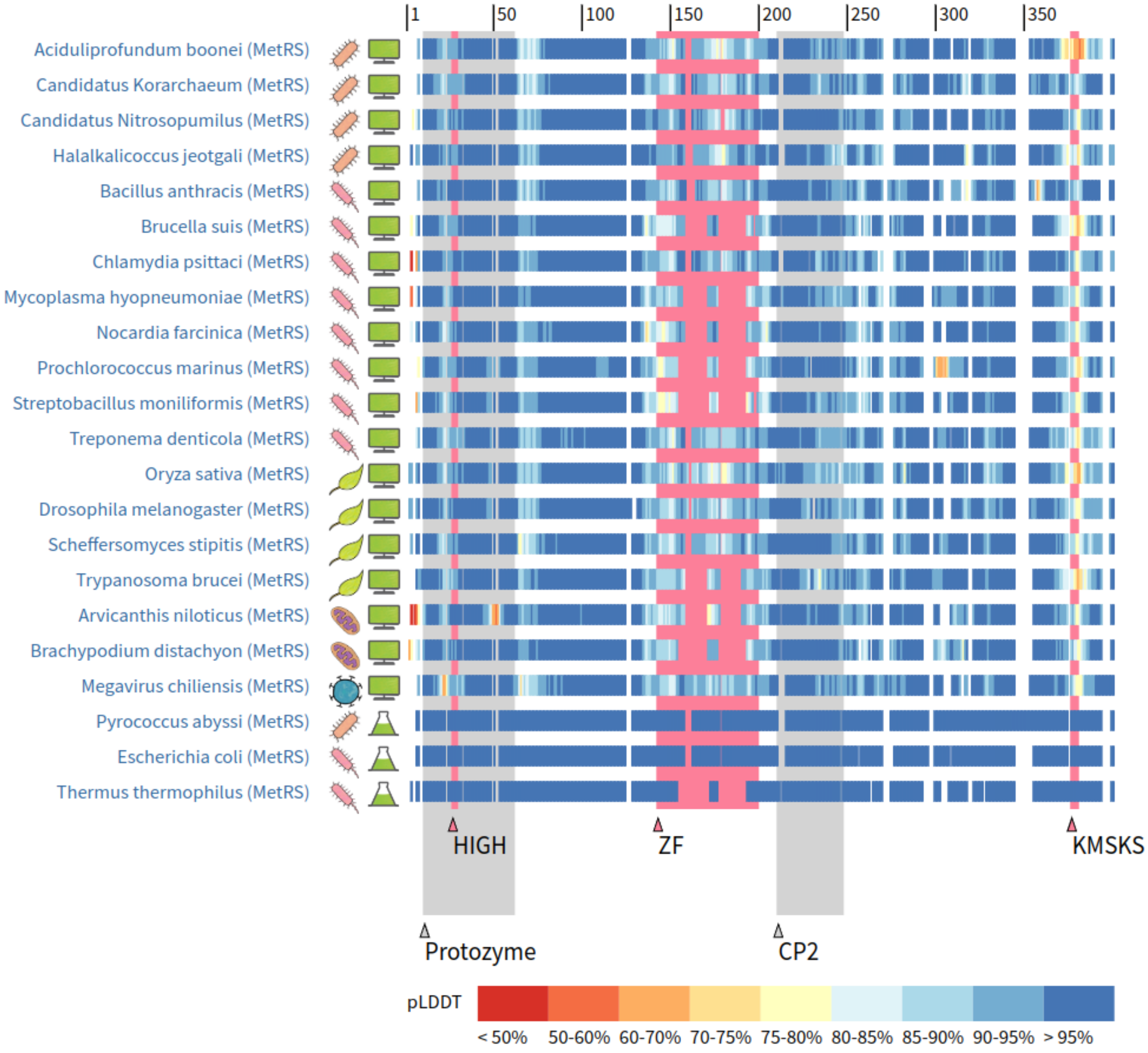


Fig. S14: **MetRS** distribution of AlphaFold pLDDT scores for the catalytic domain. Regions of low confidence: the area flanking the KMSKS motif. The median score was 96%, and 90% of all scores were greater than 85%. Refer to Fig. S4 for notation.

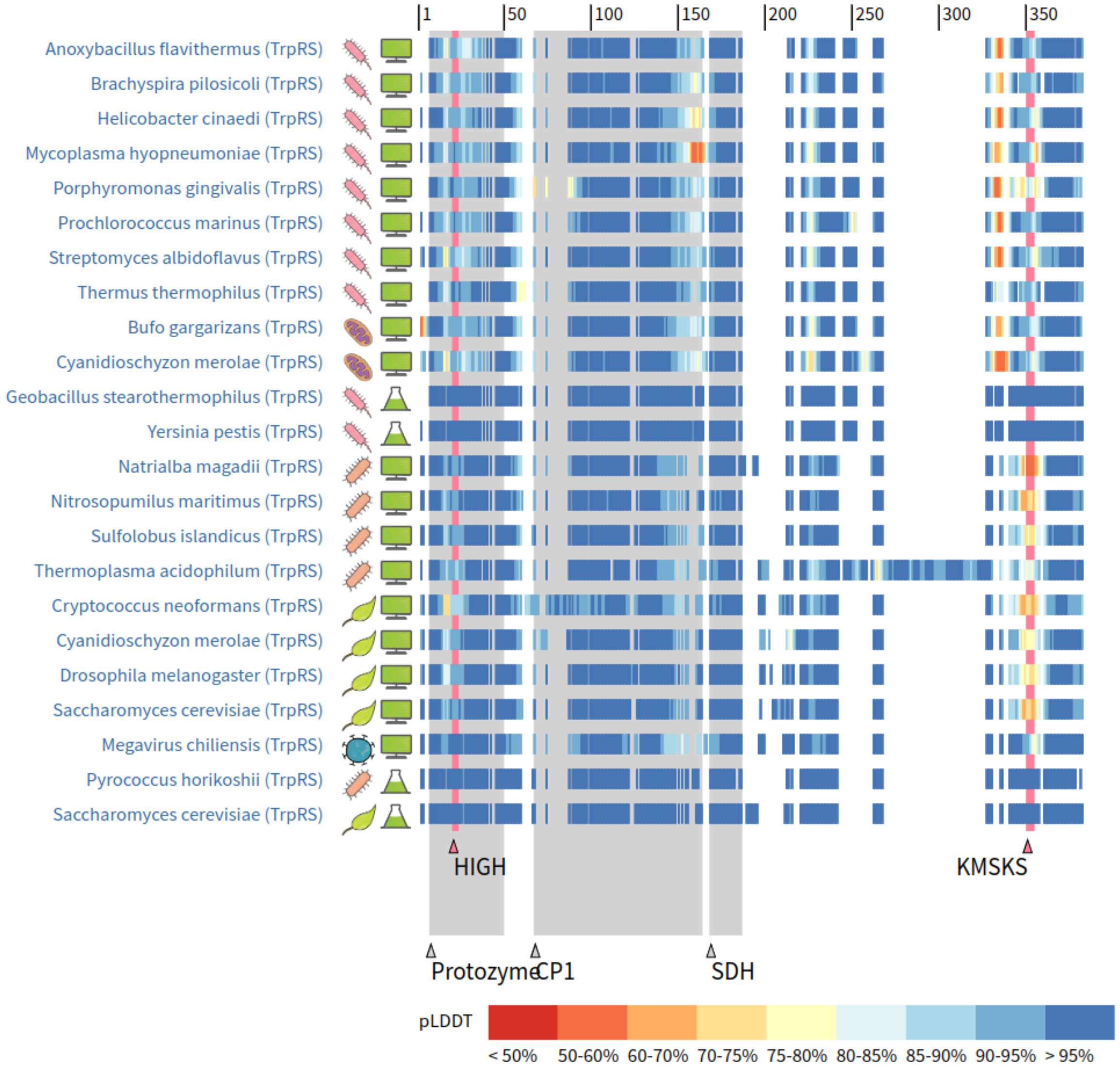


Fig. S15: **TrpRS** distribution of AlphaFold pLDDT scores for the catalytic domain. Regions of low confidence: the area flanking the KMSKS motif. The median score was 96%, and 90% of all scores were greater than 73%. Refer to Fig. S4 for notation.

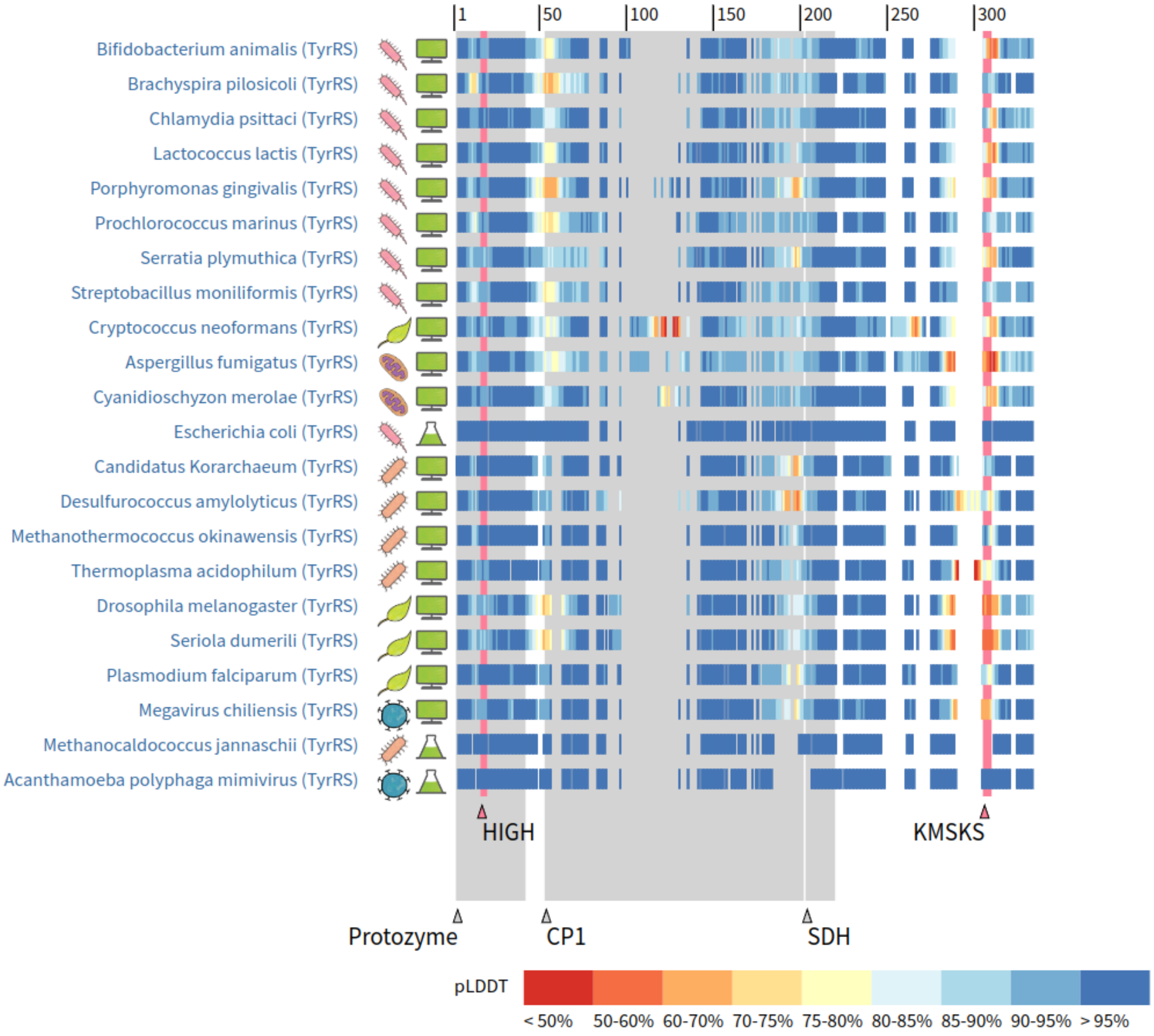


Fig. S16: **TyrRS** distribution of AlphaFold pLDDT scores for the catalytic domain. Regions of low confidence: the area flanking the KMSKS motif, and the N-terminal region of CP1. The median score was 95%, and 90% of all scores were greater than 79%. Refer to Fig. S4 for notation.

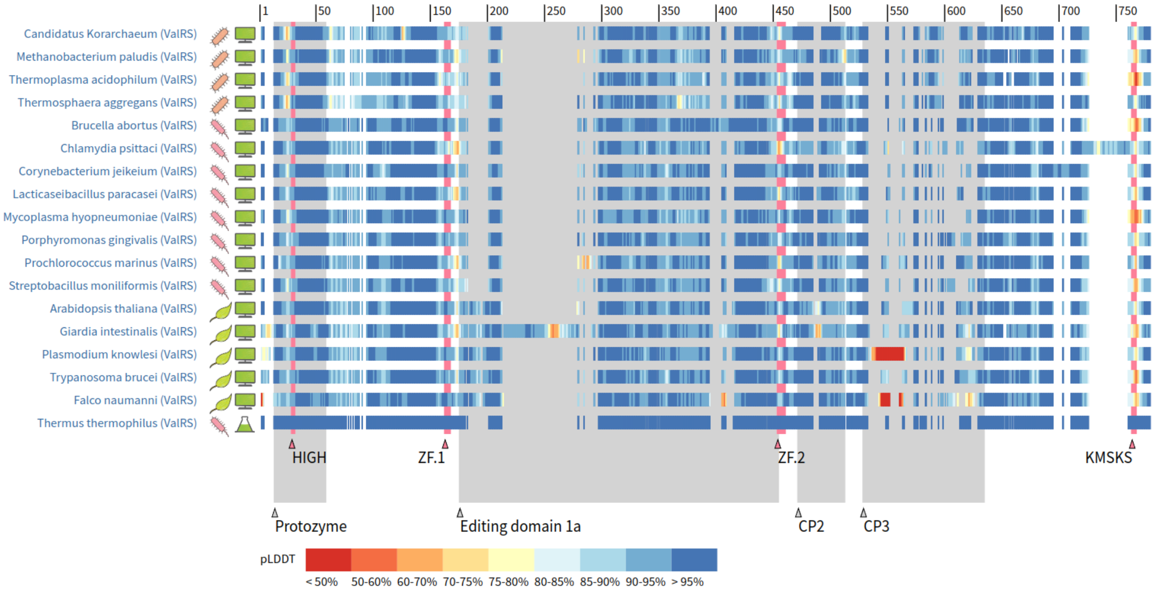


Fig. S17: **ValRS** distribution of AlphaFold pLDDT scores for the catalytic domain. Regions of low confidence: the area flanking the KMSKS motif. The median score was 94%, and 90% of all scores were greater than 82%. Refer to Fig. S4 for notation.

# Class II AlphaFold Confidence Scores

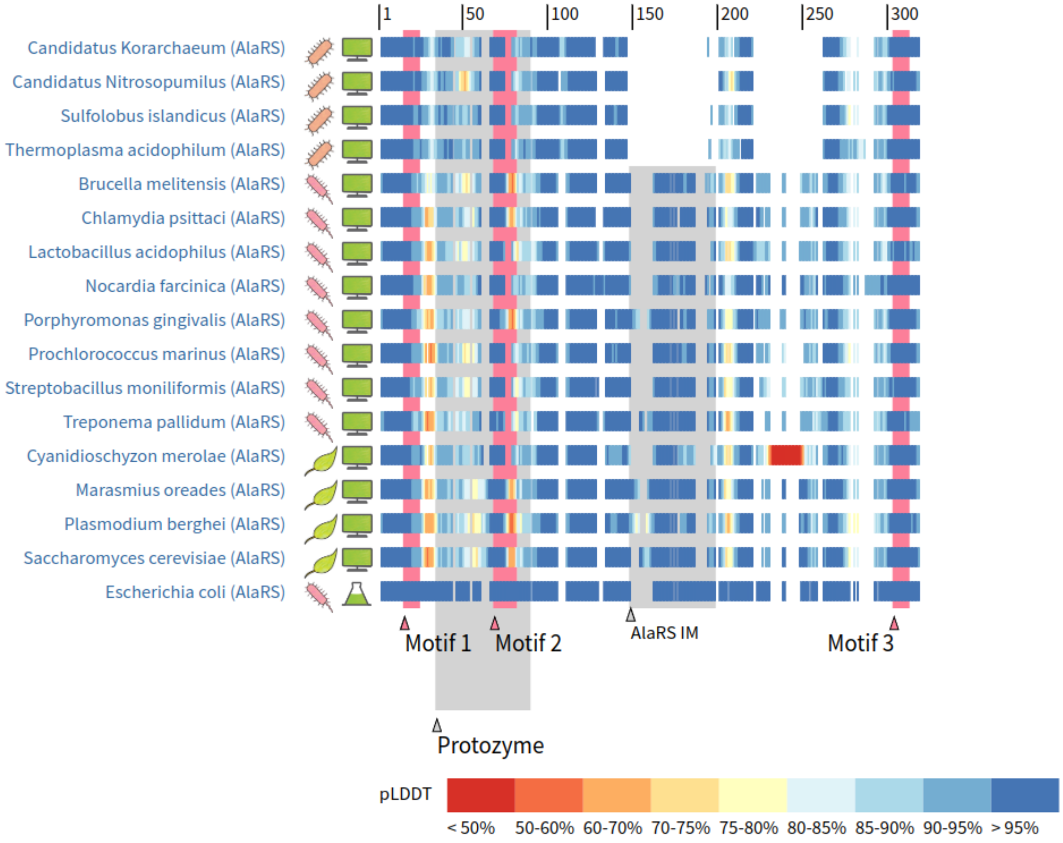


Fig. S18: **AlaRS** distribution of AlphaFold pLDDT scores for the catalytic domain. Regions of low confidence: a short loop downstream from motif 1. The median score was 93%, and 90% of all scores were greater than 76%. Refer to Fig. S4 for notation.

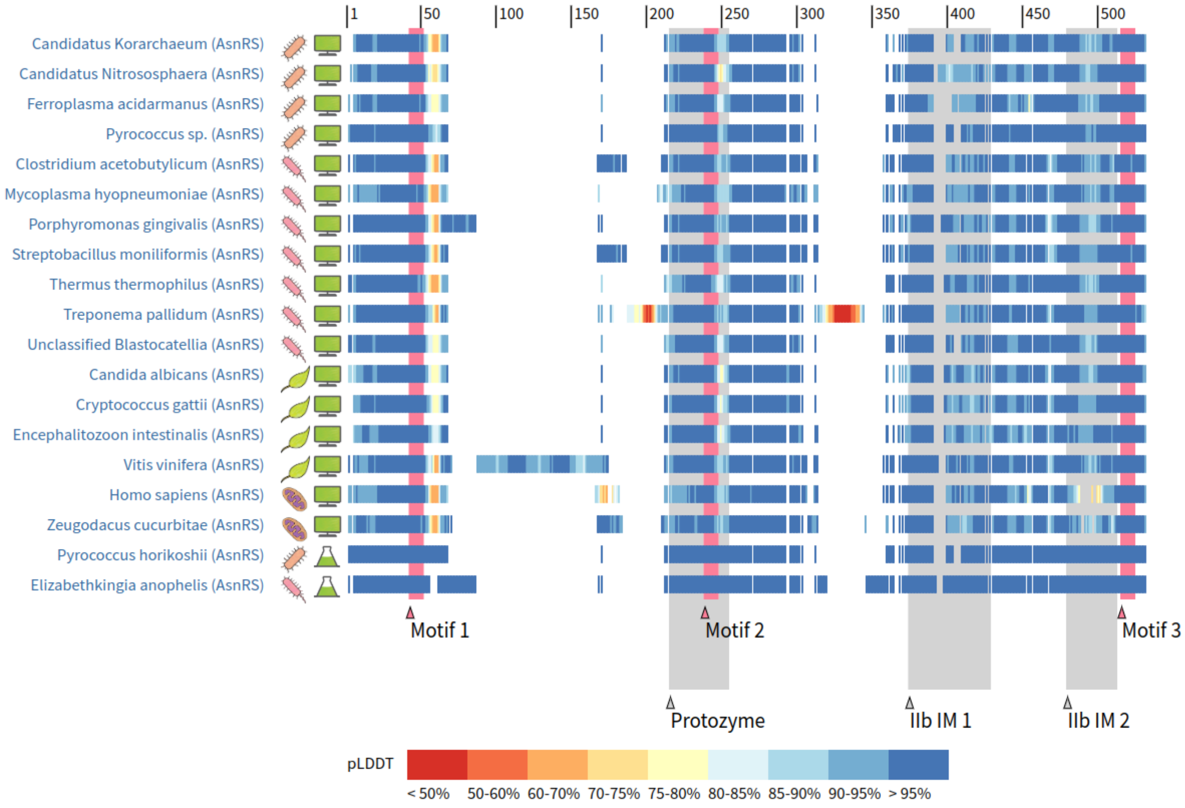


Fig. S19: **AsnRS** distribution of AlphaFold pLDDT scores for the catalytic domain. Regions of low confidence: the small interface loop downstream from motif 1. The median score was 96%, and 90% of all scores were greater than 89%. Refer to Fig. S4 for notation.

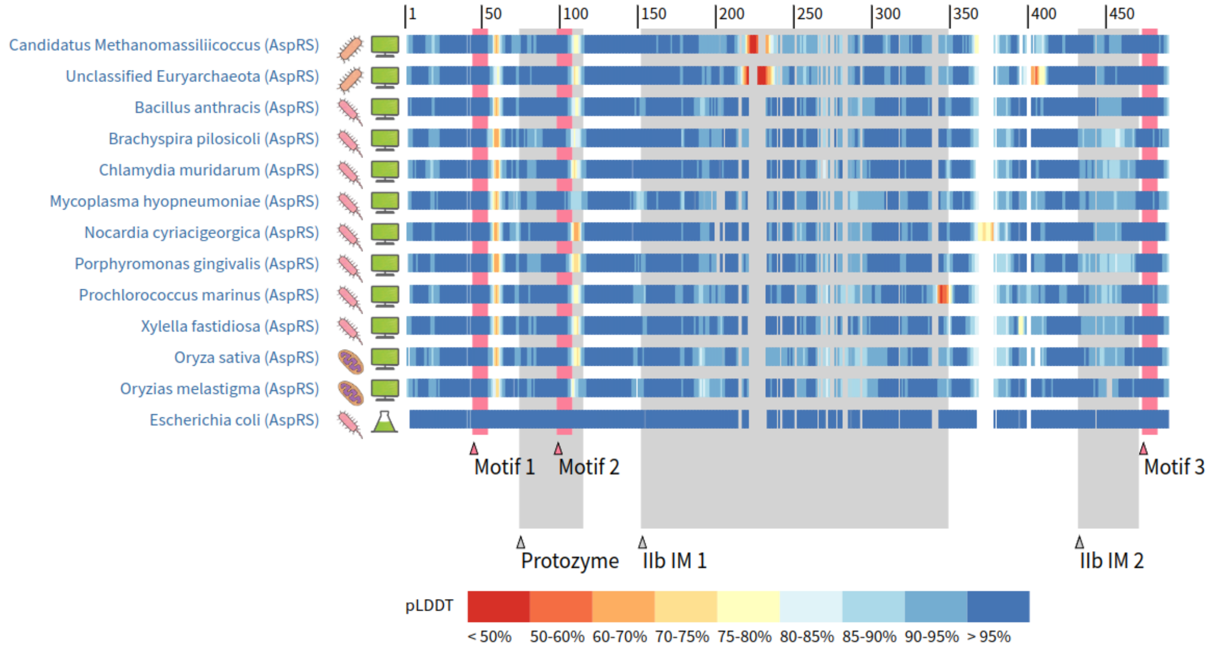


Fig. S20: **AspRS** distribution of AlphaFold pLDDT scores for the catalytic domain. Regions of low confidence: the small interface loop downstream from motif 1. The median score was 96%, and 90% of all scores were greater than 88%. Refer to Fig. S4 for notation.

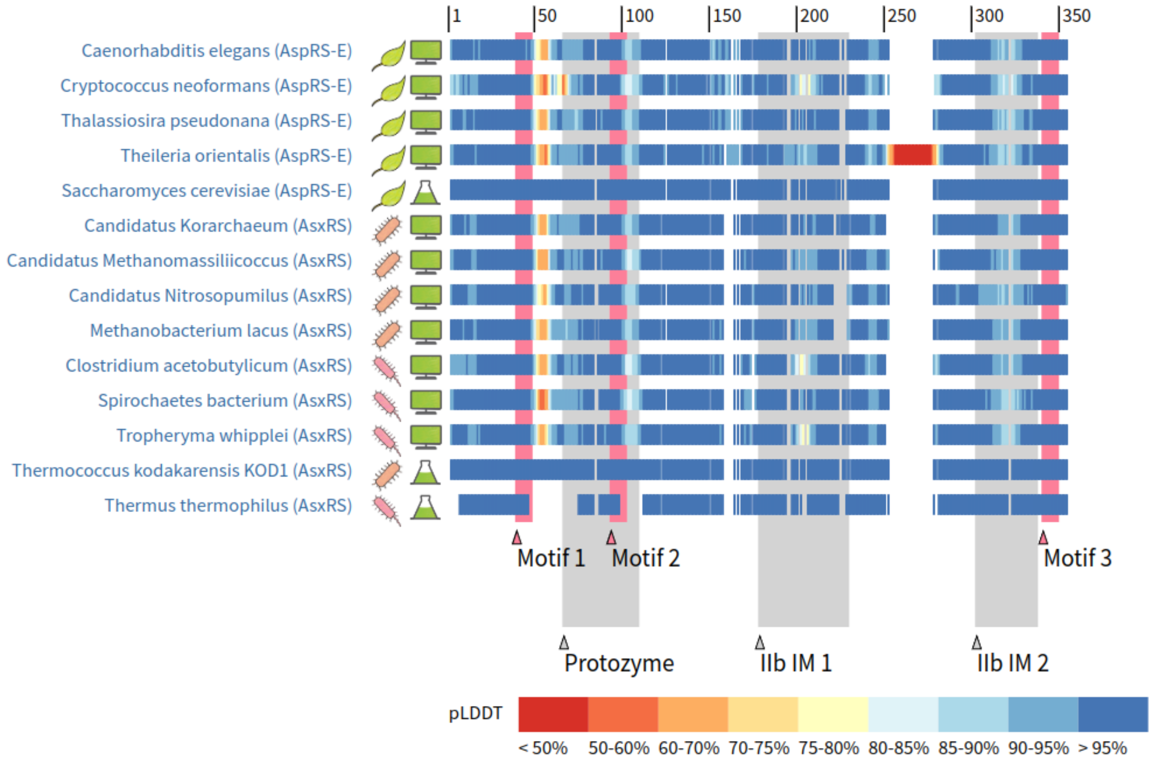


Fig. S21: **AspRS-E and AsxRS** distributions of AlphaFold pLDDT scores for the catalytic domain. Regions of low confidence: the small interface loop downstream from motif 1. The median score was 96%, and 90% of all scores were greater than 88%. Refer to Fig. S4 for notation.

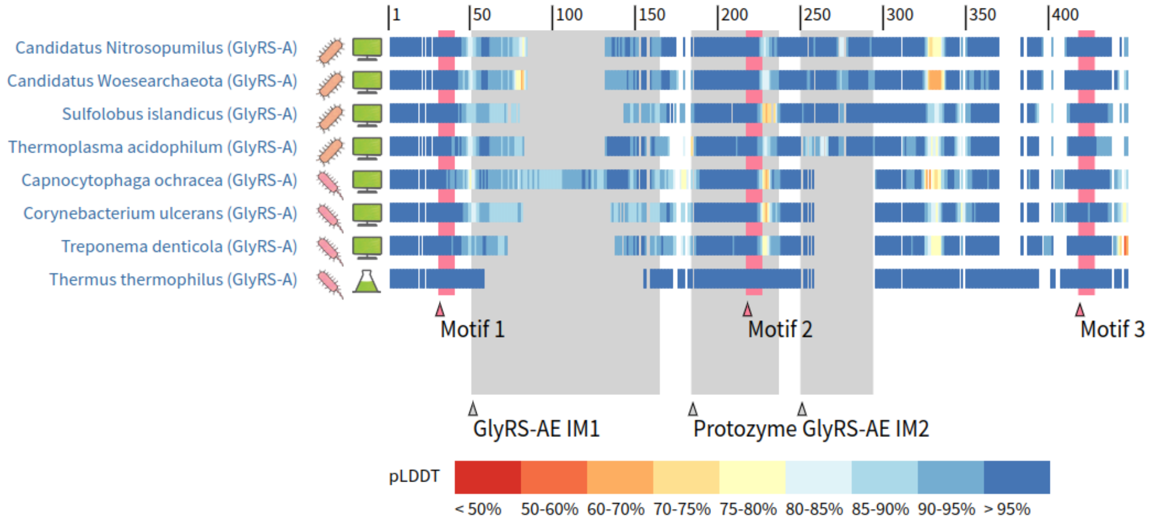


Fig. S22: **GlyRS-A** distribution of AlphaFold pLDDT scores for the catalytic domain. The median score was 96%, and 90% of all scores were greater than 88%. Refer to Fig. S4 for notation.

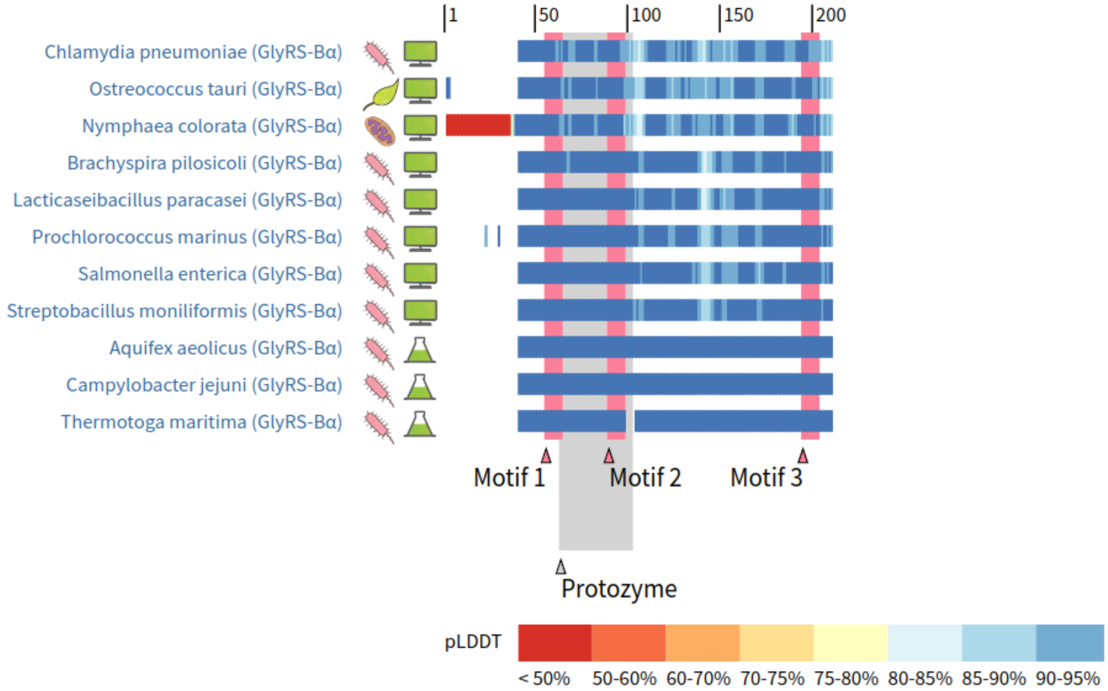


Fig. S23: **GlyRS-B** distribution of AlphaFold pLDDT scores for the catalytic domain. The median score was 97%, and 90% of all scores were greater than 84%. Refer to Fig. S4 for notation.

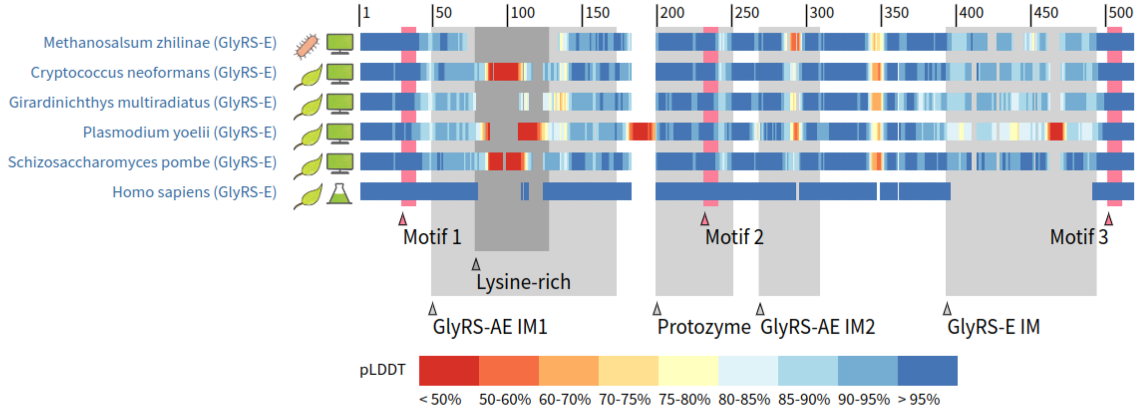


Fig. S24: **GlyRS-E** distribution of AlphaFold pLDDT scores for the catalytic domain. Regions of low confidence: a lysine-rich insertion within the GlyRS insertion nodule and a small loop upstream of the GlyRS-E insertion module. The median score was 94%, and 90% of all scores were greater than 62%. Refer to Fig. S4 for notation.

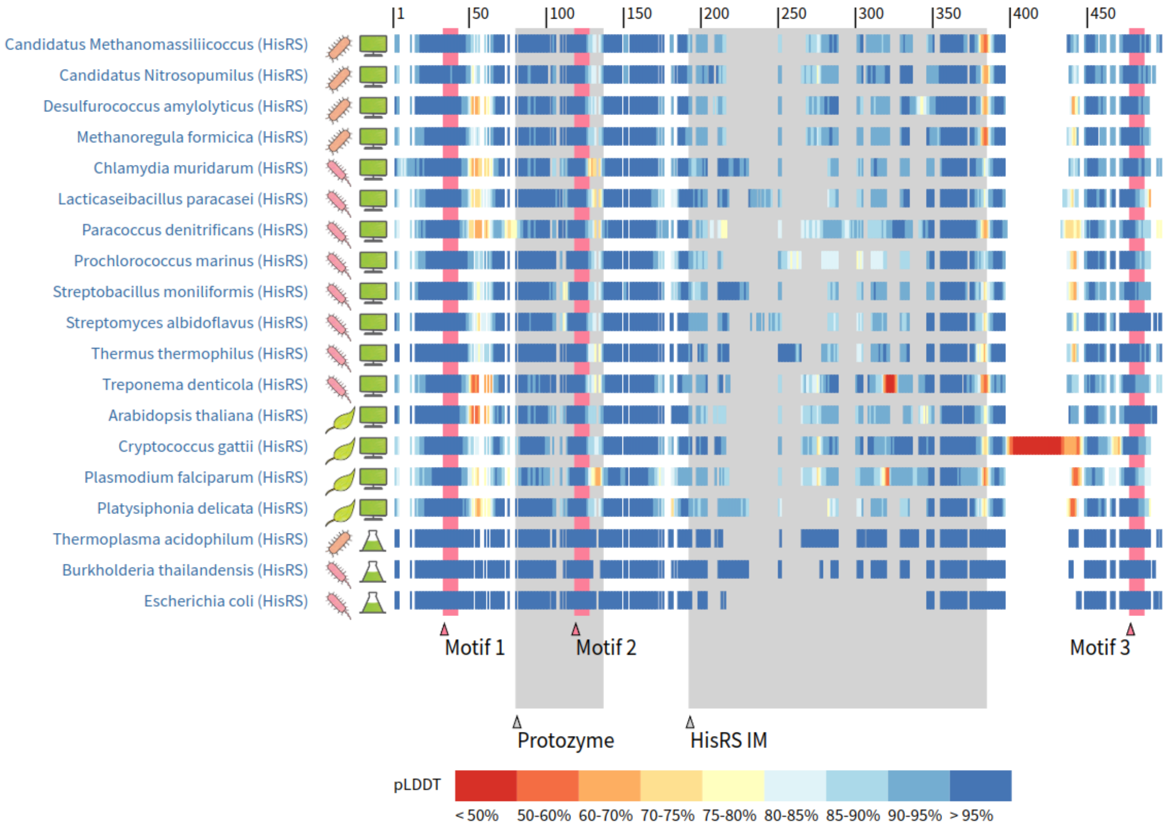


Fig. S25: **HisRS** distribution of AlphaFold pLDDT scores for the catalytic domain. Regions of low confidence: the small interface loop downstream from motif 1. The median score was 94%, and 90% of all scores were greater than 67%. Refer to Fig. S4 for notation.

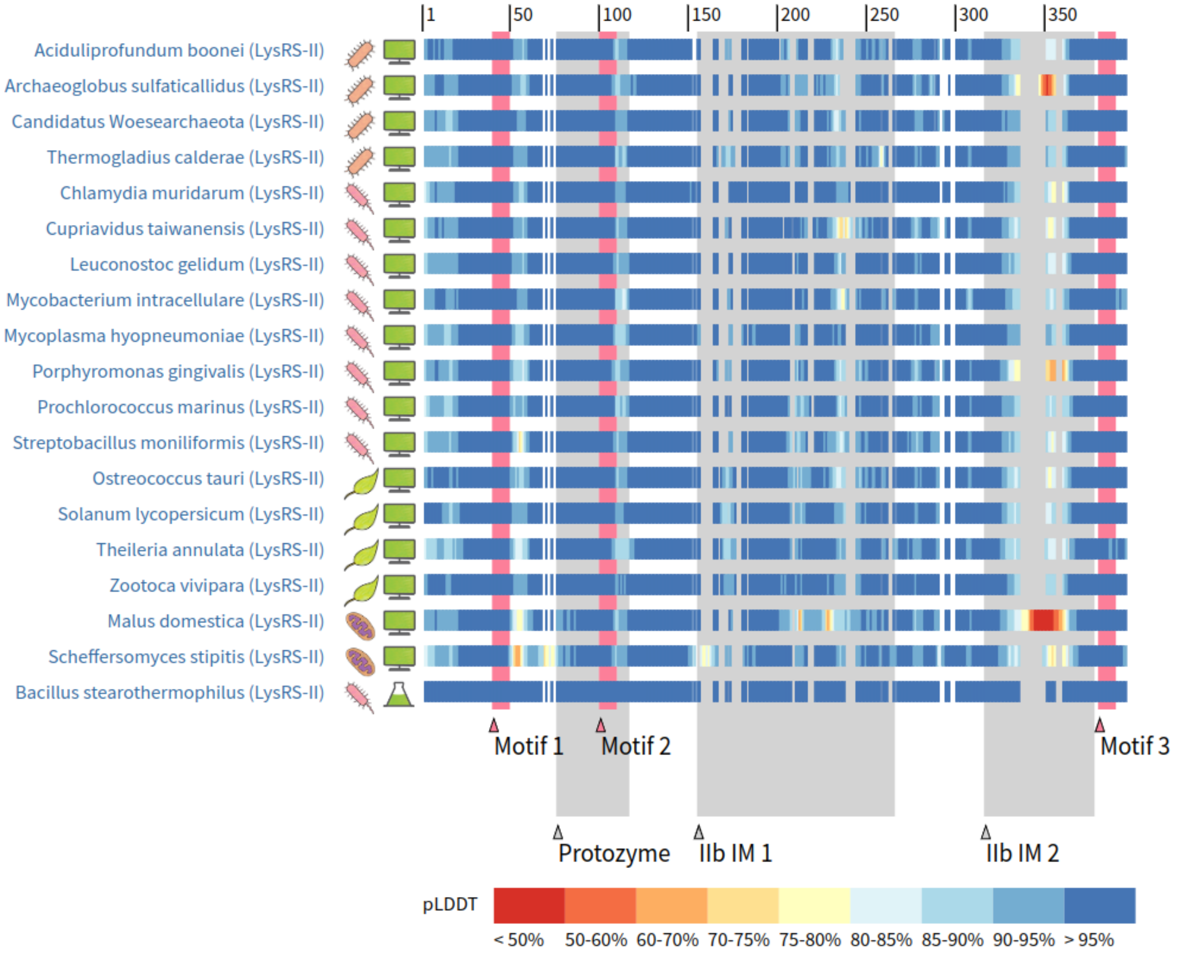


Fig. S26: **LysRS-II** distribution of AlphaFold pLDDT scores for the catalytic domain. The median score was 97%, and 90% of all scores were greater than 86%. Refer to Fig. S4 for notation.

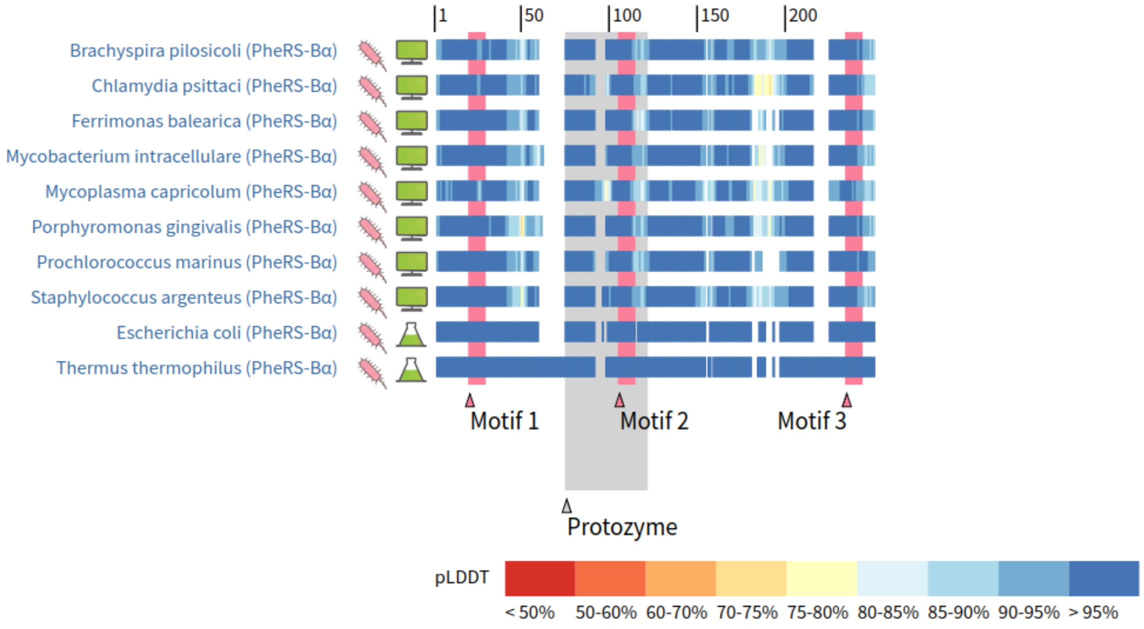


Fig. S27: **PheRS-B** $\alpha$ distribution of AlphaFold pLDDT scores for the catalytic domain. The median score was 95%, and 90% of all scores were greater than 86%. Refer to Fig. S4 for notation.

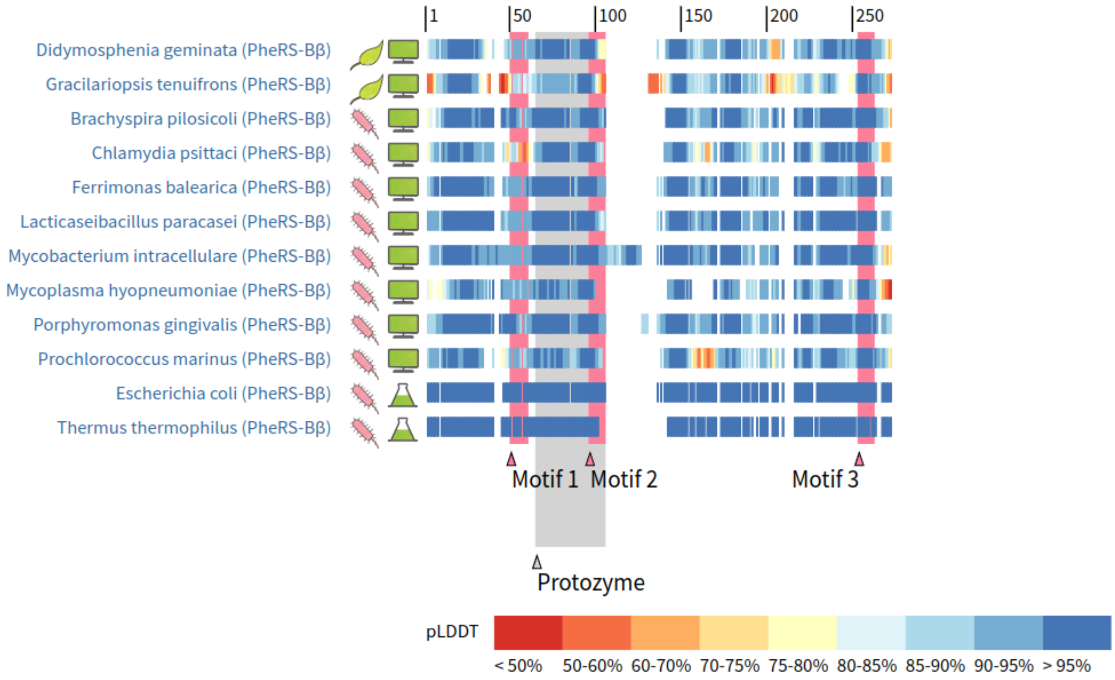


Fig. S28: **PheRS-B** $\beta$ distribution of AlphaFold pLDDT scores for the catalytic domain. The median score was 95%, and 90% of all scores were greater than 86%. Refer to Fig. S4 for notation.

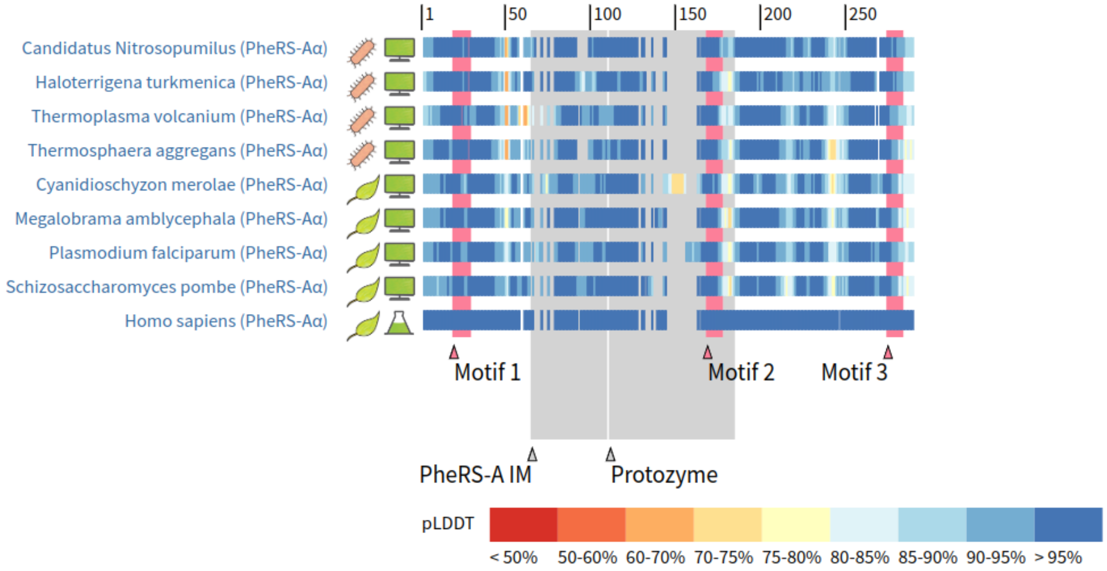


Fig. S29: **PheRS-A** $\alpha$ distribution of AlphaFold pLDDT scores for the catalytic domain. The median score was 91%, and 90% of all scores were greater than 80%. Refer to Fig. S4 for notation.

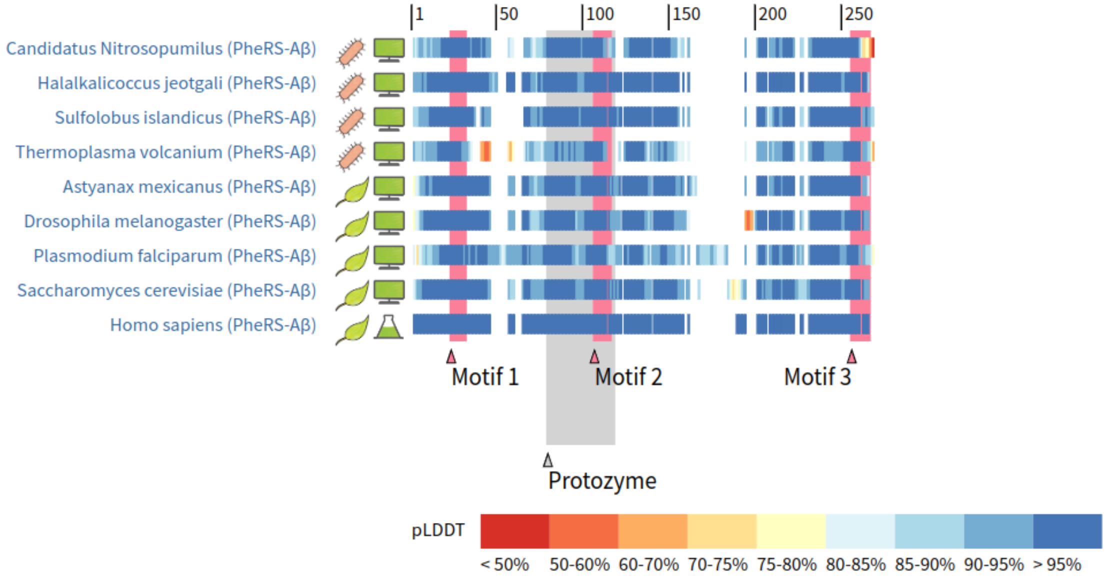


Fig. S30: **PheRS-A** $\beta$ distribution of AlphaFold pLDDT scores for the catalytic domain. The median score was 95%, and 90% of all scores were greater than 89%. Refer to Fig. S4 for notation.

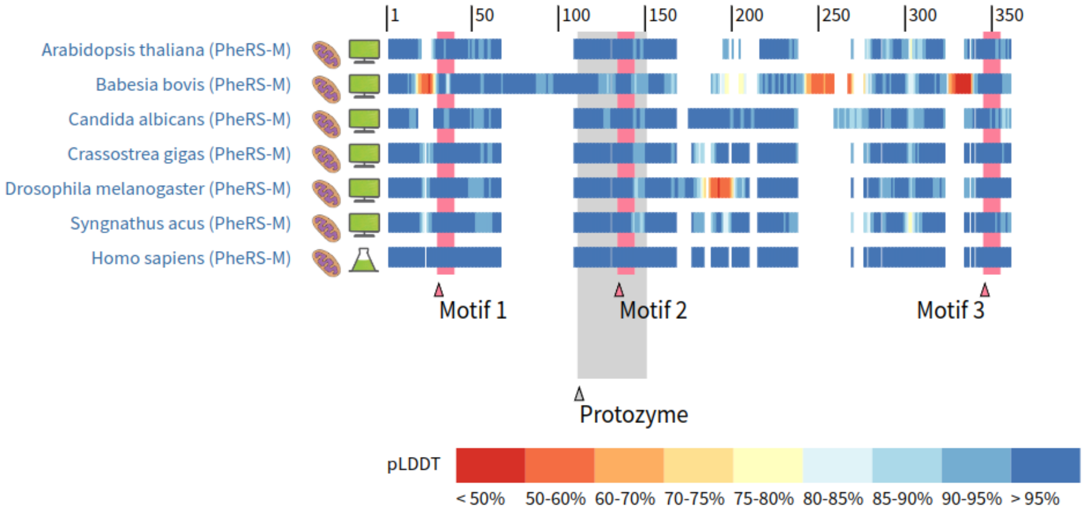


Fig. S31: **PheRS-M** distribution of AlphaFold pLDDT scores for the catalytic domain. The median score was 96%, and 90% of all scores were greater than 82%. Refer to Fig. S4 for notation.

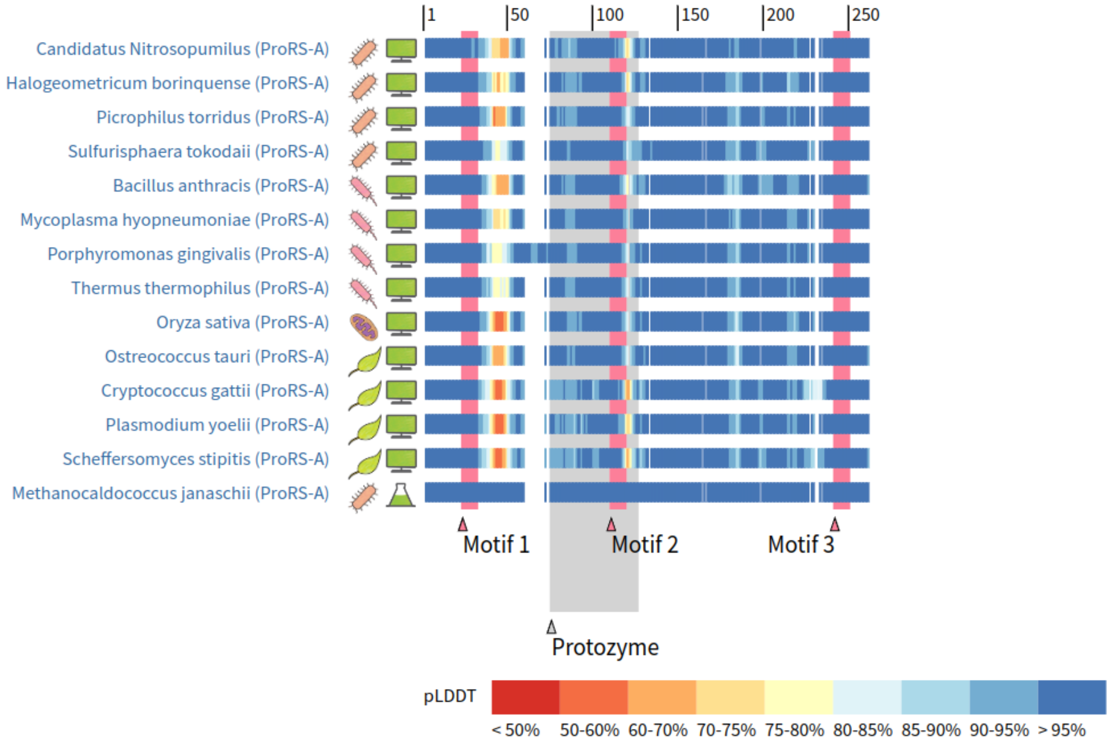


Fig. S32: **ProRS-A** distribution of AlphaFold pLDDT scores for the catalytic domain. Regions of low confidence: the small interface loop downstream from motif 1. The median score was 97%, and 90% of all scores were greater than 82%. Refer to Fig. S4 for notation.

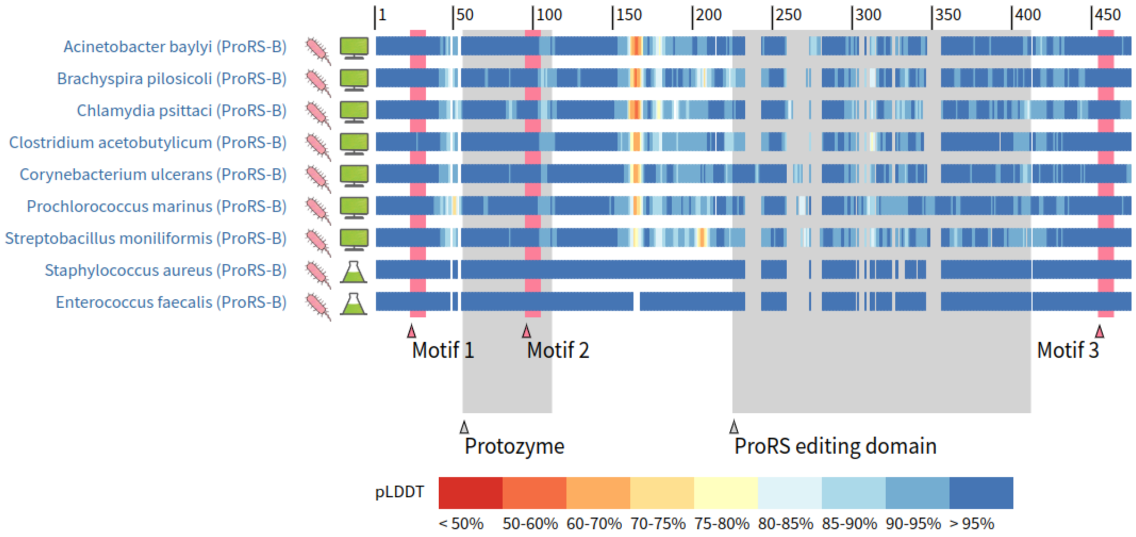


Fig. S33: **ProRS-B** distribution of AlphaFold pLDDT scores for the catalytic domain. Regions of low confidence: a short hairpin loop downstream of motif 2. The median score was 96%, and 90% of all scores were greater than 90%. Refer to Fig. S4 for notation.

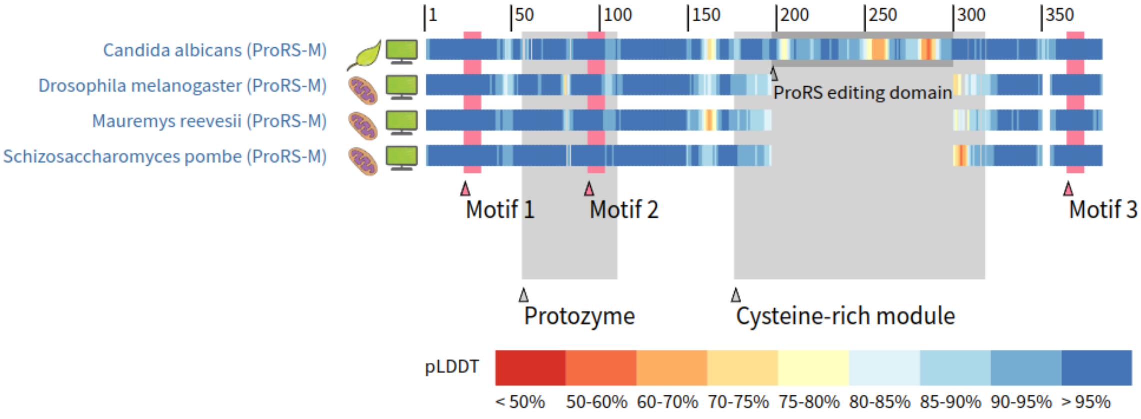


Fig. S34: **ProRS-M** distribution of AlphaFold pLDDT scores for the catalytic domain. Regions of low confidence: parts of the editing domain and cysteine-rich module. The median score was 96%, and 90% of all scores were greater than 36%. Refer to Fig. S4 for notation.

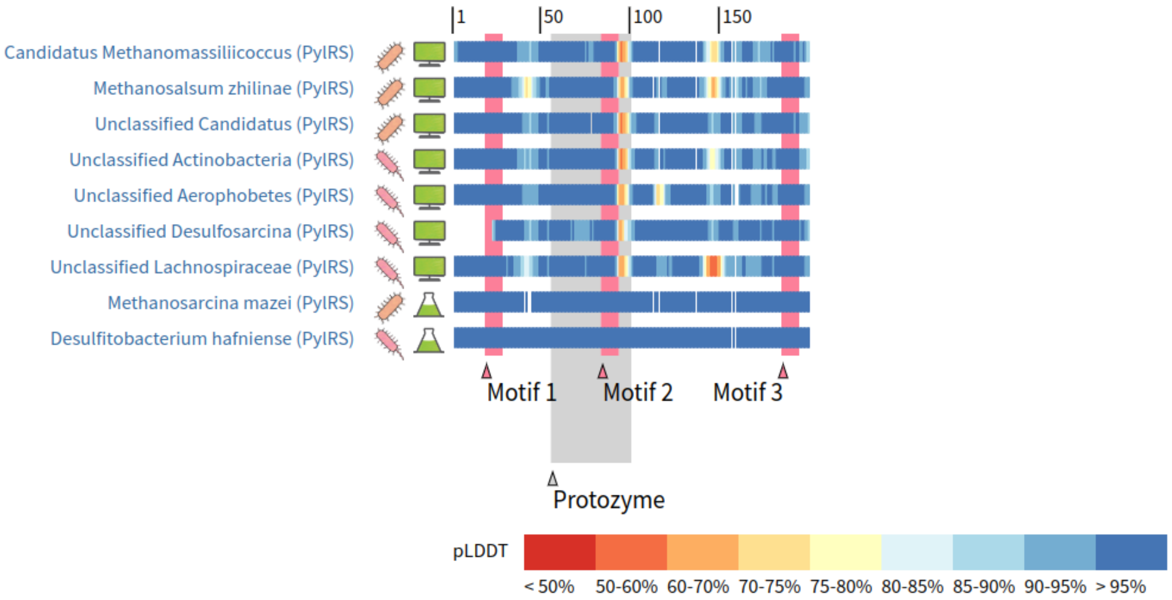


Fig. S35: **PylRS** distribution of AlphaFold pLDDT scores for the catalytic domain. Regions of low confidence: a short hairpin loop downstream of motif 2. The median score was 96%, and 90% of all scores were greater than 87%. Refer to Fig. S4 for notation.

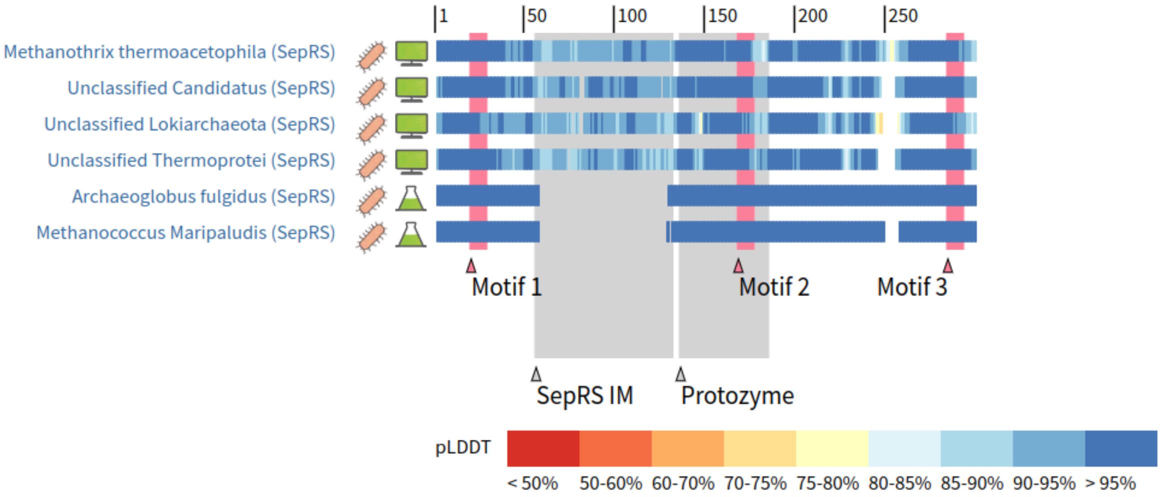


Fig. S36: **SepRS** distribution of AlphaFold pLDDT scores for the catalytic domain. The median score was 95%, and 90% of all scores were greater than 88%. Refer to Fig. S4 for notation.

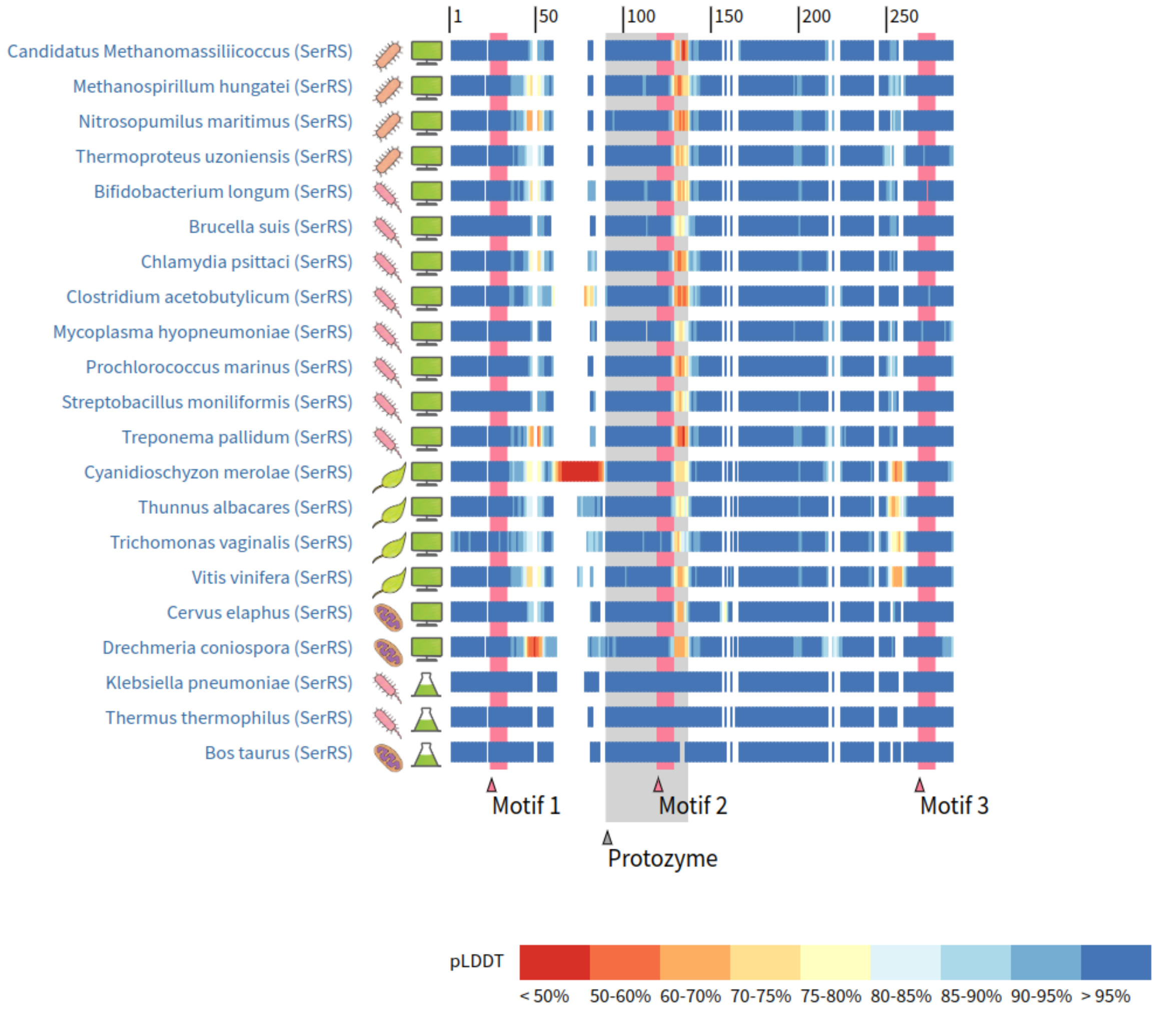


Fig. S37: **SerRS** distribution of AlphaFold pLDDT scores for the catalytic domain. Regions of low confidence: a short loop downstream of motif 2. The median score was 97%, and 90% of all scores were greater than 88%. Refer to Fig. S4 for notation.

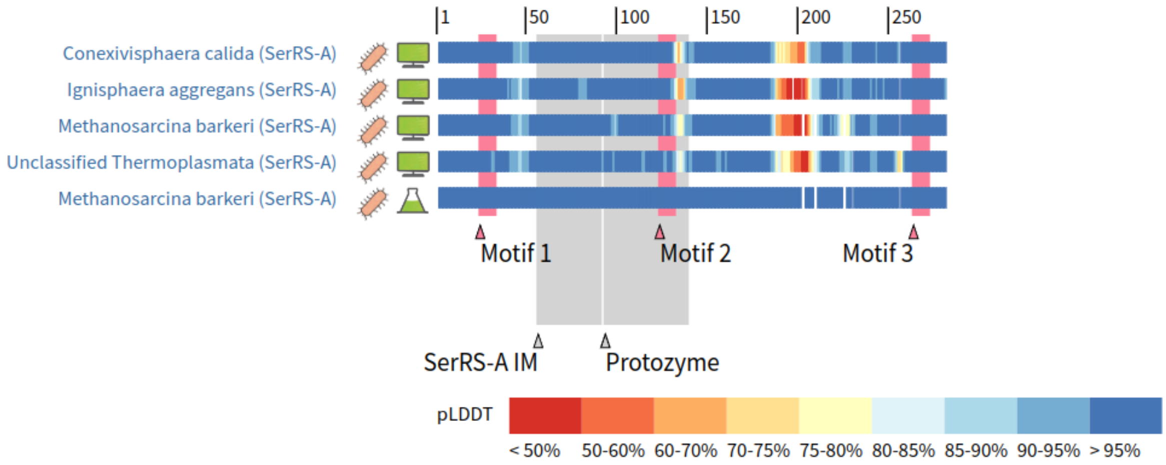


Fig. S38: **SerRS-A** distribution of AlphaFold pLDDT scores for the catalytic domain. Regions of low confidence: a short loop on the surface of the catalytic domain between motifs 2 and 3. The median score was 96%, and 90% of all scores were greater than 86%. Refer to Fig. S4 for notation.

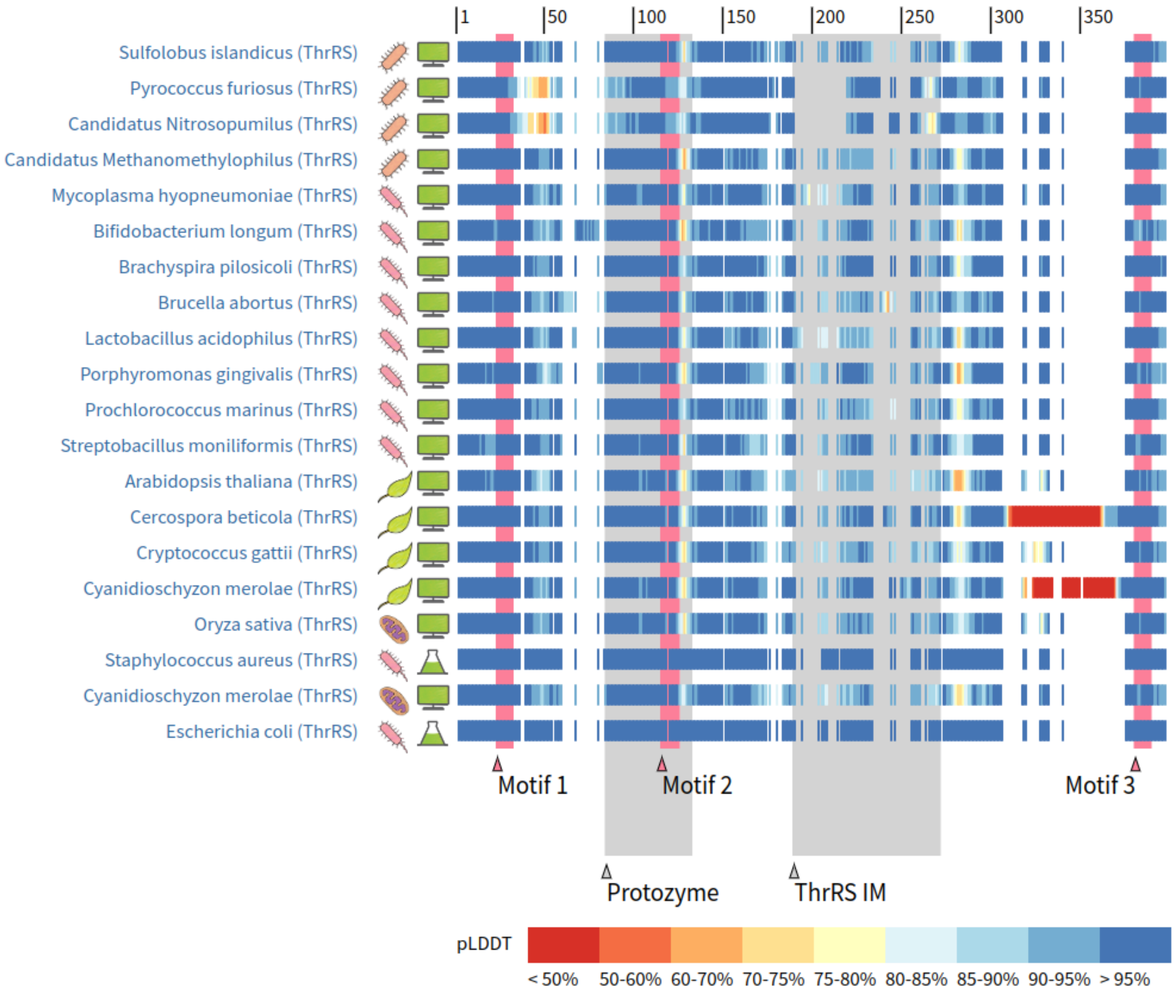


Fig. S39: **ThrRS** distribution of AlphaFold pLDDT scores for the catalytic domain. The median score was 96%, and 90% of all scores were greater than 87%. Refer to Fig. S4 for notation.

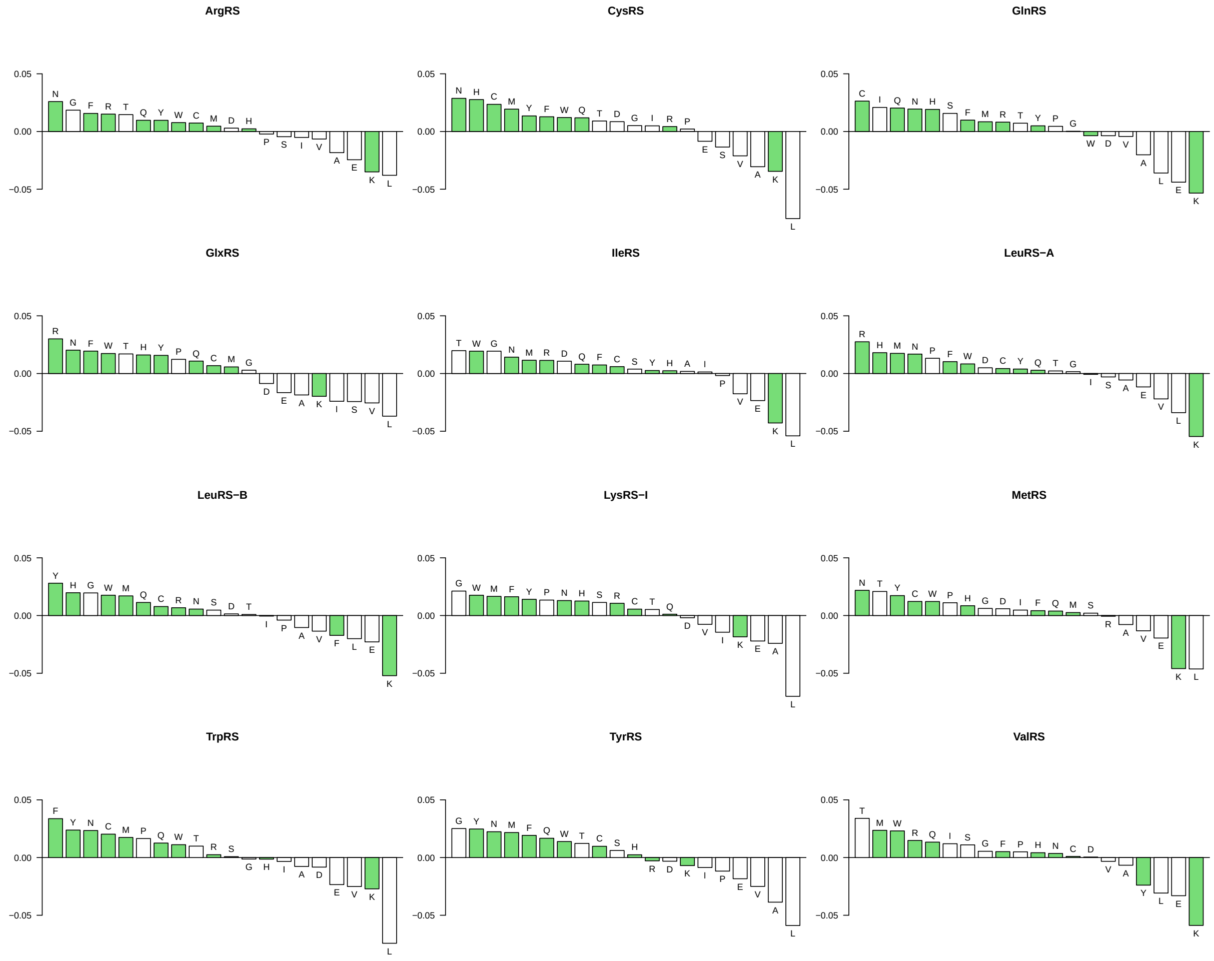


Fig. S40: **Class I**: increase in amino acid frequencies since the most recent common ancestor of each family. Green bars are Phase II amino acids according to Wong. The varying GluRS families were was joined into a single clade.

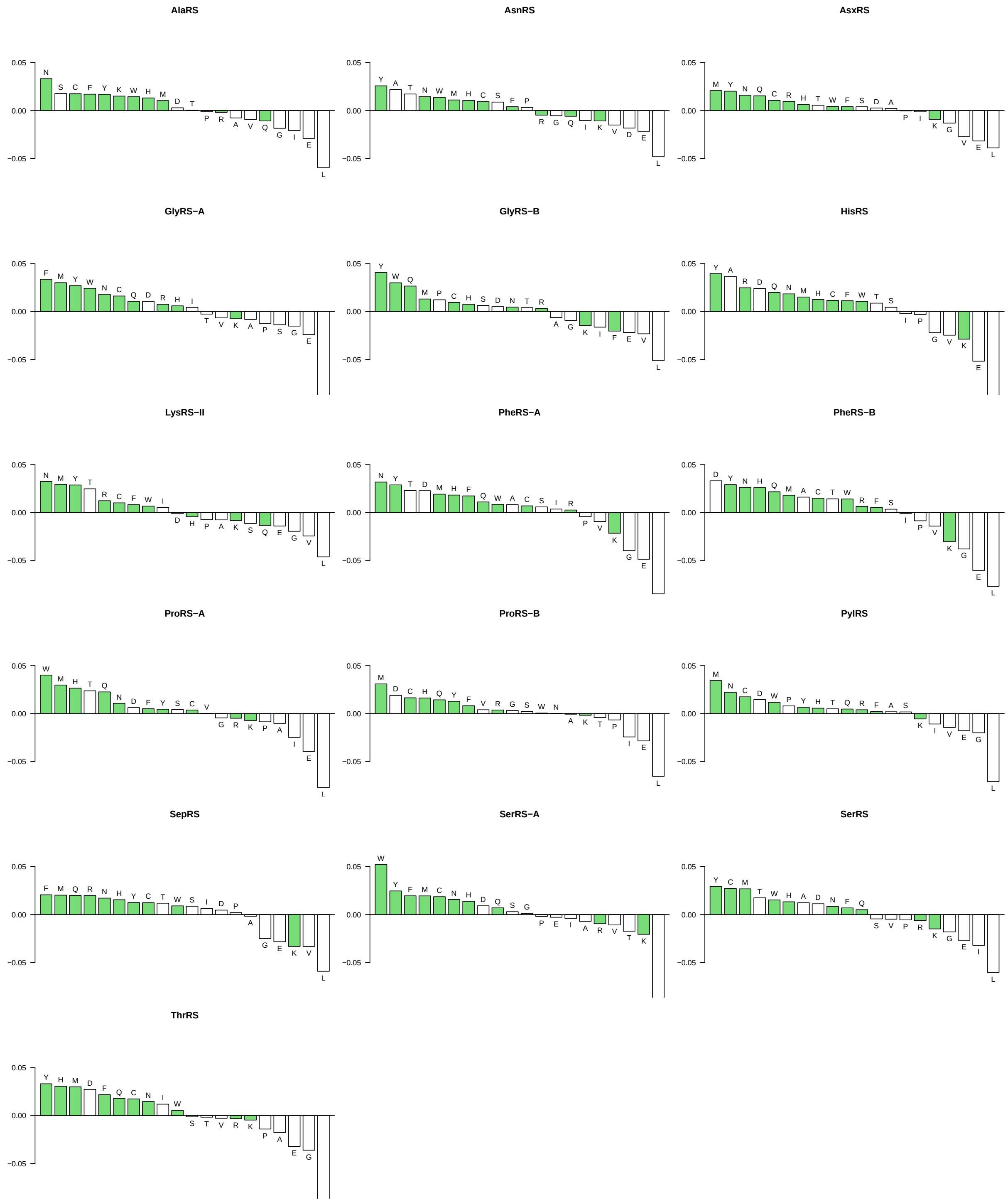


Fig. S41: **Class II**: increase in amino acid frequencies since the most recent common ancestor of each family. Green bars are Phase II amino acids according to Wong. ProRS-B and ProRS-M were was joined into a single clade, as were PheRS-B/PheRS-M, and AspRS/AspRS-E/AsxRS.

# Class I Insertion Module Phylogenies

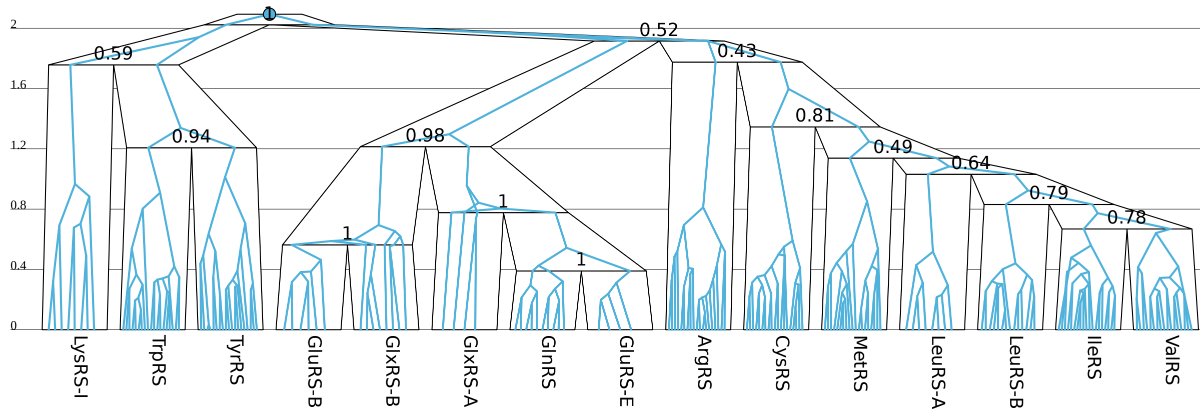


Fig. S42: **Class I protozyme, urzyme-minus-protozyme, and CP1 phylogenies**. Family tree internal nodes are labelled by posterior support. All of the subsequent Class I modules are assumed to have a phylogeny which is a clade of the coloured phylogeny above. These modules have a mean (and 95% credible interval) amino acid substitution rate of - protozyme: 0.638 (0.586,0.683); urzyme: 0.623 (0.565,0.678); and CP1: 0.763 (0.715,0.809).

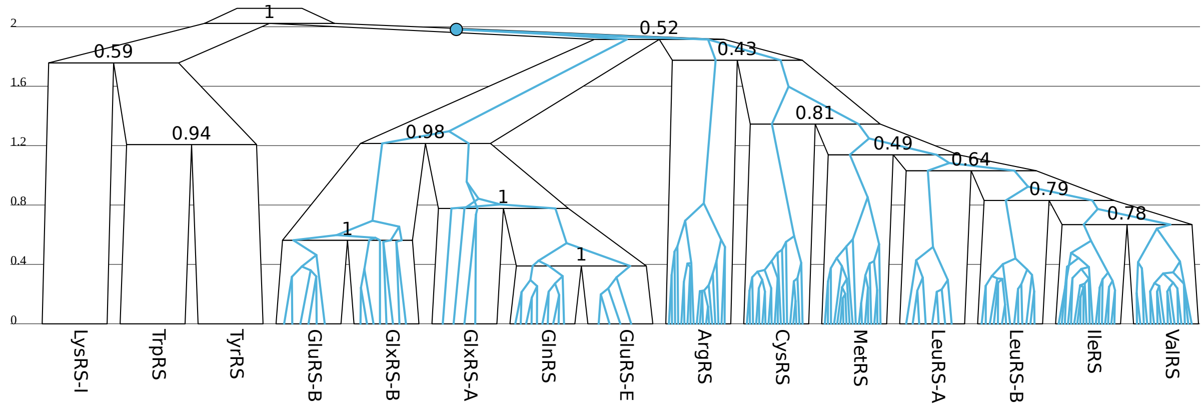


Fig. S43: **Z insertion module phylogeny.** The root of the tree is the last common ancestor of Z after its origin. Estimated substitution rate: 0.745 (0.675,0.84).

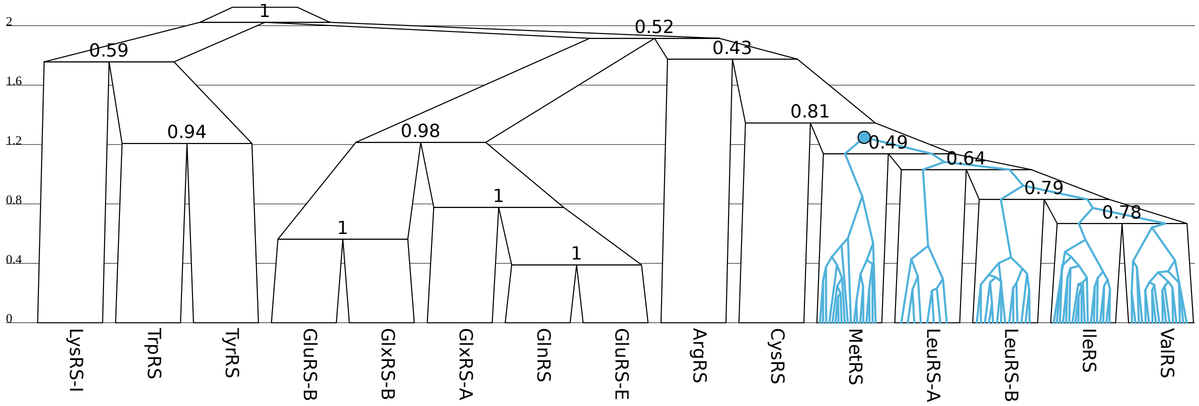


Fig. S44: **CP2 and the zinc finger phylogeny**. Estimated substitution rates - CP2: 1.17 (0.822,1.39); zinc finger: 0.841 (0.708,0.976).

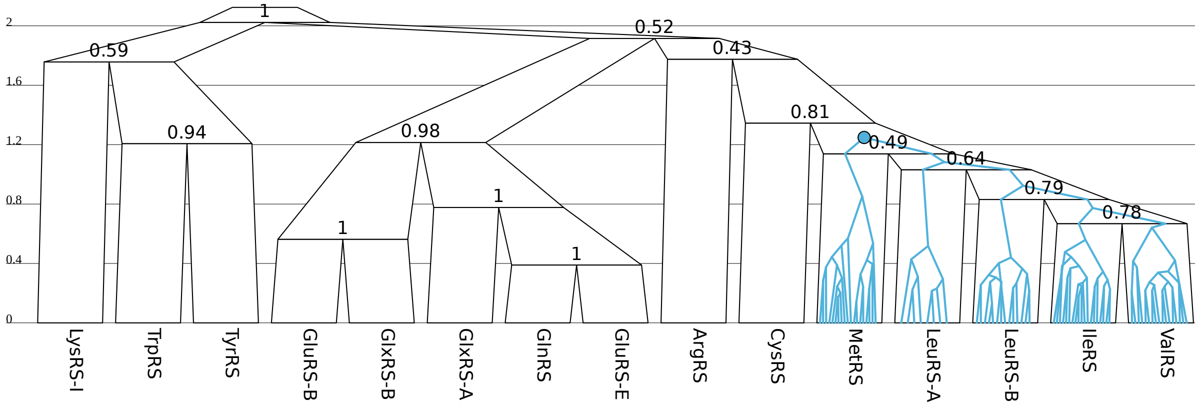


Fig. S45: **Subclass Ia editing domain phylogeny.** Estimated substitution rate: 1.04 (0.993,1.09).

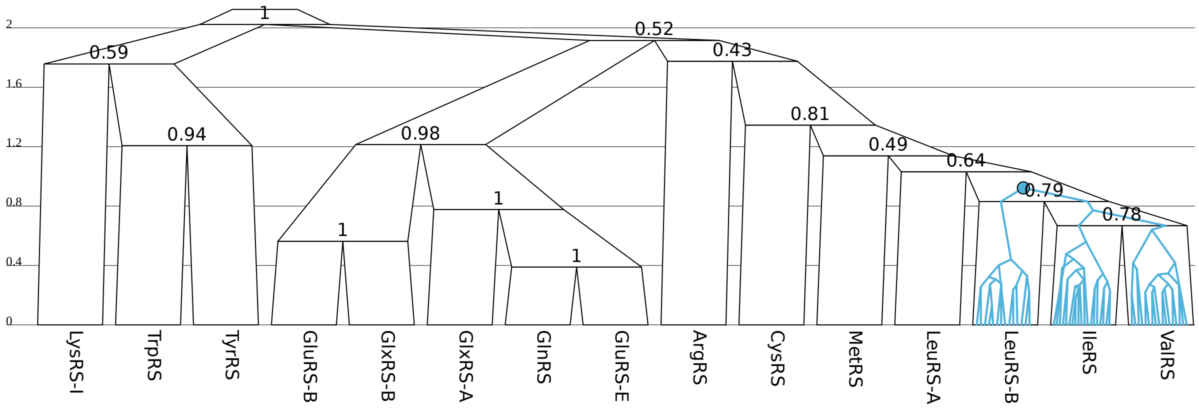


Fig. S46: **CP3 phylogeny**. Estimated substitution rate: 1.23 (1.08,1.36).

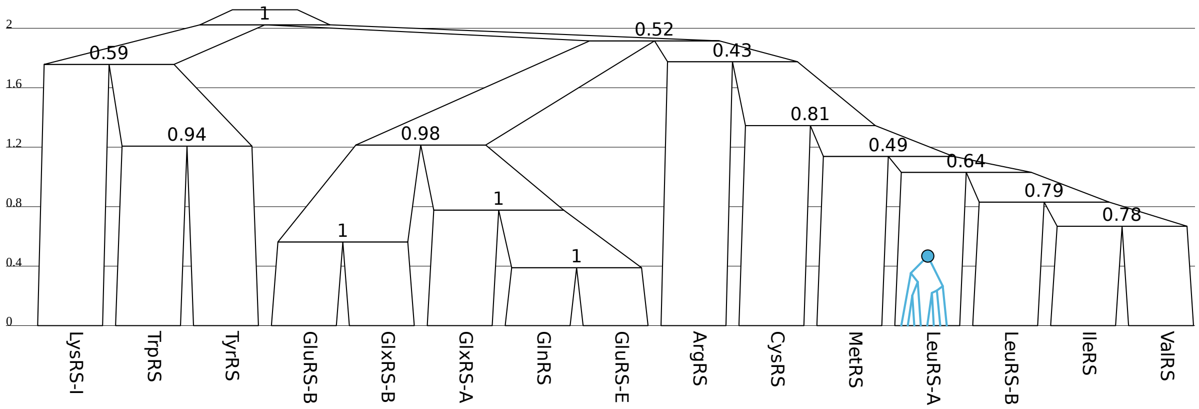


Fig. S47: **LeuRS A insertion module phylogenies**. Estimated substitution rates- LeuRS-A IM1: 1.19 (0.946,1.46); LeuRS-A IM2: 0.861 (0.686,1.05).

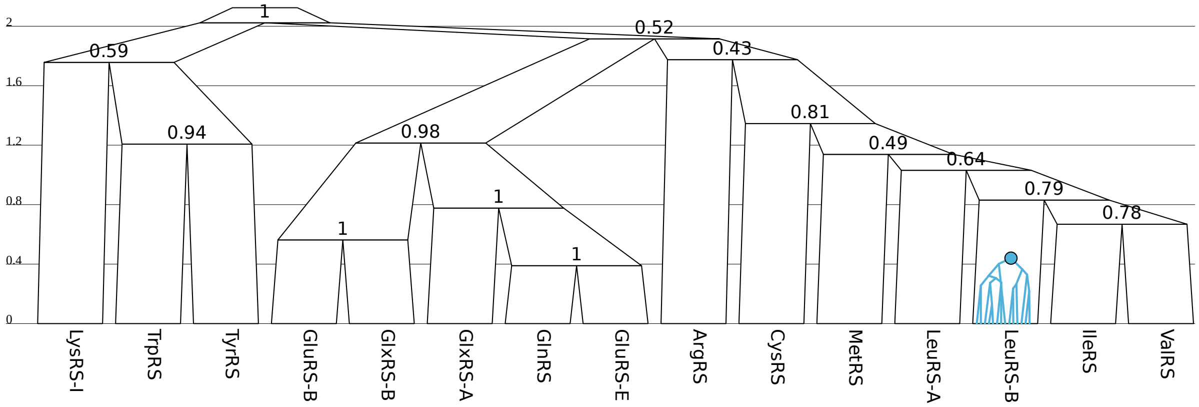


Fig. S48: **LeuRS insertion module phylogenies**. Estimated substitution rate: 1.74 (1.43,1.99).

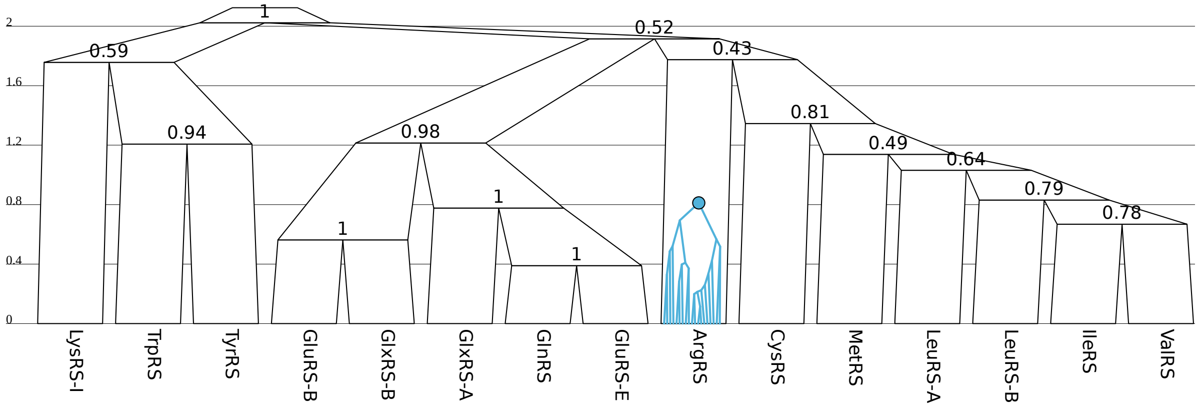


Fig. S49: **ArgRS insertion module phylogeny**. Estimated substitution rate: 0.94 (0.816,1.09).

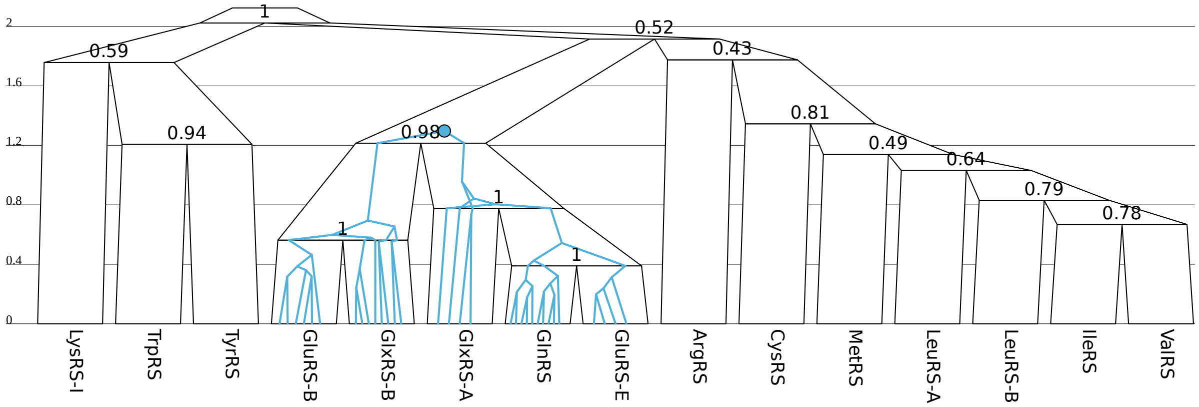


Fig. S50: **Ib insertion module phylogeny**. Estimated substitution rate: 1.1 (0.923,1.3).

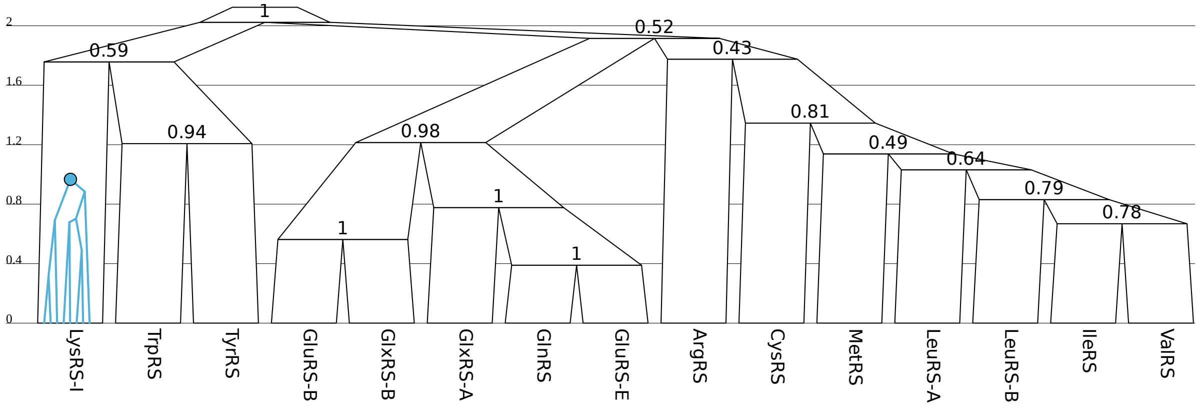


Fig. S51: **LysRS-I insertion module phylogeny**. Estimated substitution rate: 0.785 (0.617,0.954).

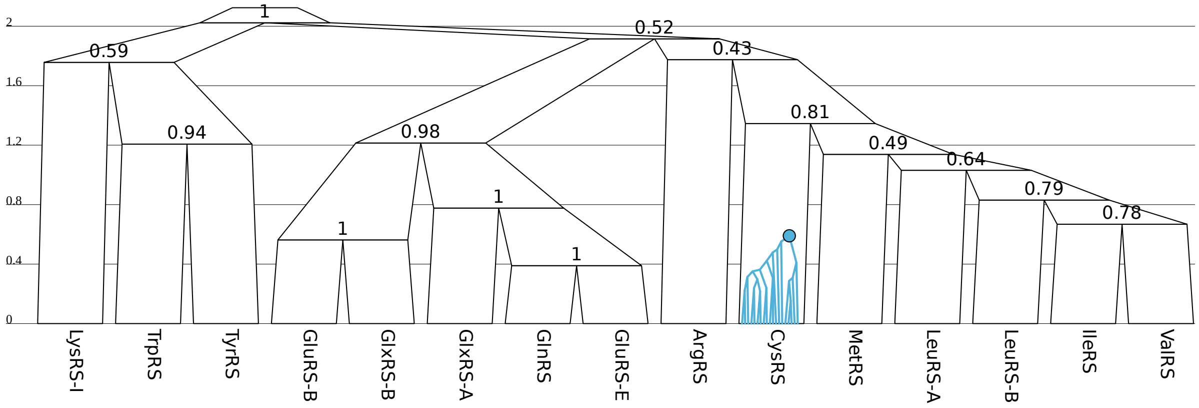


Fig. S52: **CysRS insertion module phylogeny**. Estimated substitution rate: 1.13 (0.899,1.34).

# Class II Insertion Module Phylogenies

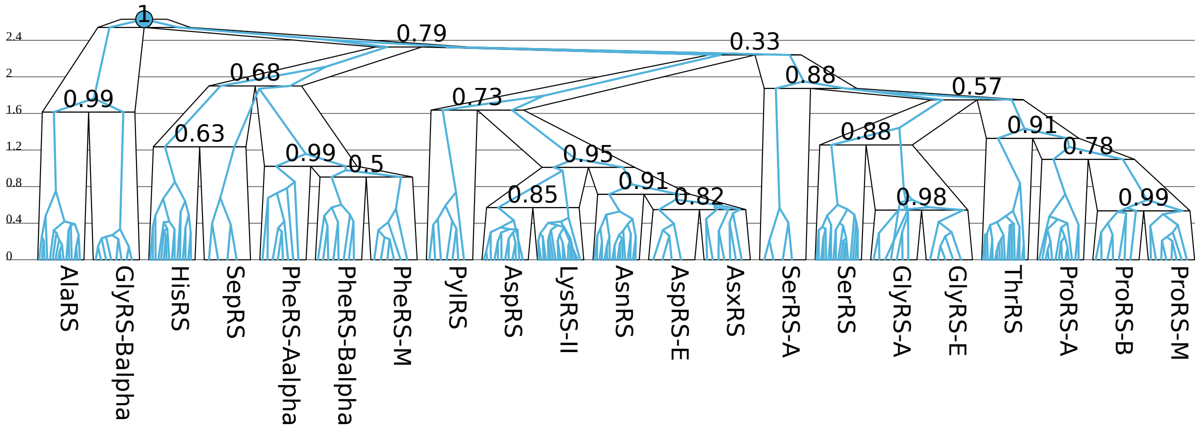


Fig. S53: **Class II protozyme, urzyme-minus-protozyme, and 6-fold-minus-urzyme phylogenies**. Family tree internal nodes are labelled by posterior support. All of the subsequent Class II modules are assumed to have a phylogeny which is a clade of the coloured phylogeny above. These modules have a mean (and 95% credible interval) relative amino acid substitution rate of - protozyme: 0.489 (0.452,0.532); urzyme: 0.744 (0.684,0.802); 6-fold: 0.516 (0.468,0.558).

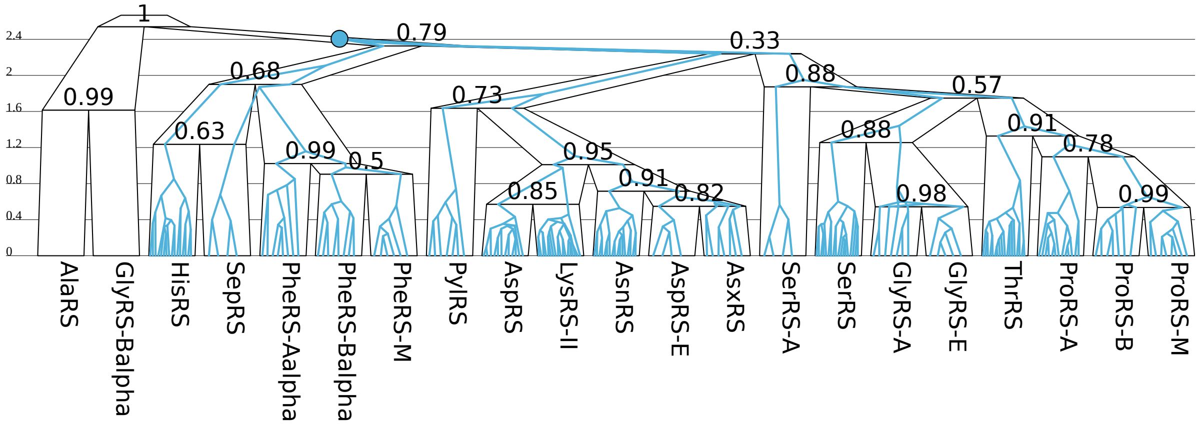


Fig. S54: **Small interface phylogeny**. Estimated substitution rate: 1.03 (0.688,1.19)

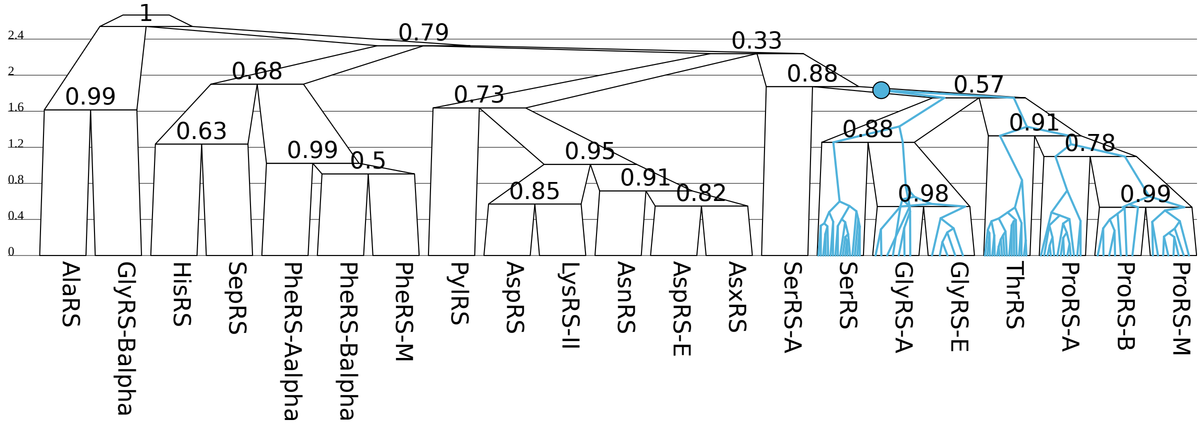


Fig. S55: **IIa insertion module phylogeny**. Estimated substitution rate: 1.01 (0.746,1.31).

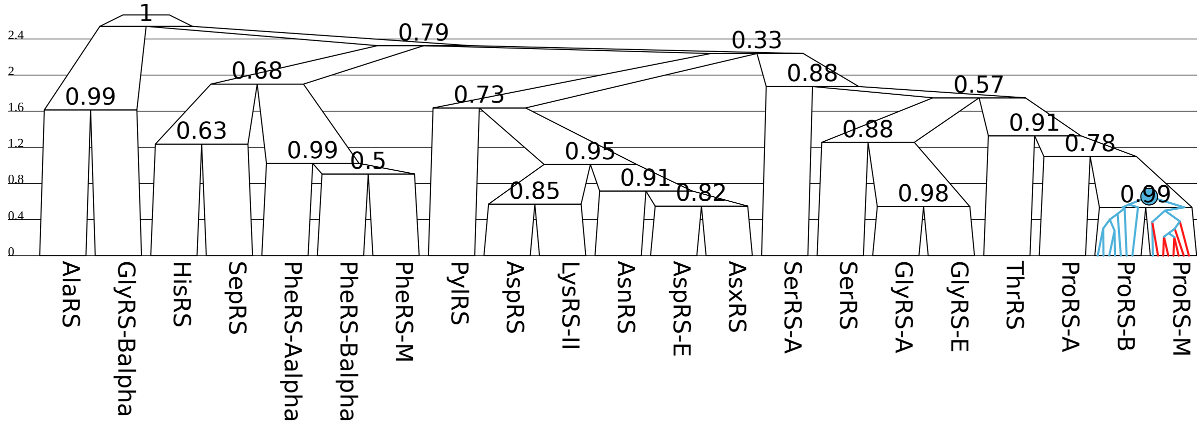


Fig. S56: **ProRS editing domain phylogeny**. The lineages in red are lacking the insertion module, due to deletion event(s). Estimated substitution rate: 1.28 (1.09,1.49).

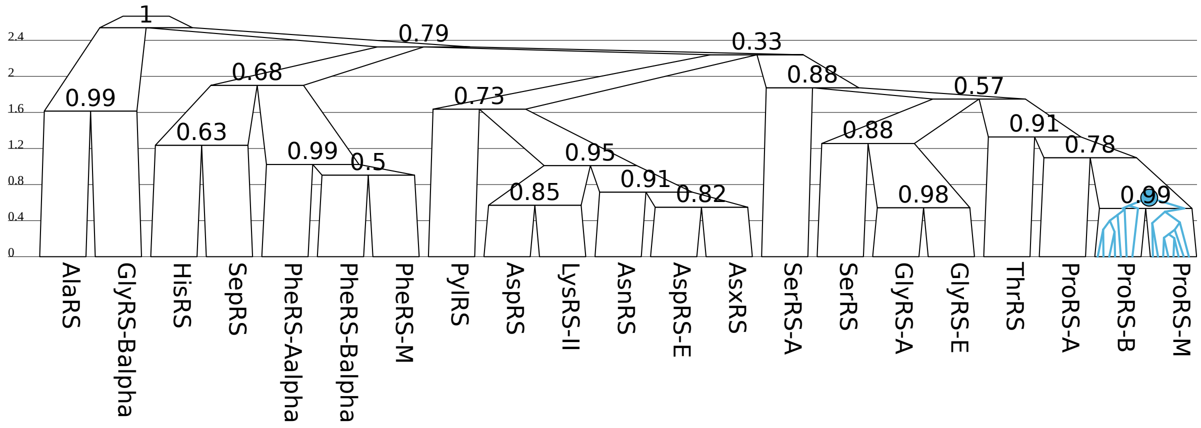


Fig. S57: **ProRS insertion module phylogeny**. Estimated substitution rate: 1.1 (0.882,1.33).

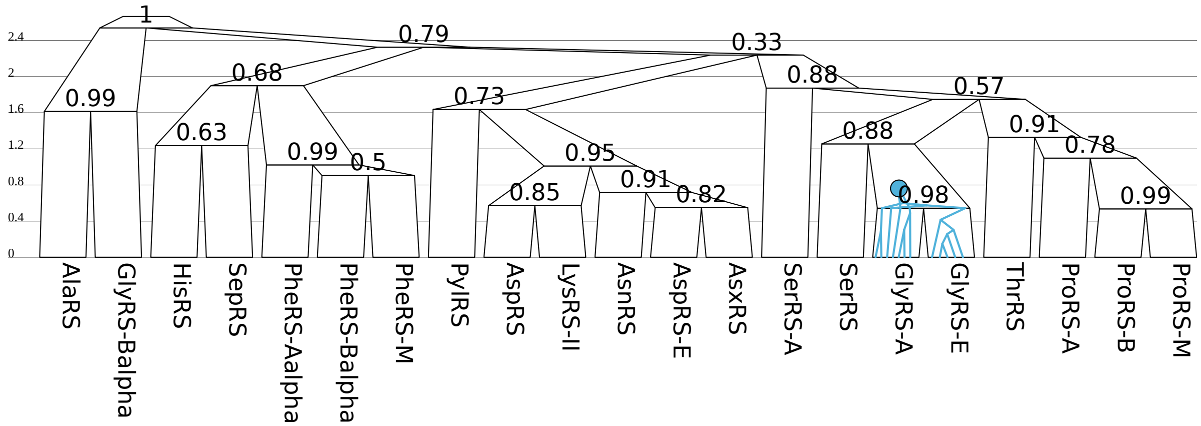


Fig. S58: **GlyRS insertion module phylogenies**. Estimated substitution rates - GlyRS IM1: 1.03 (0.843,1.2); GlyRS IM2: 1.19 (0.893,1.53).

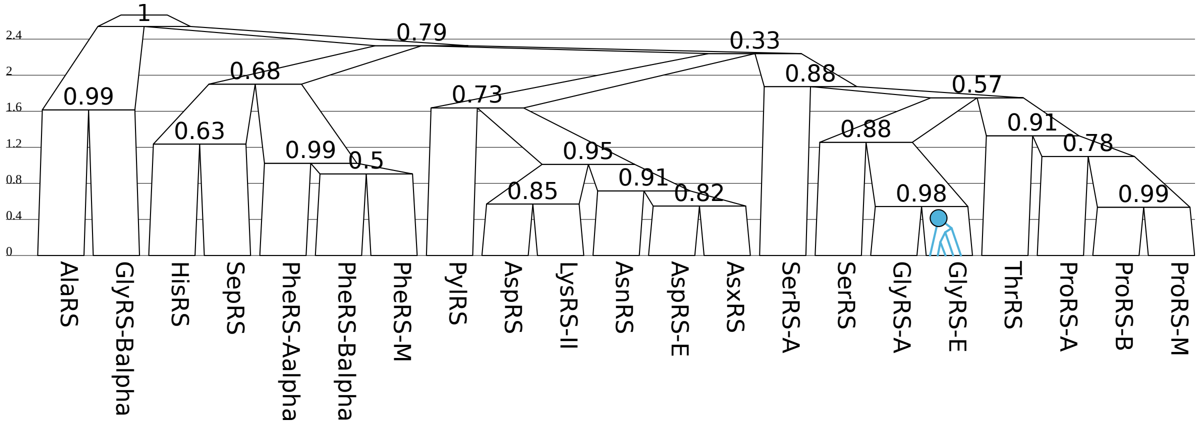


Fig. S59: **GlyRS-E insertion module phylogeny**. Estimated substitution rate: 1.52 (1.23,1.89).

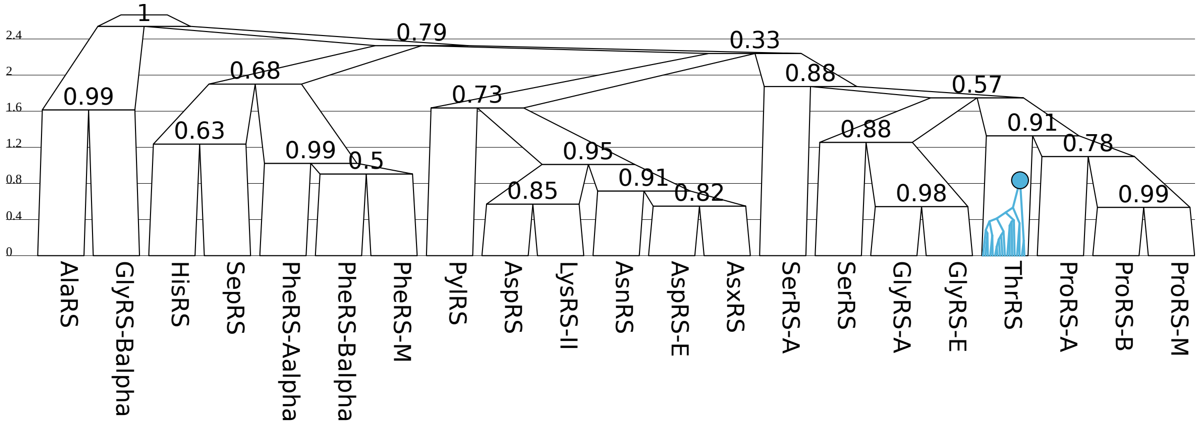


Fig. S60: **ThrRS insertion module phylogeny**. Estimated substitution rate: 0.852 (0.636,1.05).

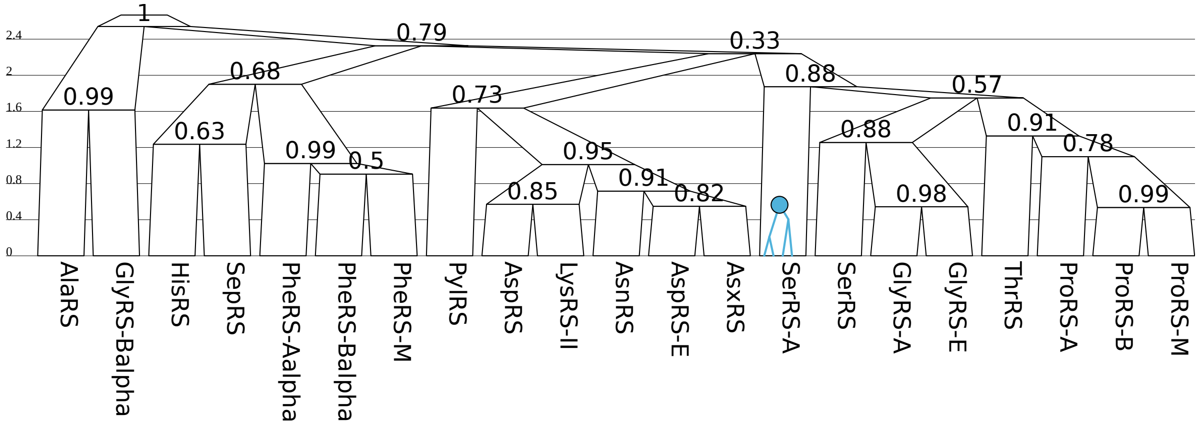


Fig. S61: **SerRS-A insertion module phylogeny**. Estimated substitution rate: 0.984 (0.706,1.42).

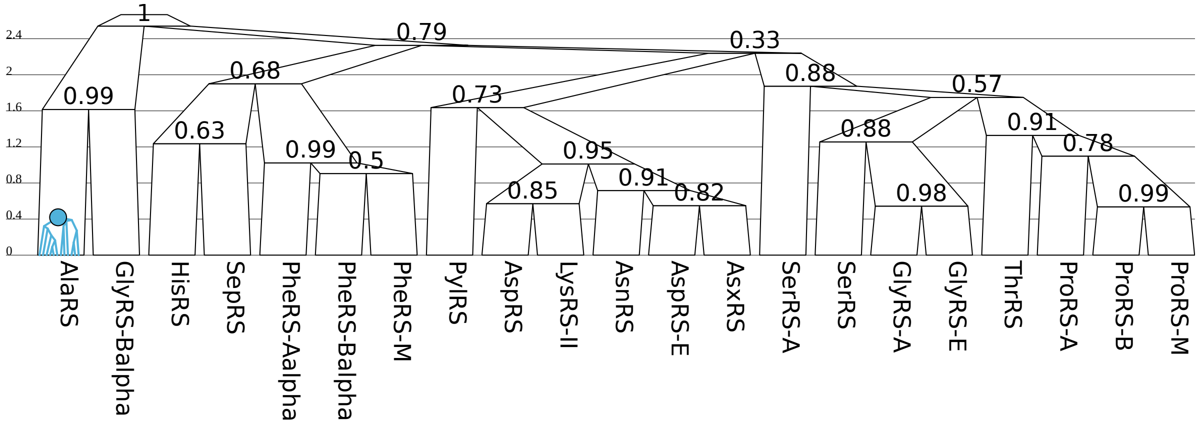


Fig. S62: **AlaRS insertion module phylogeny**. Estimated substitution rate: 0.992 (0.732,1.23).

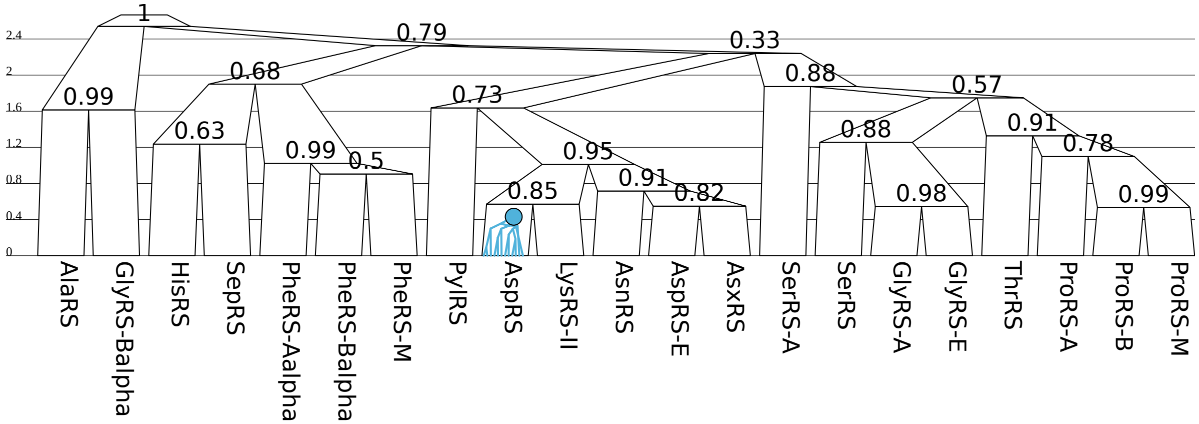


Fig. S63: **AspRS insertion module phylogenies**. Estimated substitution rates - AspRS IM1: 1.2 (0.988,1.44); AspRS IM2: 1.17 (0.954,1.43).

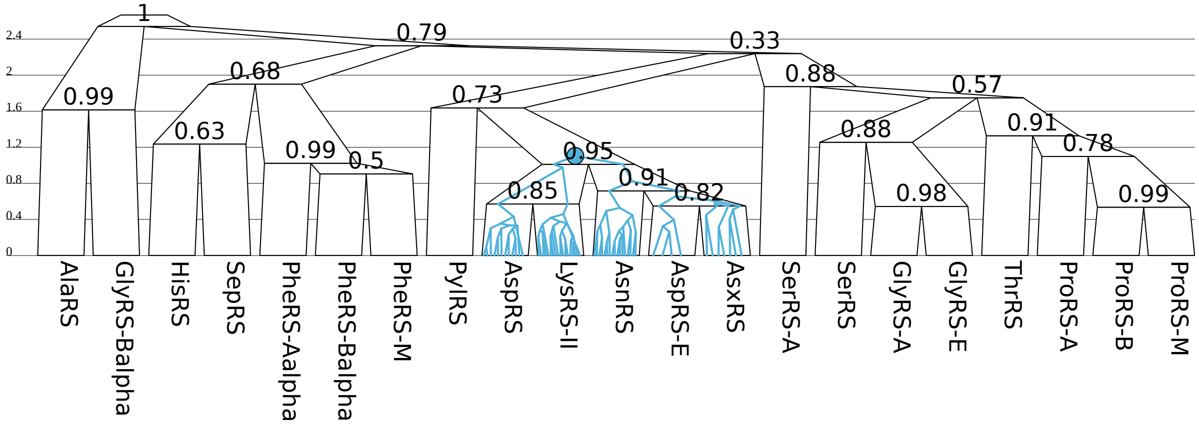


Fig. S64: **IIb insertion module phylogenies**. stimated substitution rates - IIb IM1: 1.22 (1.06,1.38); IIb IM2: 0.795 (0.668,0.91).

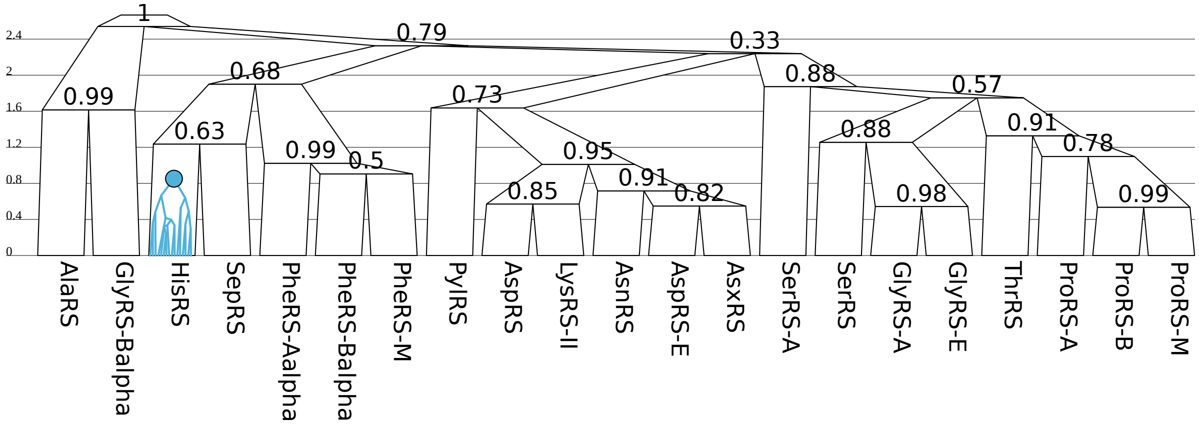


Fig. S65: **HisRS insertion module phylogeny**. Estimated substitution rate: 0.956 (0.827,1.08).

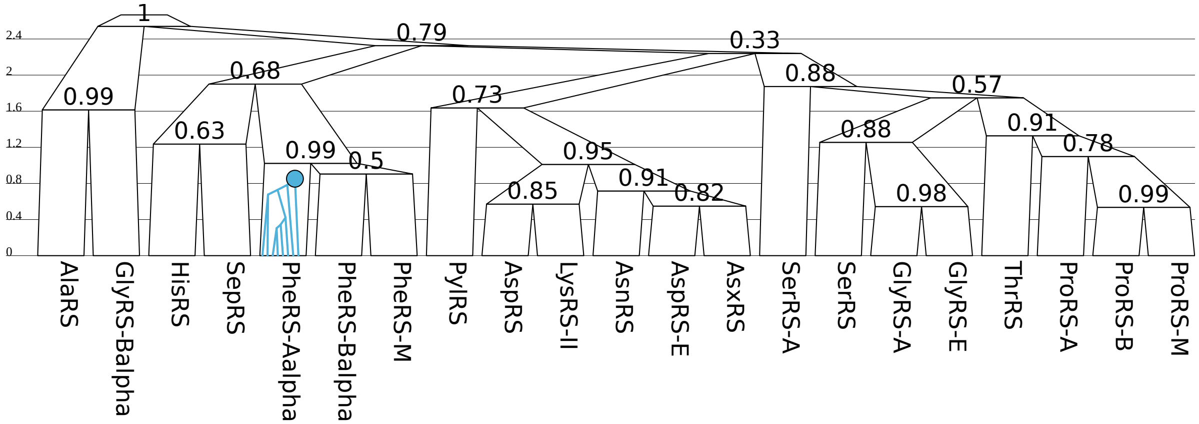


Fig. S66: **PheRS-A insertion module phylogeny**. Estimated substitution rate: 1.29 (0.943,1.66).

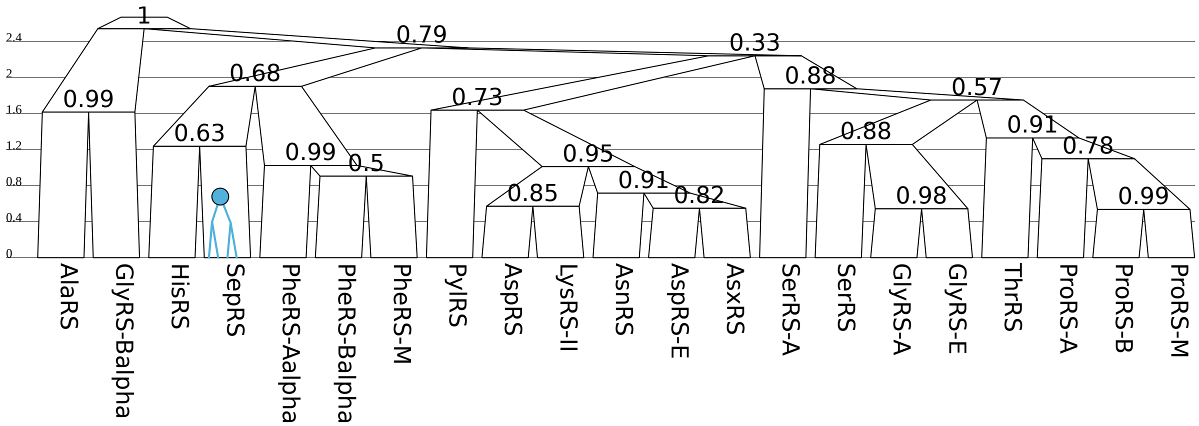


Fig. S67: **SepRS insertion module phylogeny**. Estimated substitution rate: 0.731 (0.538,0.905).

# References


[1] Bouckaert R, et al. (2014) BEAST 2: a software platform for Bayesian evolutionary analysis. PLOS Comput Biol 10(4):e1003537.

[2] Heled J, Bouckaert RR (2013) Looking for trees in the forest: summary tree from posterior samples. BMC Evolutionary Biology 13(1):221.

[3] Douglas J, Zhang R, Bouckaert R (2021) Adaptive dating and fast proposals: Revisiting the phylogenetic relaxed clock model. PLoS computational biology 17(2):e1008322.

[4] Wang S, Ma J, Peng J, Xu J (2013) Protein structure alignment beyond spatial proximity. Scientific reports 3(1):1–7.

[5] Müller T, Vingron M (2000) Modeling amino acid replacement. Journal of Computational Biology 7(6):761–776.
